# Beyond Morphological Conservatism: Integrative Taxonomy Unveils Remarkable Cryptic Diversity in the *Tachysurus virgatus* (Oshima 1926) Group (Siluriformes: Bagridae) Endemic to the Northern Margin of East Asian Tropics †

**DOI:** 10.3390/ani16152422

**Published:** 2026-08-05

**Authors:** Weihan Shao, Xingwei Cai, Tao Dinh Nguyen, E Zhang

**Affiliations:** 1 Hainan Academy of Ocean and Fisheries Sciences, Haikou 571126, China; shaoweihan1008@163.com; 2 Institute of Hydrobiology, Chinese Academy of Sciences, Wuhan 430072, China; 3 Institute of Biology, Vietnam Academy of Science and Technology, Hanoi 100000, Vietnam; dinhtao23@gmail.com

**Keywords:** catfishes, widely spread species, molecular–morphology discordance, species description, ecological divergence

## Abstract

The northern margin of the East Asian tropics harbors exceptional endemism, yet its freshwater fish fauna remains poorly characterized. *Tachysurus virgatus*, long considered a single widespread species in this region, exemplifies this taxonomic gap. Through integrative taxonomic analyses combining morphological and molecular evidence, we uncovered remarkable cryptic diversity within this species. Phylogenetic reconstructions reveal that true *T. virgatus* is restricted to the Wanquan River basin on Hainan Island, whereas nine additional lineages from other drainages represent previously unrecognized species described in this study. Together with two known congeners (*T. spilotus* and *T. kyphus*), these form the monophyletic *T. virgatus* species group for which we provide morphological diagnoses. Pronounced incongruence between morphological and molecular divergence characterizes this group, underscoring the necessity of integrative approaches for species delimitation in morphologically conserved lineages. Moreover, distinct habitat specialization patterns were identified within the group, with species exhibiting adaptations to three ecological niches: mainstem river, montane streams, and generalist habitats occupying both. Correlations between habitat preferences and functional morphology were preliminarily discussed. Using our revised taxonomic framework, we evaluated the conservation status of all species under IUCN Red List criteria, with several newly described taxa warranting threatened status due to extremely limited distributions. This study highlights the pervasive cryptic diversity of freshwater fishes in the north margin of East Asian tropics and emphasizes the critical importance of rigorous taxonomic resolution to guide effective conservation in this biogeographically complex region.

## Simple Summary

The northern margin of the East Asian tropics is a biodiversity hotspot, yet many freshwater fishes remain poorly studied. *Tachysurus virgatus*, a striped catfish long considered a single widespread species, is one example. By integrating molecular evidence with detailed morphological comparisons, the results reveal that this taxon actually comprises multiple distinct species, many of which are restricted to narrow distributions in southern China and Vietnam and are formally described in this study. Different species also prefer different habitats, such as fast-flowing mountain streams or larger lowland rivers. Recognizing this hidden diversity is essential for understanding regional biodiversity and for conservation, as several newly identified species have very limited distributions and may face greater extinction risks than previously recognized.

## 1. Introduction

The northern margin of the East Asian tropics constitutes a narrow zonal region extending nearly 2000 km from central–northern Vietnam eastward to the southern Guangxi, Guangdong, and Fujian Provinces, including Hainan Island in southern China [1]. Situated along the coastal zone of southern East Asia, this region experiences a moist, warm seasonal climate and delineates the boundary between the Indo-Burmese tropical and East Asian subtropical biogeographic provinces [2,3]. The distinctive coastal climate has persisted since the late Eocene [4], fostering exceptionally high levels of endemism across multiple taxonomic groups [5]. This region represents one of the major centers of plant and mammal species richness in East Asia [6] and harbors particularly high freshwater fish endemism, including genera such as *Cranoglanis* Peters 1881, *Metzia* Jordan & Thompson 1914, *Parazacco* Chen 1982, *Tanichthys* Lin 1932 and *Liniparhomaloptera* Fang 1935 [7].

The region’s complex topography encompasses numerous mountain ranges including the Yunkai, Shiwan, Ailao, Nanling, and Wuyi Mountains, shaped by both historical and ongoing tectonic processes. While major rivers such as the Pearl and Red rivers traverse the region, a distinctive feature of its freshwater network is the presence of numerous isolated coastal river systems, such as the Fangcheng River in Guangxi, the Moyang River in Guangdong, and the Gianh River in central Vietnam. Fluctuating sea levels in the South China Sea since the Miocene caused the intermittent connection among these river systems [8], creating opportunities for dispersal followed by isolation and diversification in freshwater fishes. This dynamic biogeographic history, combined with the general morphological conservatism exhibited by many East Asian freshwater fish lineages, has resulted in widespread cryptic diversity, where multiple species are lumped under single morphologically defined taxa [9,10]. The northern tropical margin exemplifies this pattern. For example, the White Cloud Mountain Minnow (*Tanichthys albonubes* Lin 1932), long considered a single species endemic to this region [11,12], actually comprises six cryptic species [13]. However, not all widespread taxa show deep genetic structure; some species, including *Mastacembelus armatus* Lacepède 1800 [14] and *Barbodes semifasciolatus* (Günther 1868) [15], exhibit only shallow differentiation across geographically distinct populations. These contrasting patterns indicate that different freshwater fish lineages have undergone distinct evolutionary trajectories in this region, precluding simple generalizations based on limited taxonomic sampling. Despite the high biodiversity significance of this region’s intricate river networks, cryptic diversity among widely distributed species remains poorly documented, partly due to challenges in conducting systematic transnational sampling across the national border.

However, resolving cryptic diversity has implications extending beyond taxonomic accuracy to conservation, biogeography, and evolutionary biology. Conservatively, range-restricted cryptic taxa lumped within widespread nominal species may face unrecognized extinction risks [16], obscuring conservation priorities until proper taxonomic resolution [17,18]. Biogeographically, cryptic species complexes illuminate historical processes, including the dynamic connections of drainage, historical fluctuations in sea levels, and paleoclimatic changes [19]. Evolutionarily, cryptic diversity demonstrates that reproductive isolation can proceed with minimal morphological divergence, challenging conventional understandings of diversification tempo and mode [20].

*Tachysurus virgatus* (Oshima 1926), a striped congener of *Tachysurus*, is characterized by two continuous longitudinal black stripes [21,22]. Originally described from a single 88 mm specimen collected in the Kachek River (now Wanquan River) on Hainan Island, it has traditionally been considered endemic to the northern margin of the East Asian tropics, with records spanning from central–northern Vietnam to southern Fujian Province, China [7,23,24,25]. The species occupies both major river systems and smaller isolated coastal drainages throughout its range. Despite its broad distribution across multiple river basins, *T. virgatus* sensu lato (s.l.) exhibits remarkable morphological uniformity, a pattern commonly observed in bagrid catfishes [26,27]. The common morphological conservatism, combined with the allopatric distribution of populations across independent drainage systems, raises the possibility of cryptic speciation driven primarily by geographic isolation rather than ecological divergence [28,29]. Under such scenarios, species divergence may proceed with minimal morphological differentiation due to similar selective pressures across drainages, in contrast to sympatric species that may undergo character displacement through niche partitioning. Given these characteristics, broad distribution across isolated drainages, morphological conservatism, and occurrence in a biogeographically complex region with a dynamic geological history, *T. virgatus* s.l. represents an ideal system for investigating cryptic diversity along the northern tropical margin of East Asia.

Furthermore, a recent integrative taxonomic study demonstrated that *T. virgatus* s.l. clusters with *T. kyphus* (Mai 1978), a species characterized by three broad vertical brown blotches, forming a separate lineage within *Tachysurus* [10]. The phylogenetic placement of *T. spilotus* Ng 2009, a striped congener endemic to central Vietnam and described solely on morphological grounds, remains unresolved. This species shares key morphological traits with *T. virgatus* s.l., including a relatively short maxillary barbel extending only slightly beyond the pectoral spine base and a prominent notch in the dorso-lateral stripe near the post-dorsal fin region. However, *T. spilotus* can be distinguished from both *T. virgatus* s.l. and *T. kyphus* by its deeply forked caudal fin bearing two irregular black patches on the lobes rather than continuous stripes. Molecular data are therefore needed to determine whether *T. spilotus* belongs to the monophyletic lineage comprised of *T. virgatus* s.l. and *T. kyphus*.

In this study, specimens identified as *T. virgatus* s.l. were collected from twenty-one localities across twelve river systems in southern China and central–northern Vietnam. Topotypes of *T. kyphus* and *T. spilotus*, which were previously suggested to be closely related to *T. virgatus* s.l. according to molecular and morphological evidence, were also collected. By integrating morphological and molecular analyses, this study aims to achieve two main objectives: (1) to provide robust and comprehensive morphological and molecular boundaries of the *T. virgatus* group and (2) to reveal cryptic diversity within the widespread *T. virgatus* s.l. and identify diagnostic traits of candidate species for formal description.

## 2. Methods and Materials

### 2.1. Samples Collection and Preservation

**Figure 1:**
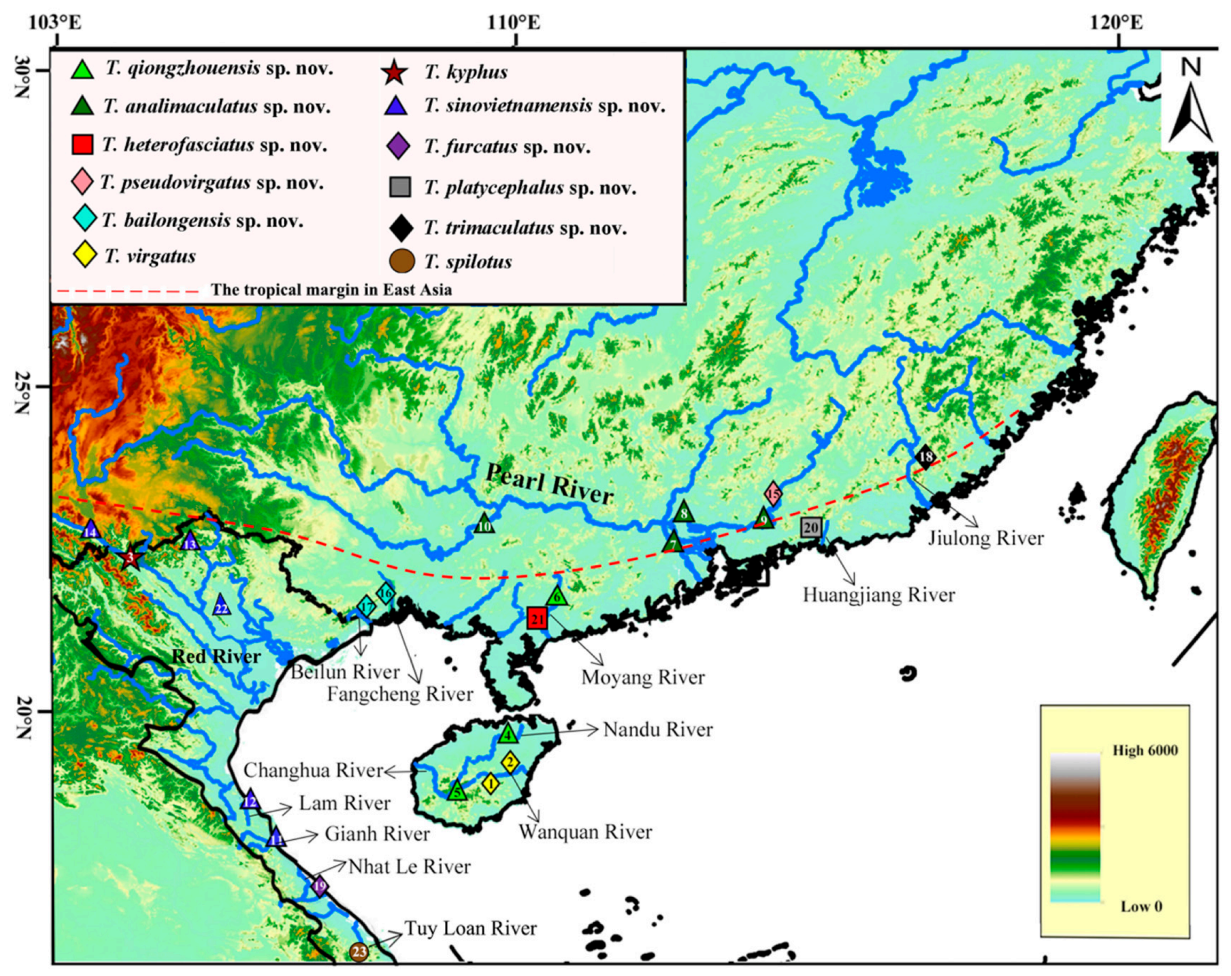
Map of sampling localities of the *Tachysurus virgatus* group. Different colors and symbol shapes correspond to the same species and morpho-groups.

Twenty-one sampling locations were selected throughout most of the historically known range of *T. virgatus* s.l. encompassing the two large river systems, the Pearl River and the Red River, as well as eleven coastal rivers along the north margin of tropics in East Asia. *Tachysurus kyphus* and *T. spilotus* are endemic to the upper reaches of the Red River and to coastal rivers in central Vietnam, respectively, and were likewise included in the present sampling. In total, the sampling localities were numbered consecutively from one to twenty-three (Figure 1, Table 1). A total of 135 individuals currently assigned to *T. virgatus* s.l. were examined in this study, together with six individuals of *T. kyphus* and two individuals of *T. spilotus*. Of these, 141 specimens were used in the morphological analyses (Table 1), whereas 43 specimens were included in phylogenetic analyses (Table 2). In addition, sequence data from 16 other congeneric species were incorporated into the phylogenetic analysis (Table 2).

### 2.2. DNA Extraction, Amplification and Sequencing

Genomic DNA was extracted from ethanol-preserved fin tissues with the DNeasy Tissue Kit (Qiagen, Beijing, China). Two mitochondrial loci, cytochrome b (*cyt-B*) and cytochrome c oxidase subunit I (COI), were subsequently amplified. In the genus *Tachysurus*, nuclear genes have been reported to evolve substantially more slowly than mitochondrial genes and to exhibit strong conservation, providing limited resolution at shallow phylogenetic levels [10]. Therefore, nuclear markers were not included in the present phylogenetic analyses.

Primer sequences and PCR cycling conditions followed those reported by Shao et al. 2021 [30]. The primer pairs used were as follows: COIF (5′-CTACAATCACCGCCTAAR-3′) and COIR (5′-TAGAAGAAAGTGACAGAGCG-3′) for the COI gene; L14724 (5′-GACTTGAAAAACCACCGTTG-3′) and H15915 (5′-CTCCGATCTCCGGATTACAAGAC-3′) for the cyt b gene. PCR amplifications were performed under the following thermal cycling conditions: an initial denaturation at 95 °C for 5 min; followed by 35 cycles of denaturation at 95 °C for 30 s, annealing at 52 °C for 45 s, and extension at 72 °C for 90 s; with a final extension at 72 °C for 8 min.

PCR products were purified and subjected to bidirectional sequencing, which was carried out by Aolinuoke Sequencing Company. Newly obtained sequences were deposited in GenBank (Table 2). Multiple sequence alignments were generated in MEGA v7.0 using MUSCLE [31] under default parameters, based on the corresponding amino acid translations. Haplotypes with more than one ambiguous site were reconstructed using PHASE v2.1.1 [32], with input files formatted in SEQPHASE [33]. Each locus was phased in three independent runs under default options. To detect the largest non-recombining fragment within each locus, recombination tests were performed with IMGC [34].

### 2.3. Phylogenetic Analyses

COI and *Cyt-B* sequences from each specimen were concatenated for phylogenetic analyses using Bayesian inference (BI) and Maximum Likelihood (ML). The GTR + I + G model was selected as the best-fit substitution model by jModelTest2 [35] under the Akaike Information Criterion [36]. *Tachysurus trilineatus* (Zheng 1979) served as the outgroup. BI analyses were conducted in MrBayes v3.1.2 [37] with three independent runs of four Markov chains each, run for 20 million generations and sampled every 1000 generations, discarding the first 25% as burn-in. Convergence was confirmed by the average standard deviation of split frequencies and the potential scale reduction factor, and MCMC traces were inspected in TRACER v1.5. ML analyses were performed in RAxML-VI-HPC [38] with 1000 bootstrap replicates.

### 2.4. Morphological Analysis

Counts and measurements followed Cheng et al. (2008) [39]. All measurements were taken point-to-point with a dial caliper and recorded to 0.1 mm; a total of 26 characters were measured. Head length and other body proportions were expressed as percentages of standard length (SL), while head subunits were given as percentages of head length (HL). Counts of dorsal- and anal-fin rays followed [40], whereas other fin rays were counted under a binocular dissecting microscope with transmitted light. Vertebral counts were obtained from X-ray images, excluding the anterior five vertebrae that form the Weberian complex.

To account for allometric effects and differences in sample size, morphometric data (excluding standard length) were size-adjusted according to Reist (1985) using the formula [41]:

M_trans_ = log *M* − b(log *BL* − log *BL*_m_) (1)

where M_trans_ is the size-transformed measurement for each individual; M is the original measurement; b is the allometric coefficient (calculated as the slope of log M against log *BL*); BL is the standard length of the individual; and BL_m_ is the mean standard length of one population while log is the base 10 logarithm. The 3D-PCA was performed and visualized with MATLAB R2024b (Mathworks Inc., Natick, MA, USA) while the 2D-PCA was run and visualized with SPSS 16 (SPSS, Chicago, IL, USA). Pairwise differences were tested using Student’s *t*-test, and variance among multiple groups was assessed via one-way ANOVA.

## 3. Results

### 3.1. Phylogeny and Geographic Distribution

After alignment and trimming the ends, a total of 2568 base pairs (bp) (COI 1479 bp and *cyt-B* 1089 bp) after alignment and trimming the ends were obtained for all forty sampled specimens recognized as *T. virgatus* s.l., *T. kyphus* and *T. spilotus*. Out of these sites in the combined mtDNA dataset, 376 were variable, and 291 were parsimony-informative.

**Figure 2:**
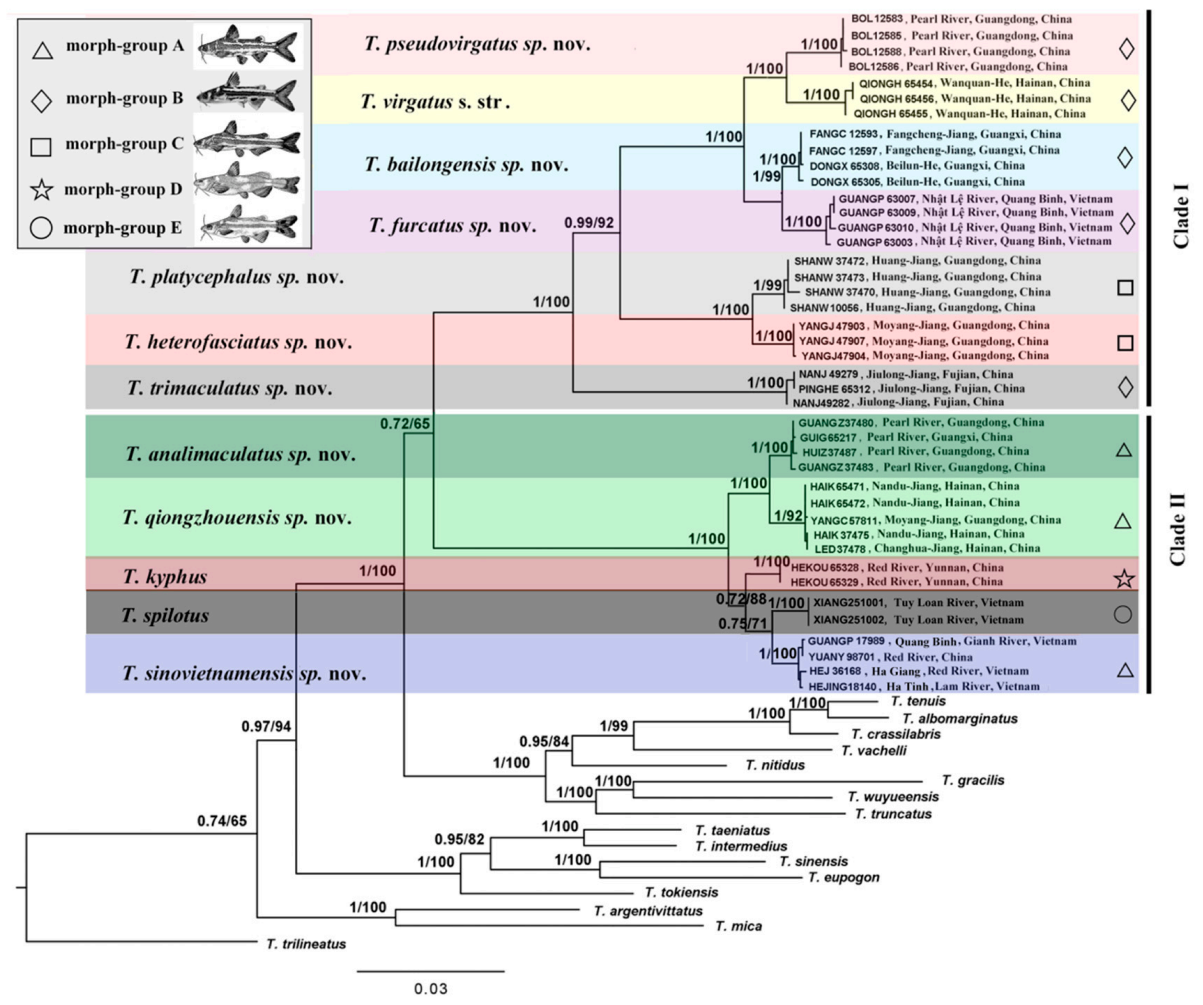
Bayesian tree based on combined genes for the *T. virgatus* species group. Values on the left of the nodes indicate posterior probabilities and bootstrap supports of Bayesian inference/maximum likelihood for major lineages. The results of morph-group subdivision are shown on the right.

Both maximum likelihood (ML) and Bayesian inference (BI) analyses recovered identical overall topologies (Figure 2). All examined specimens of *T. virgatus* s.l., *T. spilotus* and *T. kyphus* formed a strongly supported monophyletic group (PP = 100%, BP = 100%), hereafter termed the *T. virgatus* group. This group comprises two deeply divergent clades (Clades I and II) separated by uncorrected p-distances of 11.6–15.6%. Clade I contains seven allopatric lineages, each with high nodal support (PP ≥ 0.95, BP ≥ 95). Specimens from the type locality of *T. virgatus* (Wanquan River, Hainan Island) formed a distinct lineage corresponding to *T. virgatus* sensu stricto (s. str.). The remaining six lineages represent novel species described in this study.

The phylogenetic relationships within Clade I are as follows: *T. trimaculatus* sp. nov., endemic to the Jiulong River in Fujian Province, is the earliest-diverging lineage (BP = 100%, PP = 100%) and sister to all remaining species. The other six species form a well-supported clade (BP = 92%, PP = 99%) with the following structure: *T. heterofasciatus* sp. nov. and *T. platystomus* sp. nov., restricted to the Moyang River and Huangjiang River (two coastal rivers in Guangdong Province), respectively, form a robustly supported species pair (BP = 100%, PP = 100%), that is sister to a clade containing the remaining four species. Within this latter clade, *T. bailongensis* sp. nov. (endemic to Fangcheng and Beilun rivers, Guangxi, China) pairs with *T. furcatus* sp. nov. (Nhat Le River, central Vietnam) (BP = 99%, PP = 100%), and this pair is sister to *T. virgatus* s. str. + *T. pseudovirgatus* sp. nov. (BP = 100%, PP = 100%). *T. pseudovirgatus* is endemic to the Dong River, a Pearl River tributary in Guangdong Province.

The seven Clade I species exhibit divergent ecological specializations. Five species (*T. trimaculatus*, *T. heterofasciatus*, *T. platystomus*, *T. furcatus*, and *T. pseudovirgatus*) are lotic specialists restricted to stream environments. In contrast, *T. virgatus* and *T. bailongensis* are habitat generalists that primarily inhabit river main stems but also occur in stream habitats.

Clade II comprises five well-supported lineages, including *T. kyphus*, *T. spilotus* and three species previously misidentified as *T. virgatus*. Within this clade, *T. spilotus* and *T. sinovietnamensis* sp. nov. form a supported species pair (BP = 82%, PP = 92%), with *T. spilotus* endemic to coastal rivers in southern central Vietnam and *T. sinovietnamensis* occurring in the Red River and adjacent systems (Lam and Gianh rivers) in northern central Vietnam. This pair is sister to a clade containing *T. kyphus*, *T. qiongzhouensis* sp. nov., and *T. analimaculatus* sp. nov. These three species are allopatrically distributed: *T. kyphus* is endemic to the upper Red River, *T. qiongzhouensis* occurs in the Nandu and Changhua rivers on Hainan Island as well as the Moyang River in Guangdong, and *T. analimaculatus* is confined to the Pearl River basin in Guangdong and Guangxi. Ecologically, *T. kyphus* and *T. spilotus* are montane stream specialists, whereas the remaining three Clade II species are mainstem river specialists.

Uncorrected p-distances based on the *Cyt-B* gene are summarized in Table 3. Within Clade I, interspecific divergences range from 1.5% between *T. furcatus* and *T. bailongensis* to 9.2% between *T. trimaculatus* and *T. platystomus*. In Clade II, interspecific distances vary from 0.9% between *T. spilotus* and *T. sinovietnamensis* to 3.9% between *T. sinovietnamensis* and *T. analimaculatus*. Intraspecific divergences within each species are uniformly low (ranging from 0 to 0.1%).

### 3.2. Morphological Diagnosis

#### 3.2.1. Morph-Group Delineation

**Figure 3:**
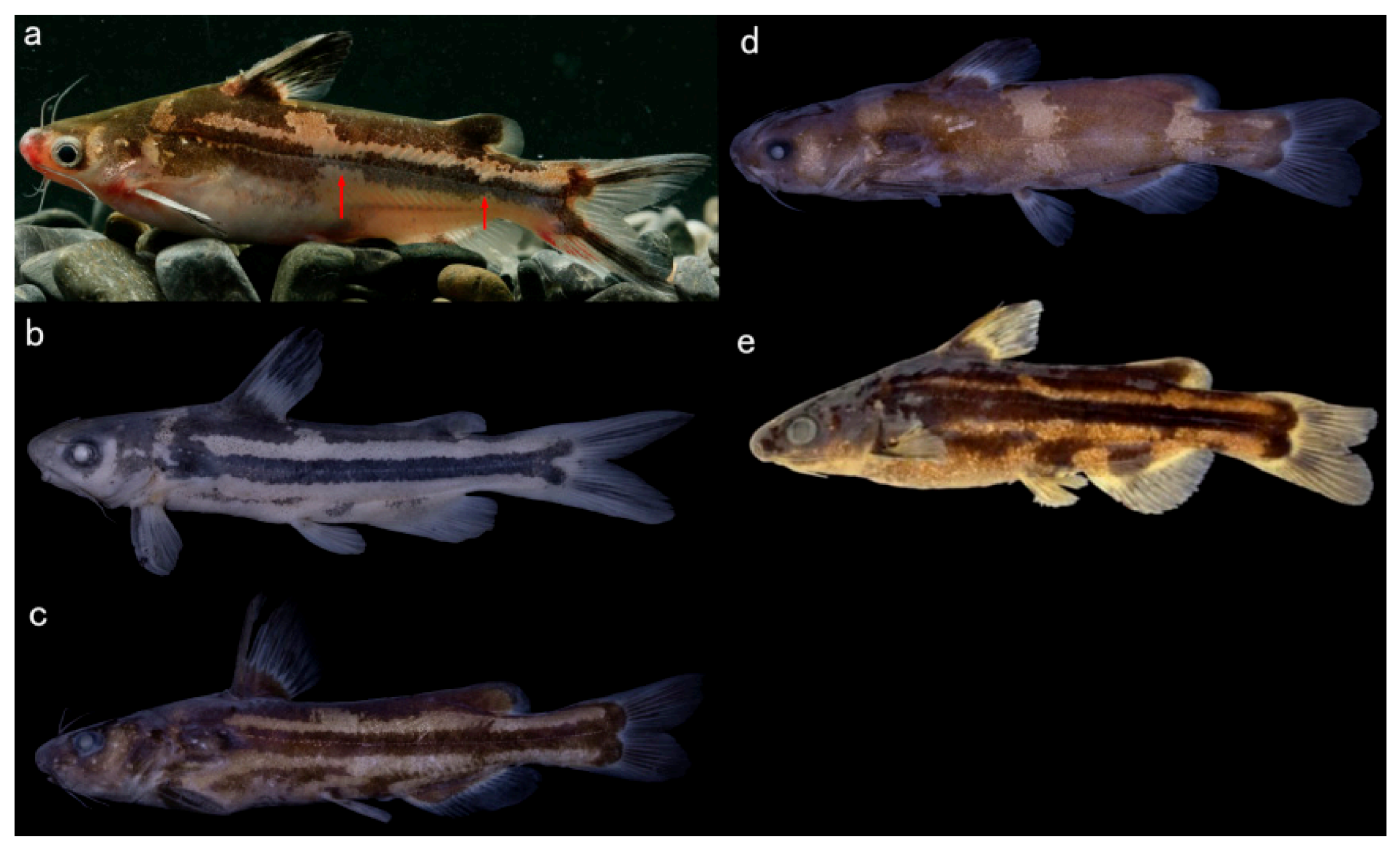
Five morph-groups within the *Tachysurus virgatus* group. (**a**) *Tachysurus sinovietnamensis* (morph-group A); (**b**) *T. virgatus* (morph-group B); (**c**) *T. platystomus* (morph-group C); (**d**) *T. kyphus* (morph-group D); (**e**) *T. spilotus* (morph-group E). Red arrow shows the two constrictions in the mid-lateral stripe of morph-group A.

Three caudal fin morphologies are present within the *T. virgatus* group: (1) deeply forked (in which the shortest branch is shorter than half the length of the longest branch) with narrow caudal-fin lobes and straight posterior edges (Figure 3a,b); (2) deeply forked with wide caudal-fin lobes and convex posterior edges (Figure 3e) and (3) moderately forked (in which the shortest branch exceeds half the length of the longest branch) (Figure 3c,d). Three body coloration patterns also occur: (1) two continuous black longitudinal stripes of uniform width (Figure 3b,c); (2) two continuous black longitudinal stripes that are constricted at two points and taper posteriorly (Figure 3a); and (3) three broad vertical brown blotches (Figure 3d). Based on the combination of the caudal-fin shape and body coloration patterns, five morph-groups are recognized within the *T. virgatus* group (Figure 3).

*Tachysurus kyphus* and *T. spilotus* fall within Clade II in the phylogenetic analysis, each representing a distinct morph-group (D and E, respectively) based on unique diagnostic traits (Figure 2). *T. kyphus* possesses a moderately forked caudal fin and can be distinguished from all other members of the *T. virgatus* group by its distinctive coloration: three broad vertical brown blotches along the body, rather than the two continuous black stripes in *T. spilotus* and *T. virgatus* s.l. *T. spilotus* is further distinguished by its highly specialized caudal fin morphology, featuring a deeply forked structure with broad lobes and convex posterior margins. Additionally, the caudal fin of *T. spilotus* bears two irregular black patches on the lobes (Figure 3e), unlike the continuous stripes extending across the caudal fin in *T. virgatus* s.l. and *T. kyphus*.

The remaining species previously treated as *T. virgatus* s.l. are allocated to three morph-groups (A–C) distributed across the two major clades. Morph-groups B and C fall within Clade I, while morph-group A belongs to Clade II alongside *T. kyphus* and *T. spilotus*. Morph-group C, comprising *T. platystomus* and *T. heterofasciatus*, is distinguished from all other morph-groups by its higher vertebral count (35–36 vs. 30–34; Table 4) and moderately forked (rather than deeply forked) caudal fin, despite sharing the two continuous horizontal black stripes typical of morph-groups A and B. Morph-groups A and B share similar overall morphology and both possess deeply forked caudal fins with two continuous horizontal black stripes. Morph-group A (*T. qiongzhouensis*, *T. analimaculatus*, and *T. sinovietnamensis*) is distinguished by a mid-lateral stripe that is constricted at two points, the posterior end of the dorsal fin and the adipose fin, and tapers posteriorly, being substantially broader at the pelvic-fin insertion than at the caudal peduncle (Figure 3a). In contrast, morph-group B (*T. virgatus*, *T. trimaculatus*, *T. bailongensis*, *T. furcatus* and *T. pseudovirgatus*) exhibits an unconstricted mid-lateral stripe of uniform width throughout its length (Figure 3b).

#### 3.2.2. Morph-Group A

**Figure 4:**
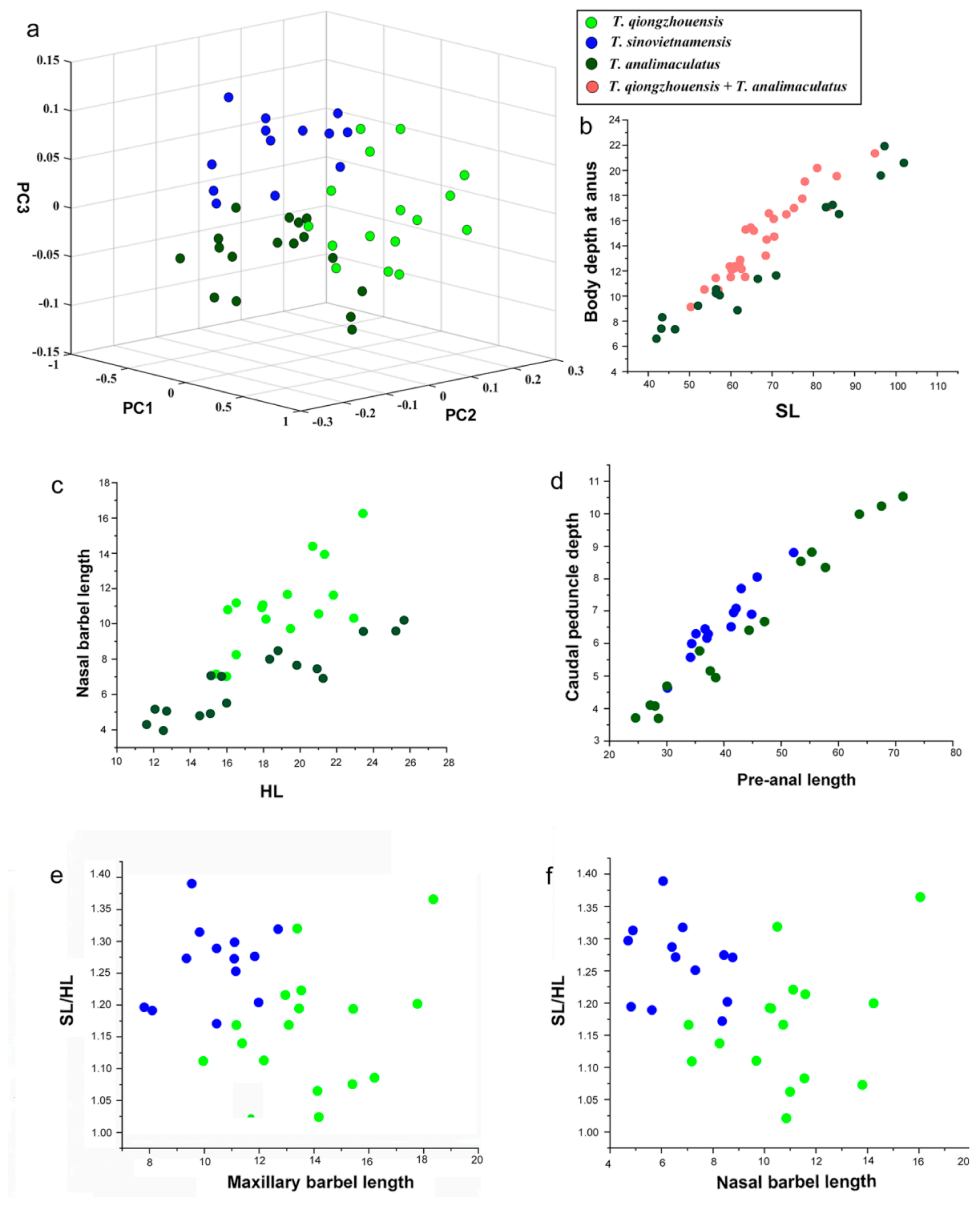
Morphometric analyses of morph−group A. (**a**) Three−dimensional principal component analysis (PCA) of the morphometric characteristics of morph-group A; (**b**) relation between body depth at anus and SL; (**c**) relation between nasal barbel length and HL; (**d**) relation between caudal peduncle depth and pre−anal length; (**e**) relation between SL/HL and maxillary barbel length; and (**f**) relation between SL/HL and nasal barbel length.

Although traditional morphometric ratios and coloration patterns do not clearly separate the three species within morph-group A, multivariate analysis reveals subtle morphological differentiation. A three-dimensional scatterplot of the first three principal components (PC1-3) from linear measurements separates the three species into largely distinct, slightly overlapping morphological spaces (Figure 4a). PC1 (86.24% of variance) loaded strongly on body depth, nasal barbel length, and caudal peduncle depth; PC2 (6.26%) on nasal and maxillary barbel length; and PC3 (1.50%) on body depth, caudal peduncle length, and inner and outer mandibular barbel lengths. Several characters contributing strongly to PC axes exhibit consistent interspecific variation. *T. analimaculatus* differs from both *T. qiongzhouensis* and *T. sinovietnamensis* in having a more slender body (depth at anus 13.8–19.2% SL vs. 19.1–24.5%) (Figure 4b). It further differs from *T. qiongzhouensis* in nasal barbel length (30.6–44.8% HL vs. 44.1–69.8%) (Figure 4c) and from *T. sinovietnamensis* in caudal peduncle depth (15.2–17.6% pre-anal length vs. 12.6–15.7%) (Figure 4d). *T. qiongzhouensis* and *T. sinovietnamensis* are morphologically conservative and cannot be reliably distinguished using conventional proportional measurements. However, bivariate plots of body proportions (SL/HL) against maxillary and nasal barbel lengths clearly separate these species into non-overlapping morphological spaces (Figure 4e,f).

#### 3.2.3. Morph-Group B

**Figure 5:**
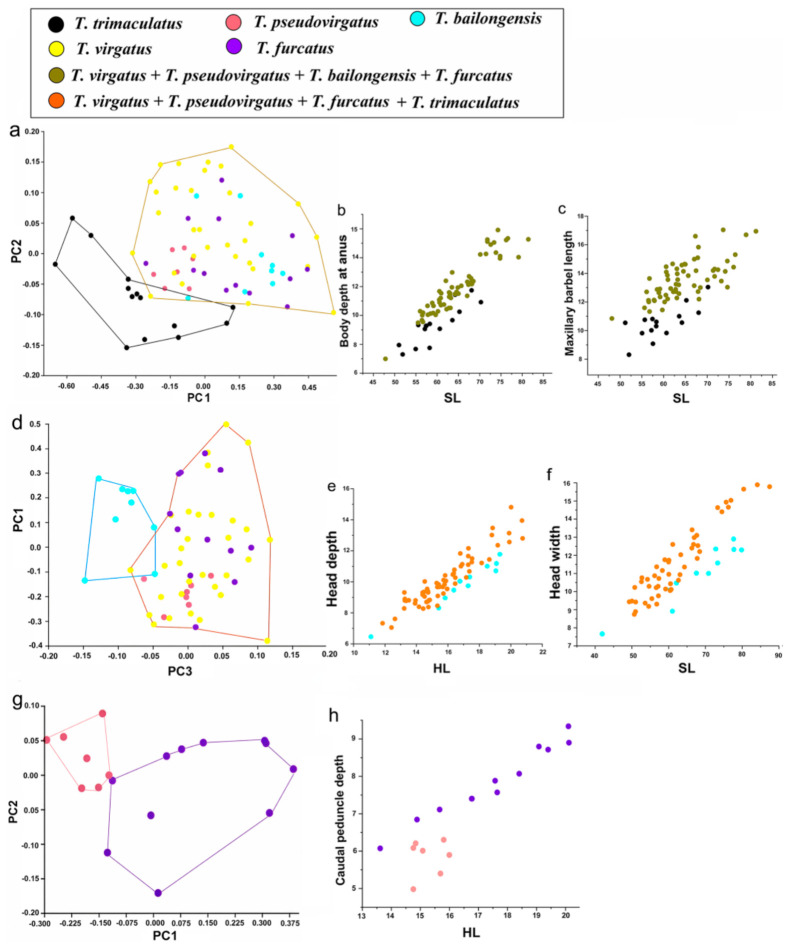
Morphometric analyses of morph−group B. (**a**) Scatter plot of PC1 against PC2 extracted from morphometric data for the species within the morph-group B; (**b**) relation between body depth at anus and SL; (**c**) relation between maxillary barbel length and SL; (**d**) scatter plot of PC1 against PC3 extracted from morphometric data for the species within the morph-group B except *T. trimaculatus*; (**e**) relation between head depth and HL; (**f**) relation between head width and SL; (**g**) scatter plot of PC1 against PC2 extracted from morphometric data for *T. pseudovirgatus* and *T. furcatus*; (**h**) relation between caudal peduncle depth and HL.

Principal component analysis for the five species within morph-group B revealed morphological differentiation. A bivariate plot of PC1 against PC2 separates *T. trimaculatus* from the other four species with slight overlap (Figure 5a). PC1 loaded strongly on body depth and caudal peduncle depth, while PC2 loaded on dorsal-spine length, nasal barbel length, and maxillary barbel length. PC3 loaded on dorsal-spine length and inner mandibular barbel length. Among the characters contributing strongly to PC axes, *T. trimaculatus* can be distinguished from the other four species by its more slender body (depth 14.0–18.4% SL vs. 18.1–21.5%; Figure 5b) and shorter maxillary barbel (16.8–20.8% SL vs. 19.0–24.6%; Figure 5c).

After excluding *T. trimaculatus*, PCA of the remaining four species reveals further differentiation. A bivariate plot of PC1 against PC3 separates *T. bailongensis* from *T. virgatus*, *T. pseudovirgatus*, and *T. furcatus* (Figure 5d). PC1 exhibited strong positive loadings across all traits and accounted for 68.84% of the total variance, representing overall body size. PC2 (7.72%) loaded primarily on nasal barbel length, while PC3 (4.99%) loaded strongly on dorsal-spine length and nasal and inner mandibular barbel lengths. *T. bailongensis* can be distinguished from the other three species by its relatively shallower head (depth 53.4–59.1% HL vs. 58.6–71.1%; Figure 5e) and narrower head (width 59.8–68.5% HL vs. 66.4–76.7%; Figure 5f).

Among the remaining three species, *T. pseudovirgatus* and *T. furcatus* show clear morphological differentiation in PCA, with PC1 vs. PC2 plot effectively separating the two species (Figure 5g). *T. pseudovirgatus* can be distinguished from *T. furcatus* by its more slender caudal peduncle (depth 42.1–45.3% HL vs. 59.5–71.1%; Figure 5h). In contrast, *T. virgatus* exhibits substantial overlap with both *T. pseudovirgatus* and *T. furcatus* in morphometric space and cannot be reliably distinguished from these species using traditional morphometric characters alone (Figure S1).

**Figure 6:**
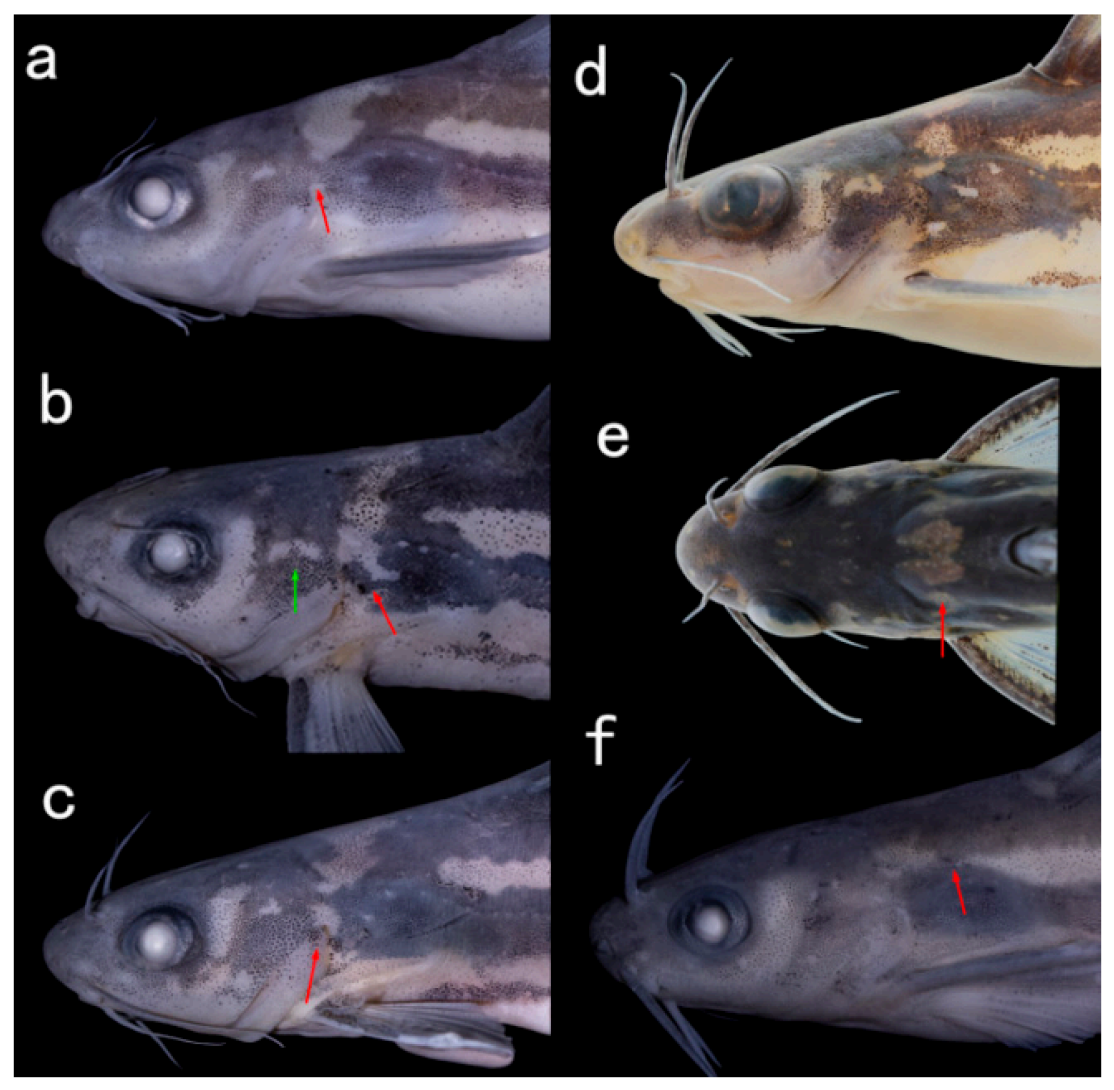
(**a**) Lateral view of the head and nuchal region of *T. pseudovirgatus* (red arrow showing the connection between the mid-lateral stripe and opercular blotches); (**b**) lateral view of the head and nuchal region of *T. pseudovirgatus* (red arrow showing the short white line interrupting the origin of the mid-lateral stripe, green arrow showing the whitish-yellow patch along the posterior margin of eyes); (**c**) lateral view of the head and nuchal region of *T. furcatus* (red arrow showing the connection between the mid-lateral stripe and opercular blotches); (**d**) lateral view of the head and nuchal region of *T. bailongensis*; (**e**) lateral view of the head and nuchal region of *T. trimaculatus* (red arrow showing the connection between dorso-lateral and mid-lateral stripes); (**f**) dorsal view of the head and nuchal region of *T. trimaculatus* (red arrow showing the connection between the dorso-lateral stripe and opercular blotches).

However, the coloration pattern at the junction between the origin of the mid-lateral stripe and the black operculum blotches can serve as a valuable diagnostic character for morphological differentiation among these three species. *T. pseudovirgatus* resembles *T. bailongensis* and *T. trimaculatus* in having the origin of the mid-lateral stripe largely continuous with the black operculum blotches (Figure 6a,d,f). In *T. virgatus*, a short, ventrally inclined yellow-whitish line interrupts the beginning of the stripe, with its lower portion extending to connect with the black blotches on the operculum (Figure 6b). In *T. furcatus*, the origin of the mid-lateral stripe diverges into two or three short black lines connecting to the operculum (Figure 6c). Furthermore, *T. pseudovirgatus* differs from *T. virgatus* in having a narrower (vs. broader) whitish-yellow patch along the posterior margin of the eyes (Figure 6a,b).

Moreover, the coloration pattern at the stripe–operculum junction also helps distinguish other morph-group B species. Both *T. trimaculatus* and *T. bailongensis* possess a connection between the dorso-lateral stripe and the opercular blotches, a feature absent in other members of the *T. virgatus* group (Figure 6e,f). These two species can be further separated by the connection of the dorso- and mid-lateral stripe connection anterior to the dorsal fin: *T. trimaculatus* exhibits only a slight or no connection between these stripes (Figure 6f), whereas *T. bailongensis* shows a broad connection (Figure 6d,e).

#### 3.2.4. Morph-Group C

**Figure 7:**
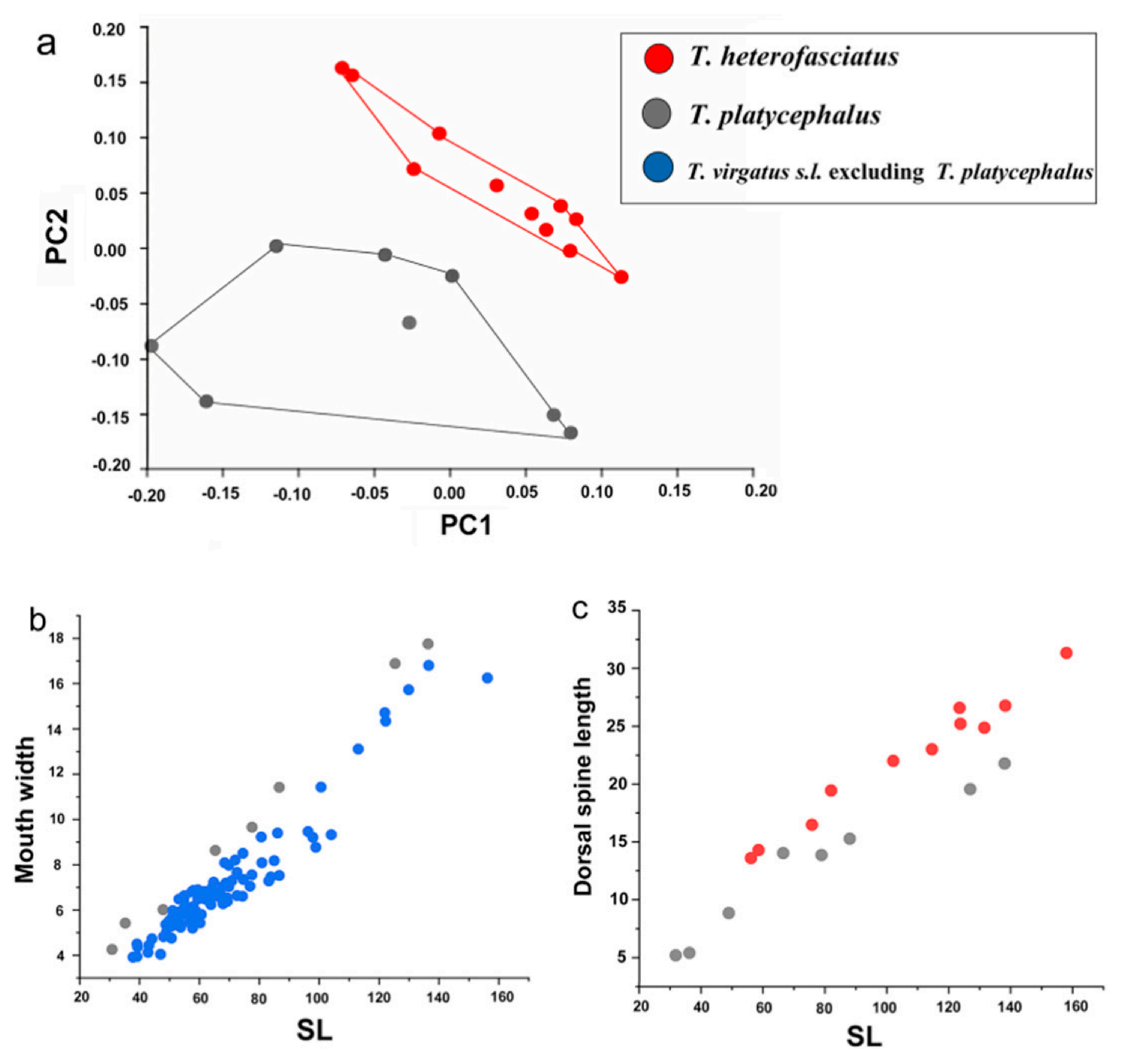
Morphometric analyses of morph−group C. (**a**) Scatter plot of PC1 against PC2 extracted from morphometric data for the species within the morph−group C; (**b**) relation between mouth width and SL; (**c**) relation between dorsal spine length and SL.

Principal component analysis of morphometric measurements for morph-group C shows that a plot of PC1 against PC2 separates *T. heterofasciatus* and *T. platystomus* into distinct clusters (Figure 7a). PC1 exhibited strong positive loadings across all traits and accounted for 97.74% of the total variance, representing overall body size. PC2 loaded primarily on dorsal-spine length, pectoral-spine length, and mouth width, while PC3 loaded strongly on nasal and maxillary barbel lengths.

*Tachysurus platystomus* can be distinguished from all other species in the *T. virgatus group* by its wider mouth (12.3–14.7% SL vs. 8.9–11.7%; Figure 7b). It further differs from *T. heterofasciatus* in dorsal-spine length (15.7–20.3% SL vs. 19.1–24.4%; Figure 7c).

Moreover, *T. heterofasciatus* differs from *T. platystomus* in coloration pattern by having a mid-lateral stripe that is distinctly (vs. slightly) constricted at the posterior ends of the dorsal and adipose fins, and a dorso-lateral stripe that extends ventrally to approach or contact the mid-lateral stripe (vs. remaining well separated from it) (Figure S2).

## 4. Discussion

### 4.1. Hidden Diversity and Species Delineation

In this study, specimens identified as *T. virgatus* s.l. or previously suggested to be closely related to *T. virgatus* s.l. were systematically sampled across their known distribution range. With the exception of *T. spilotus*, for which sample size was relatively limited due to its restricted distribution and sampling difficulty, all other species were represented by sufficiently large sample sizes. Surprisingly high levels of cryptic diversity were uncovered within the *T. virgatus* group as specimens previously assigned to *T. virgatus* s.l. were resolved into ten distinct lineages distributed across southern China and central–northern Vietnam. True *T. virgatus* is confined exclusively to the Wanquan River on Hainan Island, its type locality, whereas the remaining nine lineages represent undescribed species formally described herein. Phylogenetic analyses further reveal that *T. spilotus* and *T. kyphus*, morphologically distinctive species previously recognized based on coloration, are nested within the *T. virgatus* complex, rendering *T. virgatus* s.l. paraphyletic. This topology also indicates that the distinctive color patterns of *T. spilotus* and *T. kyphus* evolved independently within a broader radiation of morphologically conservative lineages, exemplifying the decoupling of molecular and morphological evolution in this group.

Species delineation within the *T. virgatus* group exemplify the taxonomic challenges common in catfishes as most lineage pairs fall within the “grey zone” of speciation [42], an intermediate stage where taxonomic criteria yield conflicting species delimitations due to asynchronous differentiation of diagnostic characters. Taxa within this grey zone typically exhibit mitochondrial genetic distances ranging from 0.5% to 2% [43] and/or possess fewer than two diagnostic morphological traits [44]. This “grey zone” phenomenon reflects the complex and heterogeneous dynamics of trait evolution within the *T. virgatus* group during the speciation continuum. We identified three distinct patterns of molecular–morphological evolution that inform our taxonomic decisions.

First, some species pairs show congruent molecular and morphological differentiation, with substantial genetic divergence (≥2% in *cyt-B* gene, a threshold widely used for freshwater fish delimitation) accompanied by clear morphological differentiation. For example, *T. platystomus* and *T. heterofasciatus*, distributed in separate coastal rivers in Guangdong Province, China, exhibit 2.1% genetic divergence accompanied by clear differences in morphometric traits and coloration patterns. *T. trimaculatus* and its sister species also show similar congruent differentiation. These cases conform to traditional expectations where genetic and phenotypic divergence accumulate in parallel.

In contrast, three species, *T. sinovietnamensis*, *T. qiongzhouensis*, and *T. analimaculatus*, exhibit substantial genetic divergence (1.9–2.7% pairwise *cyt-B* distances) yet remain morphologically cryptic, distinguishable only through multivariate morphometric analysis. These three species display allopatric distributions and occupy similar ecological niches, being strictly restricted to mainstem river habitats, suggesting shared selective pressures. Importantly, phylogenetic analyses reveal that these three species do not form a monophyletic group: *T. sinovietnamensis* is sister to *T. spilotus*, while *T. qiongzhouensis* and *T. analimaculatus* form a clade sister to *T. kyphus*. Under the unified species concept [42], species are separately evolving metapopulation lineages, and multiple lines of evidence, including reciprocal monophyly, allopatric distributions and genetic distinctiveness, can support the recognition of these three taxa as distinct species despite their morphological similarity. This pattern exemplifies the increasingly recognized phenomenon that speciation and morphological divergence are decoupled processes [19,45,46]. The morphological conservatism likely reflects stabilizing selection imposed by shared ecological niches, a pattern commonly observed in cryptic species complexes where niche conservatism constrains phenotypic evolution [19].

Moreover, several species pairs exhibit substantial morphological and ecological differentiation despite shallow genetic divergence, likely reflecting recent but ecologically driven speciation. The allopatric sister species *T. sinovietnamensis* and *T. spilotus* (Clade I) diverge by only 0.9% distance in *cyt-B* gene yet differ markedly in caudal fin morphology: *T. sinovietnamensis* possesses narrow, straight lobes with two short black stripes, while *T. spilotus* displays broad, convex lobes with two circular black spots. These species also occupy distinct ecological niches, *T. sinovietnamensis* inhabits lowland river mainstems, while *T. spilotus* is restricted to montane streams. Their reciprocal monophyly, allopatric distributions, morphological distinctiveness, and ecological divergence collectively support their recognition as separate species despite low genetic distance. A similar morphology–molecular pattern also occurs in Clade II, where *T. bailongensis* and *T. furcatus* exhibit only 1.5% genetic divergence yet display substantial morphological differences in both morphometric characters and coloration patterns as well as ecological niche differentiation. The low genetic distances observed between closely related species in the *T. virgatus* group might be attributed to the reduced substitution rates characteristic of Siluriformes [30]. The East Asian bagrid mitochondrial *cyt-B* gene evolves at approximately 0.18–0.30% sequence divergence per million years [47], considerably slower than rates observed in other freshwater fish groups, such as 1.5% in salmonids [48]. Under these rates, the 0.9% divergence between *T. sinovietnamensis* and *T. spilotus* corresponds to more than one million years of separation, providing ample time for morphological and ecological divergence despite modest genetic distance.

Collectively, our findings demonstrate that neither genetic distance thresholds nor morphological differentiation alone suffice for species delimitation in the *T. virgatus* group. Instead, an integrative taxonomic framework that synthesizes multiple independently evolving characters can be employed for resolving taxonomic boundaries within the *T. virgatus* group where molecular and morphological evolution are frequently decoupled, including reciprocal monophyly in multilocus phylogenetic analyses, morphological differentiation, ecological niche divergence and allopatric distributions preventing gene flow.

### 4.2. Morphological Boundary of the T. virgatus Group

Previous molecular and morphological analyses revealed that striped *Tachysurus* species comprise two distinct evolutionary lineages [10]. The *T. argentivittatus* group includes three diminutive species (*T. argentivittatus*, *T. mica* and *T. longispinalis*), characterized by a minimized body size, interrupted lateral lines, and exceptionally long maxillary barbels. In contrast, *T. virgatus* s.l., together with the unstriped species *T. kyphus*, constitutes a distinct phylogenetic clade, herein designated the *T. virgatus* group. The present study, with expanded taxonomic and geographic sampling, corroborates this framework and further demonstrates that *T. spilotus* clusters with *T. virgatus* s.l. and *T. kyphus*, together forming a well-supported monophyletic lineage. This clade is recovered as sister to a large assemblage comprising the *T. pratti-T. truncatus*, *T. tenuis-T. crassilabris*, *T. vachelli*, and *T. nitidus* groups designated by Zhou et al. (2024) and Shao & Zhang (2022) [22,49].

Members within the *T. virgatus* group exhibit considerable variation in coloration patterns, which complicates morphological diagnosis. The typical striped pattern in the *T. virgatus* group consists of two continuous longitudinal black stripes, with the mid-lateral stripe bifurcating at the caudal-fin base into two short stripes extending onto the caudal-fin lobes. However, *T. kyphus* lacks longitudinal stripes, instead displaying three black ventral blotches, while *T. spilotus* exhibits two circular black spots on the caudal-fin lobes rather than short stripes. Despite this variation, these species retain several shared synapomorphies. Most notably, all possess paired semicircular black markings positioned at the base of the caudal fin. Based on this and other shared features, we diagnose the *T. virgatus* group by the following characters: (1) adult individuals exceeding 100 mm in standard length; (2) presence of either continuous black longitudinal stripes or three ventral blotches, with paired semicircular black spots at the caudal-fin base; (3) continuous lateral line; and (4) smooth outer margin of the pectoral fin spine.

### 4.3. Ecological Differentiation Within the T. virgatus Group

Species in the *T. virgatus* group appear to exhibit pronounced habitat divergence, suggesting that niche partitioning could contribute to their sympatric occurrence [50]. For instance, in the Moyang River (Guangdong Province, China), *T. qiongzhouensis* is primarily found in lowland mainstem habitats, whereas *T. heterofasciatus* appears to be associated with montane streams. A comparable pattern may occur in the Red River basin, where *T. kyphus* is mainly distributed in upland streams and *T. sinovietnamensis* occurs predominantly in mainstem habitats. This niche divergence likely reduces interspecific competition, thereby facilitating sympatry among closely related species; however, additional ecological and behavioral data are needed to confirm this interpretation.

**Figure 8:**
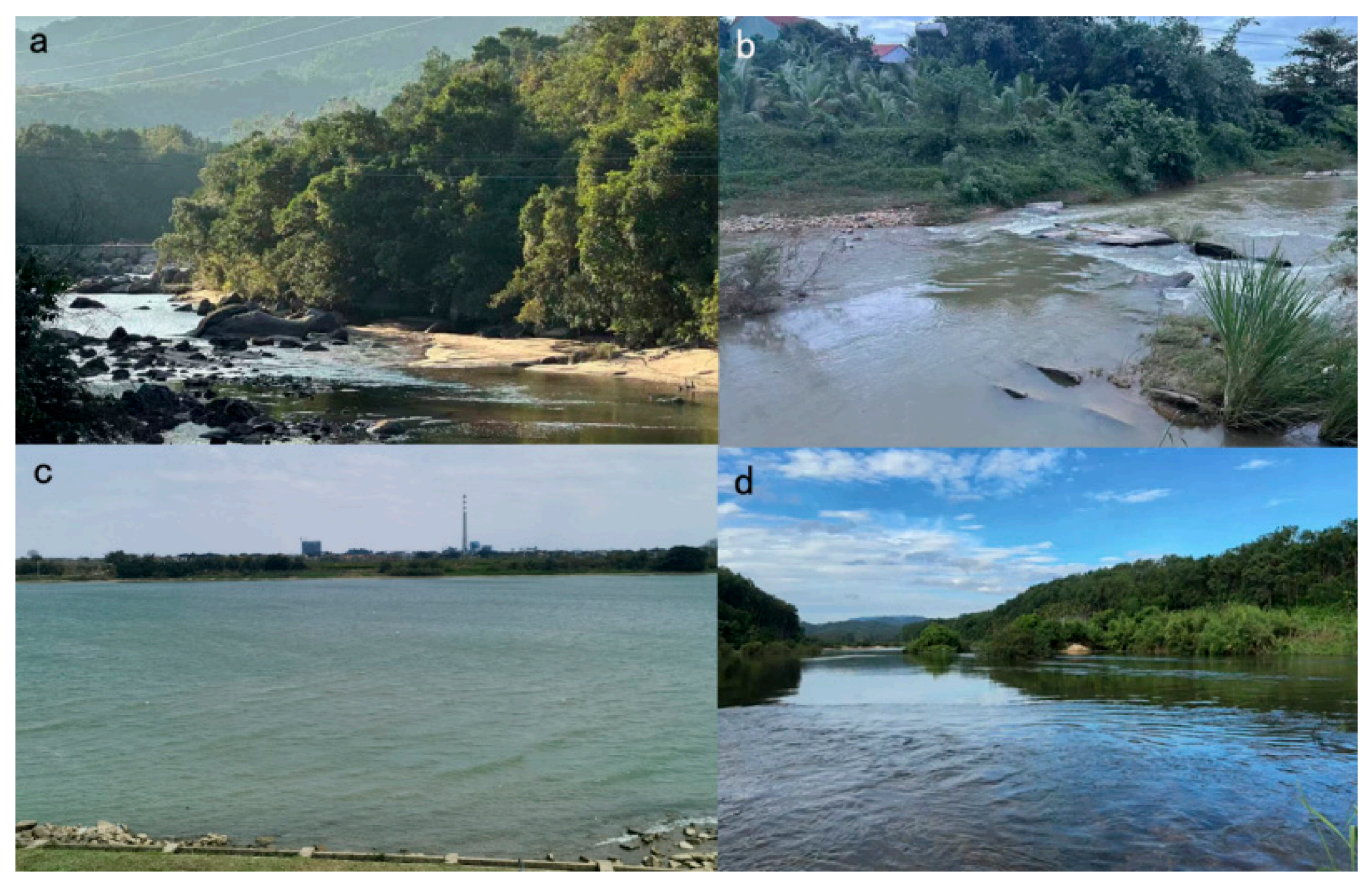
Photographs showing three typical habitat types of species in the *T. virgatus* group. (**a**) Mountain stream of the Hàn River inhabited by *T. spilotus*, (**b**) montane stream of the Moyang River inhabited by *T. heterofasciatus*, (**c**) lower reaches of the Pearl River inhabited by *T. analimaculatus*, (**d**) middle-upper reaches of the Wanquan River inhabited by *T. virgatus*.

Three distinct habitat utilization strategies exhibited among the species within the *T. virgatus* group. The majority function as habitat specialists occupying only a single habitat type with narrow niche breadth. Seven species, including *T. heterofasciatus*, *T. platystomus*, *T. kyphus*, *T. spilotus*, *T. pseudovirgatus*, *T. furcatus* and *T. trimaculatus*, are obligately stream-dwelling (Figure 8a,b), these environments are characterized by high flow velocities, coarse gravel or boulder substrates and well-oxygenated water. Conversely, three species, *T. sinovietnamensis*, *T. qiongzhouensis* and *T. analimaculatus*, are obligate mainstem specialists confined to lowland river channels (Figure 8c). These habitats feature slower currents, finer sand or silt substrates and lower dissolved oxygen concentrations. The remaining two species, *T. virgatus* and *T. bailongensis*, are habitat generalists that occupy both mainstem rivers and montane streams (Figure 8d). Field surveys suggest these species may exhibit ontogenetic habitat shifts: juveniles and subadults were predominantly collected in stream habitats, whereas adults comprised the majority of specimens from mainstem rivers. However, definitive evidence for ontogenetic habitat shifts in these taxa remains lacking, future investigations should prioritize elucidating the complete life history strategies of these species.

Moreover, based on the field investigations, mainstem specialists and generalists occupy distinctly different habitats within river mainstems. Specifically, mainstem specialists predominantly inhabit the middle-lower reaches of large river systems such as the Pearl River, Red River, and Nandu River (Figure 8c), whereas the two generalist species typically occupy the middle-upper reaches of smaller coastal rivers including the Wanquan River, Fangcheng River and Beilun River (Figure 8d). This spatial segregation corresponds to pronounced differences in hydrological characteristics, including flow velocity, discharge volume, and substrate composition. The middle-upper reaches where generalists occur are positioned closer to montane streams and characterized by gravel-dominated substrates, whereas the middle-lower reaches inhabited by specialists are predominantly composed of sand and silt [51,52]. Species distribution patterns further reflect differential habitat utilization between these two habitat types. Species occupying the middle-lower reaches of river mainstems typically exhibit broad distribution ranges and trans-basin dispersal capabilities, whereas those restricted to the middle-upper reaches often display narrower ranges likely owing to their limited dispersal capability.

Interspecific morphological variations observed in the *T. virgatus* group appear to reflect adaptations to divergent hydrodynamic conditions between montane streams and lowland mainstem habitats [53,54]. Caudal fin morphology represents a critical locomotor adaptation in fishes, as fin shape directly influences swimming performance across different flow regimes [55,56]. Among the seven montane stream specialists in this group, four species including *T. spilotus*, *T. kyphus*, *T. heterofasciatus*, and *T. platystomus*, exhibit derived caudal fin morphologies characterized by either expanded caudal-fin lobes (*T. spilotus*) or connections between caudal-fin lobes (*T. kyphus*, *T. heterofasciatus* and *T. platystomus*). The enlargement of caudal fin surface area may represent a widespread adaptation among benthic stream-dwelling fishes and is hypothesized to be a functional response to the combined selective pressures of increased flow velocity and reduced water column depth [57].

However, three montane stream specialists, namely, *T. trimaculatus*, *T. pseudovirgatus* and *T. furcatus* retain deeply forked caudal fins with narrow, elongate lobes resembling those of mainstem habitat specialists. Several non-exclusive explanations may account for this: (1) phylogenetic niche conservatism; (2) compensatory adaptations in other morphological traits that reduce selection on caudal fin shape; (3) ecological differences in micro-habitats, as these species may occupy lower-gradient stream reaches with intermediate flow velocities; and (4) recent colonization of stream habitats with insufficient time for morphological evolution. Interestingly, morphological analysis suggests that *T. trimaculatus* has evolved an alternative adaptive solution by possessing lower body depth than closely related species (Figure 5b) which can potentially trade caudal fin modification under high flow velocity pressures. Nevertheless, the precise mechanisms underlying caudal fin retention in these three species require further investigation.

Conversely, all species that occupy mainstem river habitats, whether exclusively (*T. sinovietnamensis*, *T. qiongzhouensis* and *T. analimaculatus*) or facultatively (*T. virgatus* and *T. bailongensis*), consistently possess deeply forked caudal fins with narrow, elongate lobes. This morphology may be characteristic of fishes inhabiting deeper water with slower currents and is associated with enhanced cruising efficiency and burst swimming performance [54].

Beyond fin morphology, the disruptive coloration patterns of longitudinal stripes on the body of *T. kyphus* and short stripes on the caudal fin of *T. spilotus* may represent adaptations to the visual backgrounds of montane streams. These patterns potentially provide crypsis by mimicking the gravel substrates characteristic of such habitats, thereby reducing predation risk and enhancing foraging efficiency by decreasing prey detection [58,59,60].

### 4.4. Conservation Implications

Accurate species delimitation is fundamental to conservation biology, as species serve as the primary units for threat assessment and management planning [61]. Failure to recognize cryptic diversity within widespread taxa can result in inadequate conservation prioritization, as geographically restricted or ecologically specialized lineages may face elevated extinction risks that remain undetected when populations are assessed collectively [62,63]. Taxonomic revision that reveals cryptic species complexes therefore has direct implications for conservation status assessments and necessitates re-evaluation of threat levels and development of species-specific management strategies [64,65]. The conservation significance of this revision is clear for *T. virgatus* sensu lato. Although previously treated as a widespread species and currently listed as Data Deficient by the IUCN [66], it would likely have been regarded as Least Concern based on its apparently broad distribution and presumed large population size.

Range size, abundance, and population/occurrence decline trends are key criteria used by the International Union for the Conservation of Nature (IUCN) to determine species conservation status [43]. Our taxonomic revision substantially alters conservation priorities within the group, as most of them have much narrower distributions. This result has important implications for reassessing their conservation status. *T. virgatus* s. str. is endemic to the Wanquan River basin on Hainan Island, where it remains abundant throughout the middle and upper mainstem reaches and associated streams. Similar patterns characterize other members of the *T. virgatus* group that inhabit river mainstems either exclusively or facultatively. Despite the comparatively low Extent of Occurrence (EOO) of these species (ranging from 5000 to 30,000 km^2^), no significant population declines or threats have been observed. Consequently, these species should not be considered threatened, and we recommend maintaining the LC classification based on IUCN criteria [66]. Conversely, the obligate stream-dwelling species within the *T. virgatus* group face markedly different conservation challenges. *T. kyphus* and *T. spilotus* possess relatively broad distributions and robust population sizes, with EOOs exceeding 20,000 km^2^ and presence in more than five locations, thus also warranting LC designation. *T. trimaculatus* and *T. heterofasciatus* maintain substantial populations but are confined to extremely restricted ranges, each known from a single location. However, as neither continuous population declines nor extreme fluctuations in occurrence have been documented for these species, they merit Vulnerable (VU) classification under Criterion D2. *T. platystomus* and *T. pseudovirgatus* are likewise restricted to single locations yet exhibit small population sizes. These two species occur in close proximity to the Pearl River Delta, China’s largest urban agglomeration, and have experienced pronounced population declines over the past decade due to intensive urbanization pressures. Based on these factors, they warrant classification as Endangered (EN) under Criterion B1a,b.

## 5. Taxonomic Treatments

### 5.1. Species Description

The present study establishes that the *T. virgatus* species group encompasses twelve species in total: three previously recognized species and nine undescribed species herein identified. Formal descriptions of these nine new species are detailed in Supplementary File S1 and the morphological measurement data are provided in Supplementary File S2.

***Tachysurus virgatus* (Oshima 1926)**

(Figure 3b)

Diagnosis. A striped *Tachysurus* species. Distinguished from all other striped congeners except *T. bailongensis*, *T. pseudovirgatus*, *T. trimaculatus* and *T. furcatus* by the following combination of characters: a deeply (vs. moderately) forked caudal fin, a straight (vs. gently convex) posterior edge of the caudal-fin lobes, presence (vs. absence) of a prominent post-dorsal-fin notch on the dorso-lateral stripe and a uniform and unconstricted (vs. tapering, constricted and irregular) mid-lateral stripe. It further differs from *T. bailongensis*, *T. pseudovirgatus*, *T. trimaculatus* and *T. furcatus* in absence (vs. presence) of a short whitish-yellow line in the beginning of the mid-lateral stripe.

Distribution and habitat: Currently only known from Wanquan River on Hainan Island. Primarily occupy river mainstems while also occurring in stream habitats.

***Tachysurus kyphus* (Mai 1978)**

(Figure 3d)

Diagnosis. Distinguished from all other *Tachysurus* species except *T. ichikawai* by the combination characters: a moderately forked caudal fin and three broad vertical brown blotches along the body. It can be further differentiated from *T. ichikawai* by having shorter maxillary barbel not extending (vs. extending) to the pectoral-fin base.

Distribution and habitat: Currently known from the Red River in northern Vietnam and southern Yunnan, China. Recorded exclusively in flowing streams.

*Tachysurus spilotus* Ng 2009

(Figure 3e)

Diagnosis. A striped *Tachysurus* species. Distinguished from all other striped congeners by having two black circular spots (vs. two black short stripes) on the caudal fin lobes.

Distribution and habitat: Currently known from the coastal rivers in central Vietnam including Han River and Anlao River. Recorded exclusively in flowing streams.

**Figure 9:**
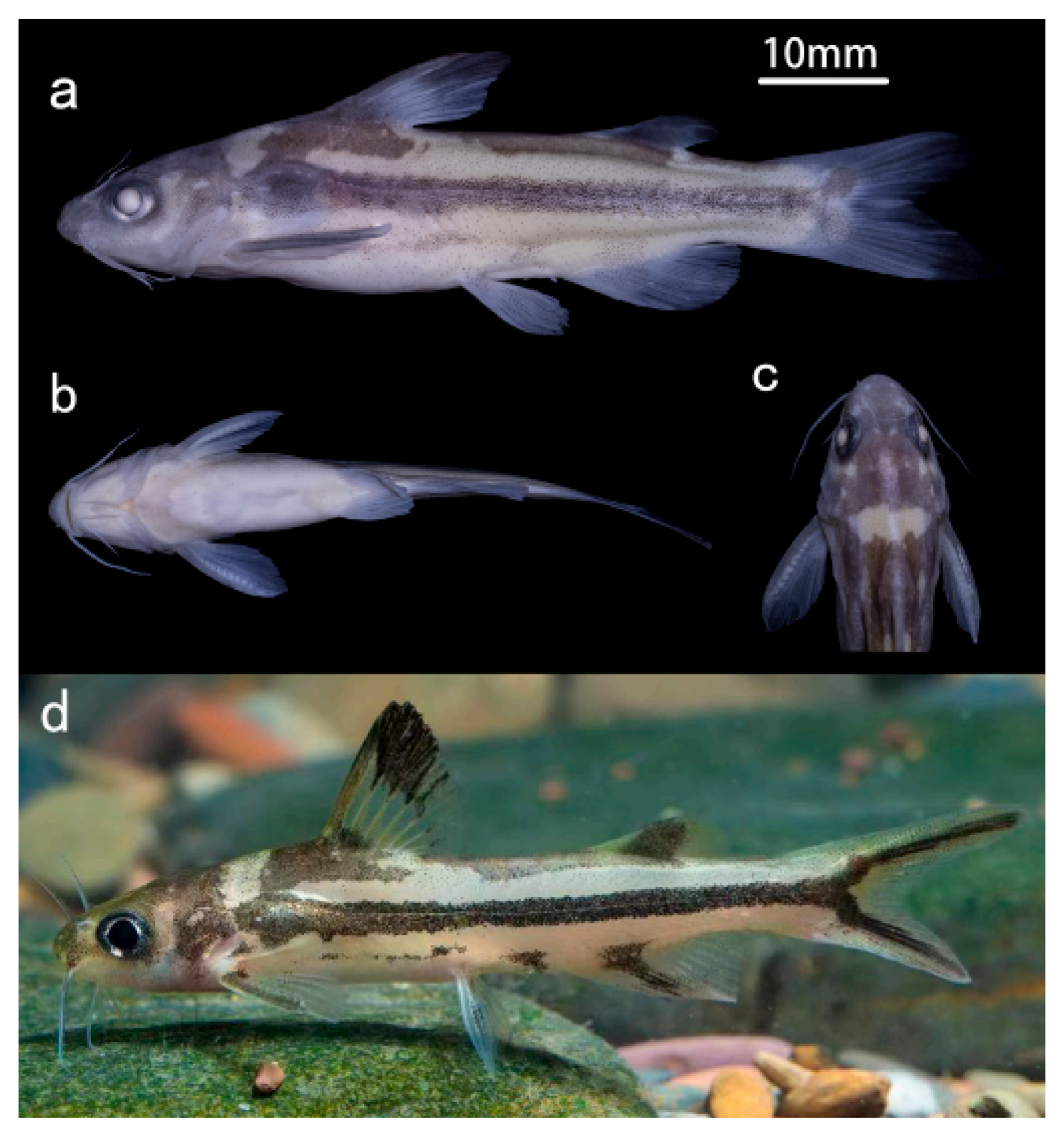
Lateral (**a**), dorsal (**b**) and ventral (**c**) views of *Tachysurus pseudovirgatus* sp. nov. (**d**) Freshly collected specimen of *Tachysurus pseudovirgatus.*

***Tachysurus pseudovirgatus* Shao & Zhang, sp. nov.** (Figure 9)

Holotype. 201909112589, 59.2 mm SL, South China: Guangdong Prov.: Boluo County: a stream in Dong River, the tributary of the Pearl River (Figure 1); collected by Weihan Shao in September 2019.

Paratypes. 201909112585-8, 20190937564-6, seven ex., 52.8–57.3 mm SL; other data same as holotype.

Diagnosis. A striped *Tachysurus* species. Distinguished from all other striped congeners except *T. bailongensis*, *T. virgatus*, *T. trimaculatus* and *T. furcatus* by the following combination of characters: a deeply (vs. moderately) forked caudal fin, a straight (vs. gently convex) posterior edge of the caudal-fin lobes, presence (vs. absence) of a prominent post-dorsal-fin notch on the dorso-lateral stripe and a uniform and unconstricted (vs. tapering, constricted and irregular) mid-lateral stripe. It further differs from *T. bailongensis* and *T. trimaculatus* by the absence (vs. presence) of a connection between the dorso-lateral stripe and the black blotches on the operculum, from *T. furcatus* in having a narrower caudal peduncle (depth 33.5–41.3% of HL vs. 42.1–45.3%), from *T. virgatus* in having a narrower (vs. broader) whitish-yellow patch along the posterior margin of eyes and absence (vs. presence) of a short whitish-yellow line in the beginning of the mid-lateral stripe.

Distribution and habitat. Currently known from Boluo County, Guangdong Province, belonging to Dong River, a tributary of the Pearl River (Figure 1). Recorded exclusively in flowing streams.

Etymology. The specific epithet is derived from the Greek prefix *pseudo-*, in allusion to the close morphological and phylogenetic affinities of *T. pseudovirgatus* with *T. virgatus*. The onomatopoeic Chinese sound of this species is “Si Zong Wen Ni Chang”.

***Tachysurus bailongensis* Shao & Zhang, sp. nov.**

(Figure 10)

Holotype. FANGC12597, 71.02 mm SL, South China: Guangxi Zhuang Autonomous Region: Fangchenggang City: Naguo Town: Fangcheng River, a coastal river flowing into the Beibu Gulf (Figure 1); collected by Weihan Shao in September 2021.

Paratypes. FANGC12592-6, FANGC12598, seven ex., 61.3–76.8 mm SL; other data same as holotype; DONGX 65305-6, two ex. 42.3–62.2 mm SL, South China: Guangxi Zhuang Autonomous Region: Dongxing City: Jiangpiung Town: Jiangping River, a coastal river flowing into the Beibu Gulf.

Diagnosis. A striped *Tachysurus* species. Distinguished from all other striped congeners except *T. pseudovirgatus*, *T. virgatus*, *T. trimaculatus* and *T. furcatus* by the following combination of characters: a deeply (vs. moderately) forked caudal fin, a straight (vs. gently convex) posterior edges of the caudal-fin lobes, presence (vs. absence) of a prominent post-dorsal-fin notch on the dorso-lateral stripe, and a uniform and unconstricted (vs. tapering, constricted and irregular) mid-lateral stripe. It can be differentiated from *T. trimaculatus*, *T. pseudovirgatus*, *T. virgatus* and *T. furcatus* by a shallower head (depth 53.4–59.1% HL vs. 58.6–71.1% HL) and narrower head (width 59.8–68.5% HL vs. 66.4–76.7% HL), and from *T. pseudovirgatus*, *T. virgatus* and *T. furcatus* by the presence (vs. absence) of a connection between the dorso-lateral stripe and black blotches on the operculum.

Distribution and habitat. Currently known from the Fangcheng River and Jiangping River in Guangxi Zhuang Autonomous Region, China, which are two coastal rivers flowing into the Beibu Gulf basin (Figure 1). Juveniles mainly occur in streams, while adults are restricted to the main channels of rivers.

Etymology. The specific name bailongensis refers to “Bailongwei”, the locality in Fangchenggang City, Guangxi Zhuang Autonomous Region, southern China, where the new species was collected. The name commemorates the historical site of the Bailong Fort, which was constructed by the Qing Dynasty in the late 19th century to defend the Chinese–Vietnamese border against incursions by French colonial forces in Vietnam.

***Tachysurus furcatus* Shao & Zhang, sp. nov.**

(Figure 11)

Holotype. GUANGP63012, 67.40 mm SL, Central Vietnam: Quang Binh Prov.: Nhat Le River, a coastal river flowing into South China Sea (Figure 1); collected by Weihan Shao in June 2025.

Paratypes. GUANGP63001-11, eleven ex., 52.7–73.3 mm SL; other data same as holotype.

Diagnosis. A striped *Tachysurus* species. Distinguished from all other striped congeners except *T. pseudovirgatus*, *T. virgatus*, *T. trimaculatus* and *T. bailongensis* by the following combination of characters: straight (vs. gently convex) posterior edges of the caudal-fin lobes, a deeply (vs. moderately) forked caudal fin, presence (vs. absence) of a prominent post-dorsal-fin notch on the dorso-lateral stripe and a uniform and unconstricted (vs. tapering, constricted and irregular) mid-lateral stripe. It can be differentiated from *T. virgatus*, *T. trimaculatus* and *T. bailongensis* by the origin of the mid-lateral stripe diverging into two or three short black lines (vs. the entire lower portion) connecting to the black blotches on the operculum, from *T. trimaculatus* and *T. bailongensis* by the absence (vs. presence) of a connection between the dorso-lateral stripe and the black blotches on the operculum, and from *T. pseudovirgatus* in having a deeper caudal peduncle (depth 59.5–71.1% vs. 42.1–45.3% HL).

Distribution and habitat. Currently known from the Nhat Le River in Quang Binh Province, and Hương River in Thừa Thiên Huế Province, central Vietnam, which are two coastal rivers flowing into the South China Sea (Figure 1). Recorded exclusively in flowing streams.

Etymology. The specific epithet *furcatus* is derived from Latin, meaning “forked”, referring to the distinctive mid-lateral stripe of this species, which diverges into two or three short black lines to connect to the black blotches on the operculum, forming a fork-like pattern. The species is locally known in Chinese as “Cha Wen Ni Chang”.

**Figure 12:**
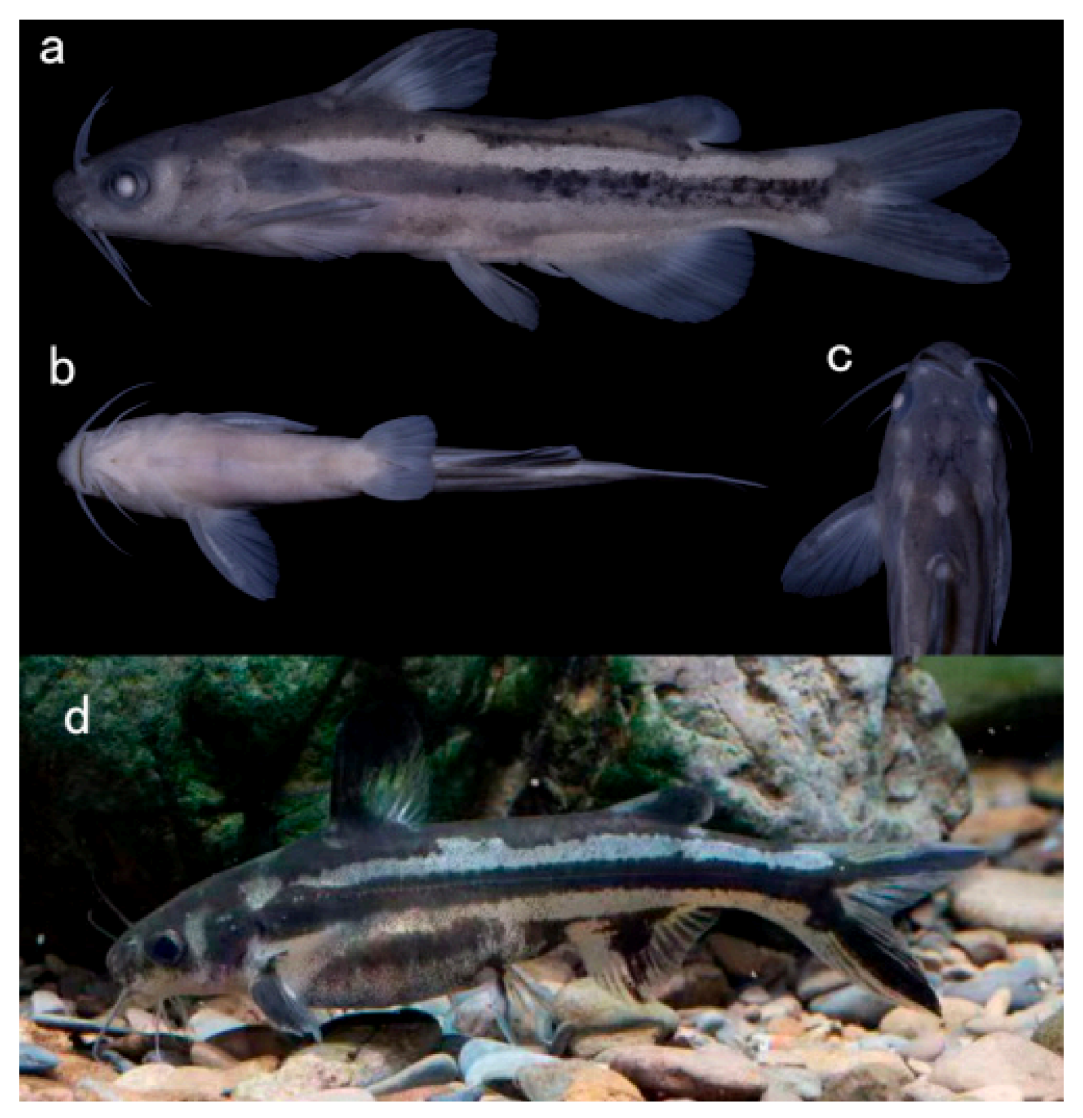
Lateral (**a**), dorsal (**b**) and ventral (**c**) views of *Tachysurus trimaculatus* sp. nov. (**d**) Freshly collected specimen of *T. trimaculatus*.

***Tachysurus trimaculatus* Shao & Zhang, sp. nov.** (Figure 12)

Holotype. PINGHE65311, 69.57 mm SL, Southeast China: Fujian Prov.: Pinghe Town: Jiulong River, a coastal river flowing into the Taiwan Strait (Figure 1); collected by Weihan Shao in June 2019.

Paratypes. PINGHE 65312-7, NANJ9279-9286, fourteen ex., 46.18–71.37 mm SL; other data same as holotype.

Diagnosis. A striped *Tachysurus* species. Distinguished from all other striped congeners by the following combination of characters: a deeply (vs. moderately) forked caudal fin, presence (vs. absence) of a connection between the dorso-lateral stripe and the black blotches on the upper margin of the operculum, and absence (vs. presence) of a connection between the dorso-lateral and mid-lateral stripes anterior to the dorsal fin.

Distribution and habitat. Currently known from the Beixi River, a tributary of the Jiulongjiang River in Fujian Province, Southeast China, which is a coastal river flowing into the Taiwan Strait (Figure 1). Recorded exclusively in flowing streams.

Etymology. The specific epithet is derived from the Latin *tri-*(“three”) and *maculatus* (“spotted”), referring to the distinctive nuchal stripe of this species. In dorsal view, the nuchal white-yellowish stripe, typically continuous in congeners, is uniquely separated into three blotches. The onomatopoeic Chinese sound of this species is “San Ban Ni Chang”.

***Tachysurus platystomus* Shao & Zhang, sp. nov.**

(Figure 13)

Holotype. HAIF01, 88.84 mm SL, South China: Guangdong Prov.: Haifeng County: Huang River, a coastal river flowing into the South China Sea (Figure 1); collected by Weihan Shao in July 2020.

Paratypes. HAIF02-8, seven ex., 34.15–137.54 mm SL; other data same as holotype.

Diagnosis. A striped *Tachysurus* species. Distinguished from all other striped congeners except *T. heterofasciatus* by the moderately (vs. deeply) forked caudal fin. It can be further differentiated from *T. heterofasciatus* in having a shorter dorsal spine (length 15.7–20.3% SL vs. 19.1–24.4%), a wider mouth (width 12.3–14.7% SL vs. 8.9–11.7%) and a mid-lateral stripe with slight (vs. distinct) constriction at the posterior end of the dorsal and adipose fins.

Distribution and habitat. Currently known from the Huangjiang River, in Haifeng County, Guangdong Province, South China, which is a coastal river flowing into the South China Sea (Figure 1). Recorded exclusively in flowing streams.

Etymology. The specific name *platystomus* is derived from the Greek *platy-*(“broad”) and *-stomus* (“mouth”), referring to the broad mouth of this species, which is wider than that of all other striped *Tachysurus* species. The onomatopoeic Chinese sound of this species is “Kuankou Ni Chang”.

***Tachysurus heterofasciatus* Shao & Zhang, sp. nov.**

(Figure 14)

Holotype. YANGC47901, 102.53 mm SL, South China: Guangdong Prov.: Yangchun City: E’Huangzhang Natural Reserve: Moyangjiang River, a coastal river flowing into the South China Sea (Figure 1); collected by Ke Zheng in July 2024.

Paratypes. YANGC47902-11, ten ex., 57.79–156.92 mm SL; other data same as holotype.

**Diagnosis**. A striped *Tachysurus* species that can be easily distinguished from non-striped congeners by the coloration consisting of two black, longitudinal and continuous stripes extending from the head to the caudal peduncle. Distinguished from all other striped congeners except *T. platecephalus* by the moderately (vs. deeply) forked caudal fin. It can be further differentiated from *T. platecephalus* in having a longer dorsal spine (length 19.1–24.4% vs. 15.7–20.3% SL), a narrower mouth (width 8.9–11.7% SL vs. 12.3–14.7%) and a mid-lateral stripe distinctly (vs. slightly) constricted at the posterior end of the dorsal and adipose fins.

Distribution and habitat. Currently known from the E’ Huangzhang Natural Reserve, Moyang River, in Yangchun City, Guangdong Province, South China, which is a coastal river flowing into the South China Sea (Figure 1). Recorded exclusively in flowing streams.

**Etymology.** The specific epithet is derived from the Greek *hetero-*(“different”) and Latin *-fasciatus* (“banded”), referring to the species’ unique mid-lateral stripe, which is of uniform width but interrupted by two pronounced constrictions, in contrast to other striped species, where the mid-lateral stripe is either uniformly wide without constrictions or gradually narrows with constrictions. The onomatopoeic Chinese sound of this species is “Yi Wen Ni Chang”.

***Tachysurus analimaculatus* Shao & Zhang, sp. nov.**

(Figure 15)

Holotype. GUANGZ37486, 72.40 mm SL, China: Guangdong Prov.: Guangzhou City, Zhongluotan Town, Beijiang River, a tributary of the Pearl River (Figure 1); collected by Weihan Shao in March 2020.

Paratypes. GUANGZ37481-5, five ex., 56.68–87.19 mm SL, other data same as holotype; GUIGANG65213-7, five ex., 41.00–51.91 mm SL, China: Guangxi Zhuang Autonomous Region: Guigang City, Xijiang River, a tributary of the Pearl River.

Diagnosis. A striped *Tachysurus* species. Distinguished from all other striped congeners except *T. qiongzhouensis* and *T. sinovietnamensis* by the following combination of characters: a deeply (vs. moderately) forked caudal fin, presence (vs. absence) of a prominent post-dorsal-fin notch on the dorso-lateral stripe and a constricted, irregular and tapering (vs. unconstricted and uniformly wide) mid-lateral stripe. It can be differentiated from *T. qiongzhouensis* and *T. sinovietnamensis* in having a more slender body (depth at anus 13.8–19.2% HL vs. 19.1–24.5%). It further differs from *T. qiongzhouensis* by having a shorter nasal barbel (30.6–44.8% HL vs. 44.1–69.8%), and from *T. sinovietnamensis* by having a more slender caudal peduncle (depth 15.2–17.6% pre-anal length vs. 12.6–15.7%).

Distribution and habitat. Currently known from the Pearl River, South China (Figure 1). Inhabiting exclusively main channels of rivers.

Etymology. The specific epithet “*analimaculatus*” is a compound adjective derived from the Latin terms *analis*- (pertaining to the anal fin) and *maculatus-* (spotted or marked with patches). This name refers to the prominent black blotch on the anal fin. The onomatopoeic Chinese sound of this species is “Tun Ban Ni Chang”.

***Tachysurus qiongzhouensis* Shao, Cai & Zhang, sp. nov.**

(Figure 16)

Holotype. HAIK37477, 88.29 mm SL, China: Hainan Prov.: Haikou City, Xinpo Town, Nandu River, a coastal river of Hainan Island (Figure 1); collected by Xingwei Cai in November 2019.

Paratypes. HAIK37472-6, HAIK200304-8, ten ex., 56.56–98.28 mm SL, other data same as holotype; YANGC57807-10, 57813-14, five ex., 60.74–69.67 mm SL, China: Guangdong Prov.: Yangchun City, Heshui Town, Moyang River, a coastal river of south China.

Diagnosis. A striped *Tachysurus* species. Distinguished from all other striped congeners except *T. analimaculatus* and *T. sinovietnamensis* by the following combination of characters: a deeply (vs. moderately) forked caudal fin, presence (vs. absence) of a prominent post-dorsal-fin notch on the dorso-lateral stripe and a constricted, irregular and tapering (vs. unconstricted and uniformly wide) mid-lateral stripe. It can be differentiated from *T. analimaculatus* in having a deeper body (depth at anus 19.1–24.5% vs. 13.8–19.2% HL). *Tachysurus qiongzhouensis* exhibits overall morphological similarity to *T. sinovietnamensis* but can be distinguished by the ratios of SL/HL to maxillary barbel length (71.28–89.23% vs. 45.23–56.34%) and SL/HL to nasal barbel length (34.21–36.21% vs. 67.21–78.21%).

Distribution and habitat. Currently known from the Nandu and Changhua Rivers on Hainan Island and the Moyang River in Guangdong Province, South China (Figure 1). Inhabiting exclusively main channels of rivers.

Etymology. The specific epithet is derived from Qiongzhou, the historical designation for Hainan Island and the primary distributional range of this species. This species also inhabits the Moyang River in Guangdong Province, a region situated across the Qiongzhou Strait from Hainan Island. This epithet emphasizes the geographical affinity linking the populations of these two adjacent regions. The onomatopoeic Chinese sound of this species is “Qiong Zhou Ni Chang”.

***Tachysurus sinovietnamensis* Shao, Nguyen & Zhang, sp. nov.**

(Figure 17)

Holotype. YUANY13. 71.90 mm SL, China: Yunnan Prov.: Yuanyang County, Red River (Figure 1); collected by Hongfu Yang in November 2024.

Paratypes. YUANY11-12, 15–16, four ex., 50.01–69.91 mm SL, other data same as holotype; BN202501-4, four ex., 52.74–65.27 mm SL, Vietnam: Bắc Ninh Prov.: Bắc Ninh City, Cầu River, a tributary of Red River.

Diagnosis. A striped *Tachysurus* species. Distinguished from all other striped congeners except *T. analimaculatus* and *T. sinovietnamensis* by the following combination of characters: a deeply (vs. moderately) forked caudal fin, presence (vs. absence) of a prominent post-dorsal-fin notch on the dorso-lateral stripe and a constricted, irregular and tapering (vs. unconstricted and uniformly wide) mid-lateral stripe. It can be differentiated from *T. analimaculatus* in having a deeper body (depth at anus 19.1–24.5% vs. 13.8–19.2% HL). *Tachysurus sinovietnamensis* exhibits overall morphological similarity to *T. qiongzhouensis* but can be distinguished by the ratios of SL/HL to maxillary barbel length (71.28–89.23% vs. 45.23–56.34%) and SL/HL to nasal barbel length (34.21–36.21% vs. 67.21–78.21%).

Distribution and habitat. Currently known from the Red River in Southwest China and Northern Vietnam as well as coastal rivers in northern central Vietnam including the Gianh and Lam Rivers in Quang Binh and Ha Thin Provinces, respectively (Figure 1). Inhabiting exclusively main channels of rivers.

Etymology. The specific epithet refers to the known distribution of this species across China (*Sino-*) and Vietnam (*-vietnamensis*).

### 5.2. Identification Key to Species in the T. virgatus Group

1a. Caudal fin deeply forked21b. Caudal fin moderately forked102a. Caudal fin with broad lobes bearing two irregular black patches
*T. spilotus*
2b. Caudal fin with narrow lobes bearing two black short stripes33a. Mid-lateral stripe constricted, irregular and tapering43b. Mid-lateral stripe unconstricted and uniformly wide64a. A slender body depth at anus 13.8–19.2% SL
*T. analimaculatus*
4b. A deep body depth at anus 19.1–24.5% SL55a. Ratio of SL/HL to maxillary barbel length 25.28–51.1%
*T. qiongzhouensis*
5b. Ratio of SL/HL to maxillary barbel length 46.86–88.76% %
*T. sinovietnamensis*
6a. Dorso-lateral stripe connected with blotches on operculum76b. Dorso-lateral stripe well separated from opercular blotches87a. Dorso-lateral stripe not connected or slightly connected with mid lateral stripe
*T. trimaculatus*
7b. Dorso-lateral stripe broadly connected with mid lateral stripe
*T. bailongensis*
8a. A short yellow line interrupting origin of mid-lateral stripe
*T. virgatus*
8b. Uninterrupted mid-lateral stripe origin99a. A deep caudal peduncle 42.1–45.3% HL
*T. furcatus*
9b. A narrow caudal peduncle 33.5–41.3% HL
*T. pseudovirgatus*
10a. Two black longitudinal stripes extending from head to caudal1110b. Three broad vertical brown blotches along body
*T. kyphus*
11a. Short dorsal spine 15.7–20.3% SL and a wide mouth 12.3–14.7% SL
*T. platystomus*
11b. Long dorsal spine 19.1–24.4% SL and a narrow mouth 8.9–11.7% SL
*T. heterofasciatus*


## 6. Conclusions

This study employs an integrative taxonomic framework combining morphological and molecular evidence to reveal hidden species diversity within the *Tachysurus virgatus* s.l. along the northern margin of the East Asian tropics. Our results uncover remarkable cryptic diversity: true *T. virgatus* is restricted to the Wanquan River on Hainan Island, whereas nine additional lineages are described as new species. Together with *T. kyphus* and *T. spilotus*, these taxa constitute a well-supported monophyletic group. The pronounced decoupling between molecular divergence and morphological differentiation highlights the prevalence of a “grey zone” of speciation within this lineage. Guided by the Unified Species Concept and supported by multiple independent lines of evidence, including habitat specialization and geographic distribution, this study establishes robust and defensible species delineation.

## Figures and Tables

**Figure 10:**
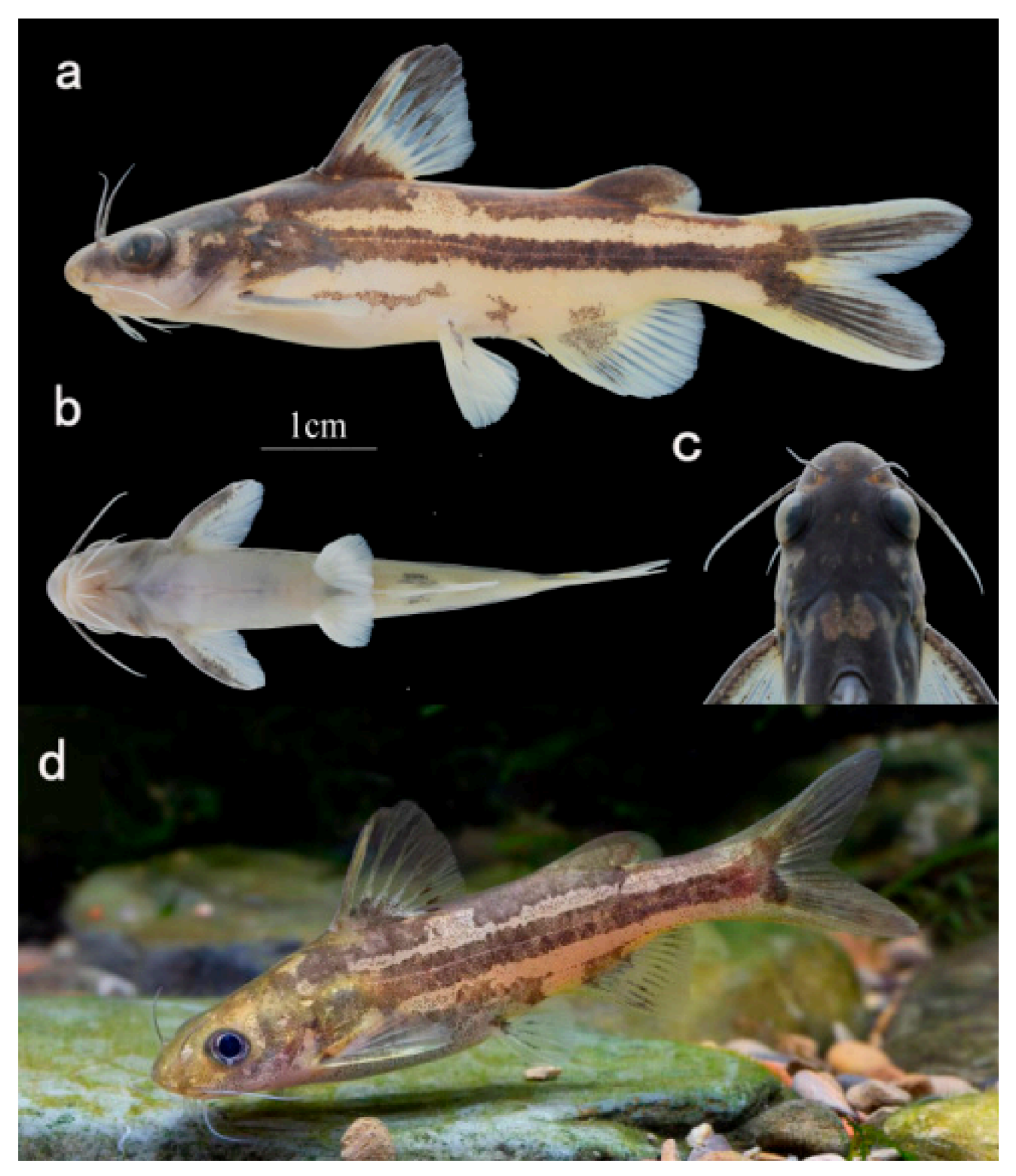
Lateral (**a**), dorsal (**b**) and ventral (**c**) views of *Tachysurus bailongensis* sp. nov. (**d**) Freshly collected specimen of *T. bailongensis*.

**Figure 11:**
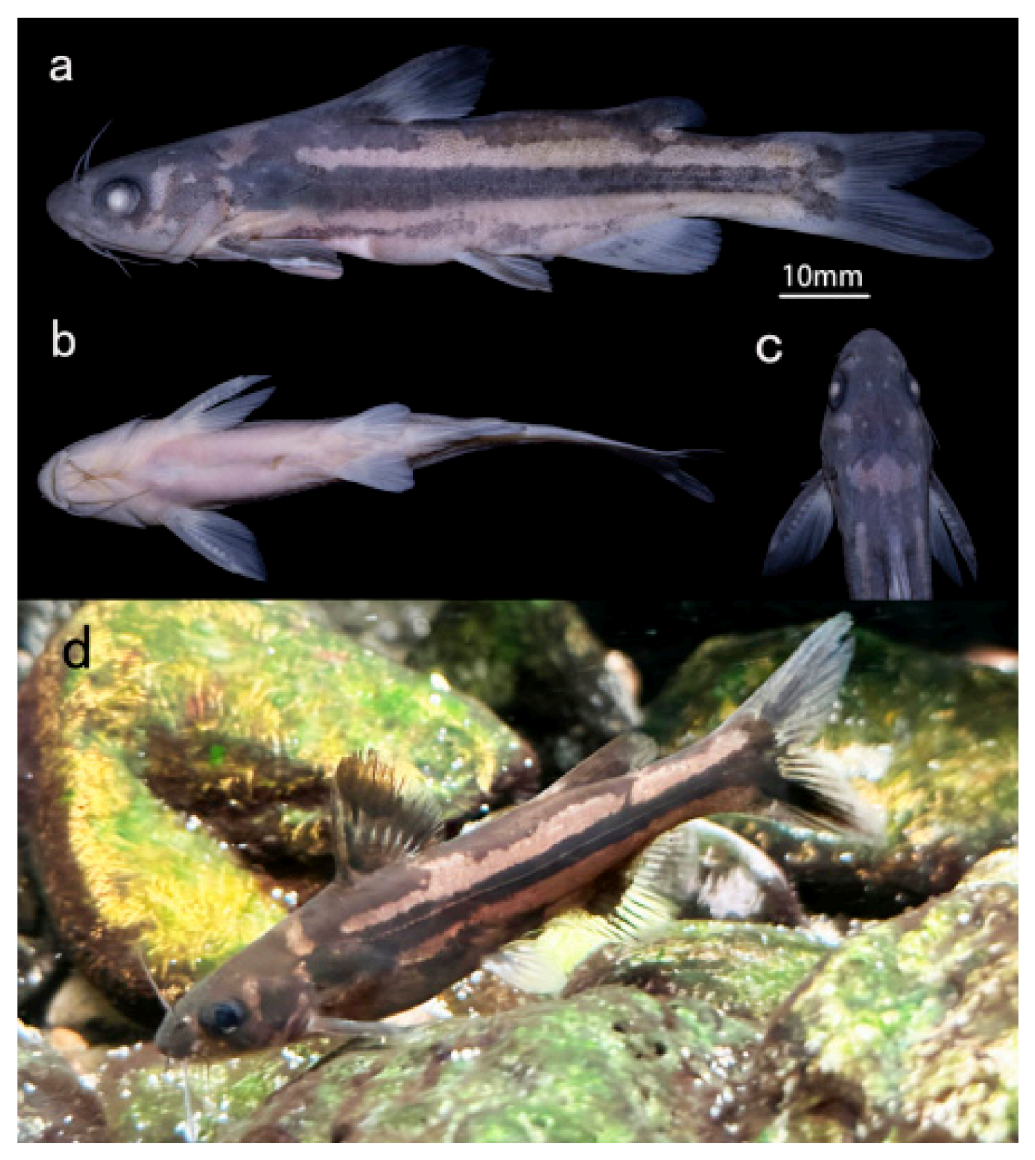
Lateral (**a**), dorsal (**b**) and ventral (**c**) views of *Tachysurus furcatus* sp. nov. (**d**) Freshly collected specimen of *T. furcatus*.

**Figure 13:**
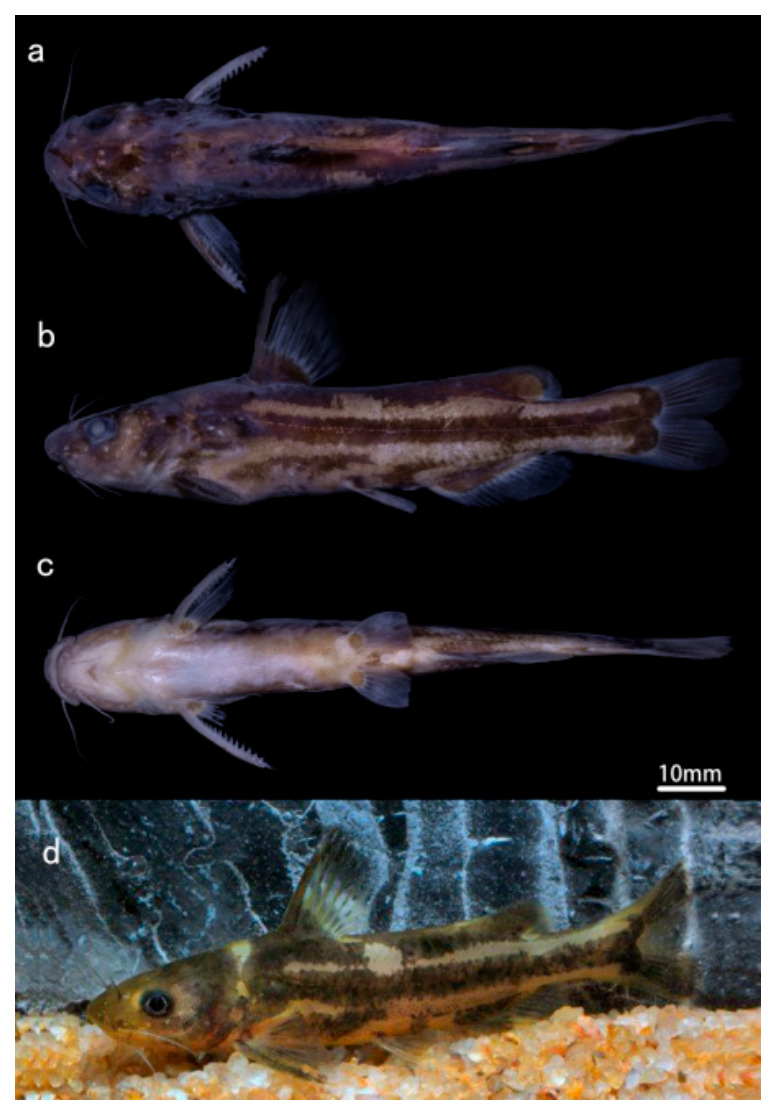
Lateral (**a**), dorsal (**b**) and ventral (**c**) views of *Tachysurus platystomus* sp. nov. (**d**) Freshly collected specimen of *T. platystomus*.

**Figure 14:**
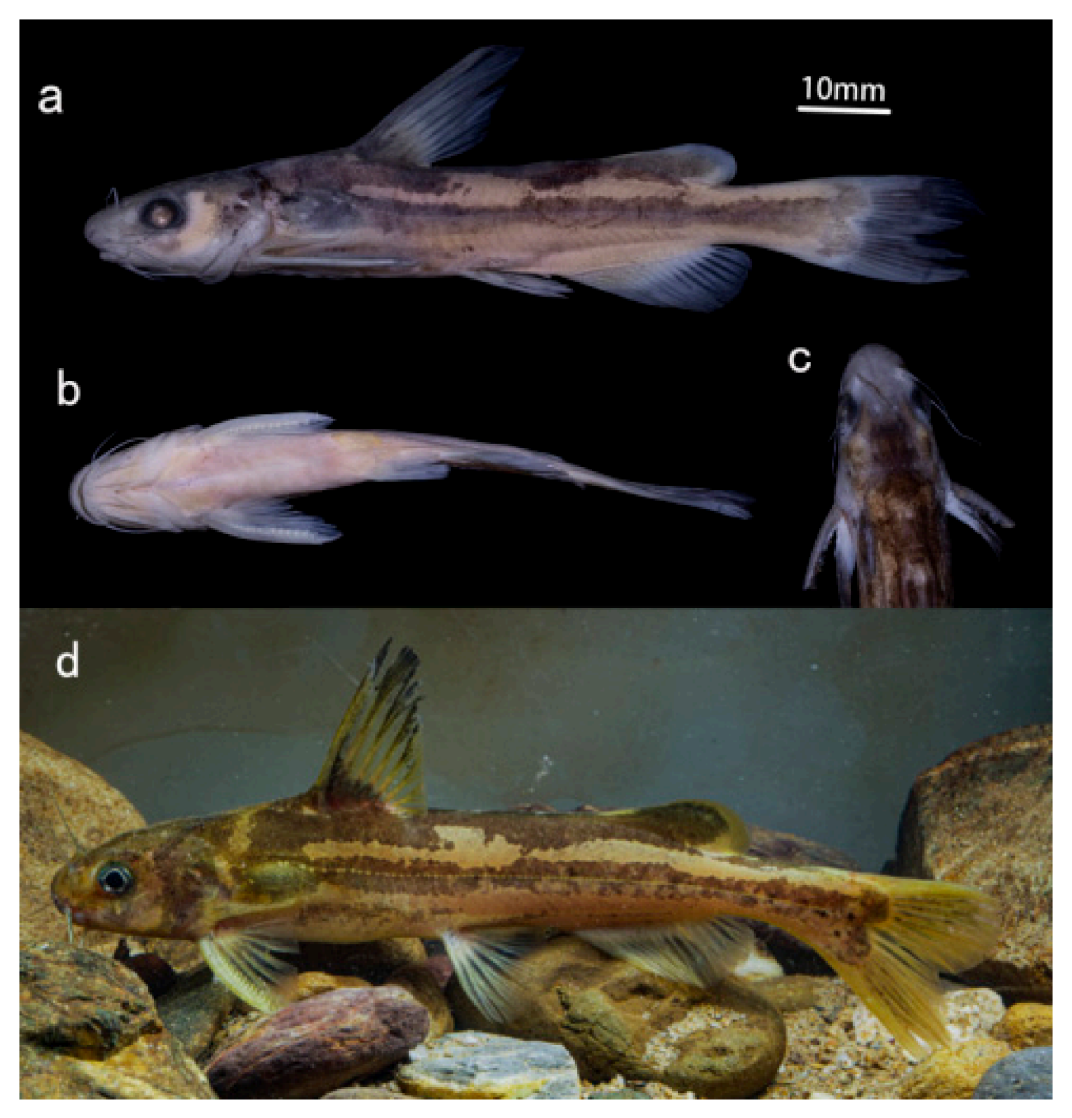
Lateral (**a**), dorsal (**b**) and ventral (**c**) views of *Tachysurus heterofasciatus* sp. nov. (**d**) Freshly collected specimen of *T. heterofasciatus*.

**Figure 15:**
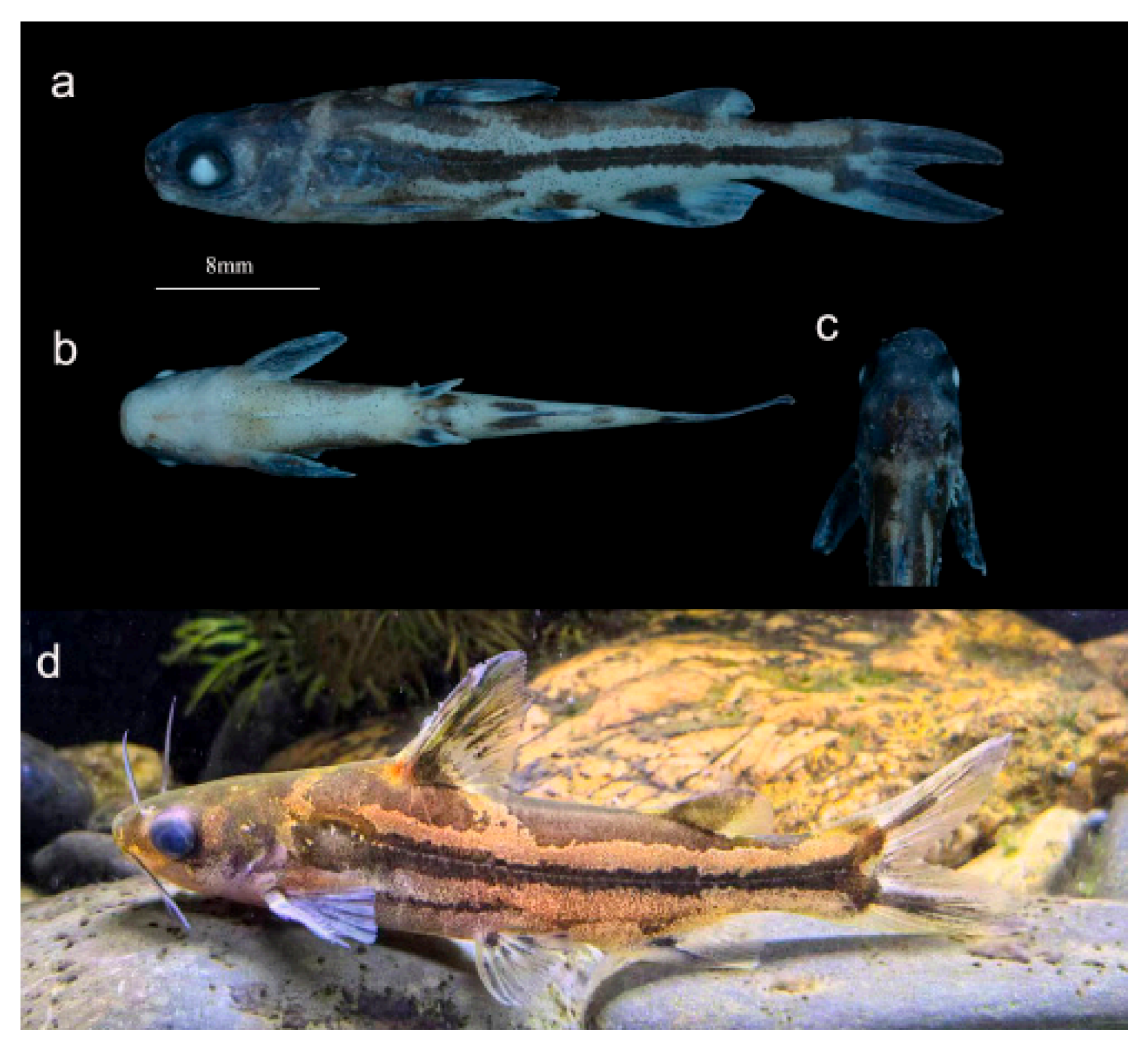
Lateral (**a**), dorsal (**b**) and ventral (**c**) views of *Tachysurus analimaculatus* sp. nov. (**d**) Freshly collected specimen of *T. analimaculatus*.

**Figure 16:**
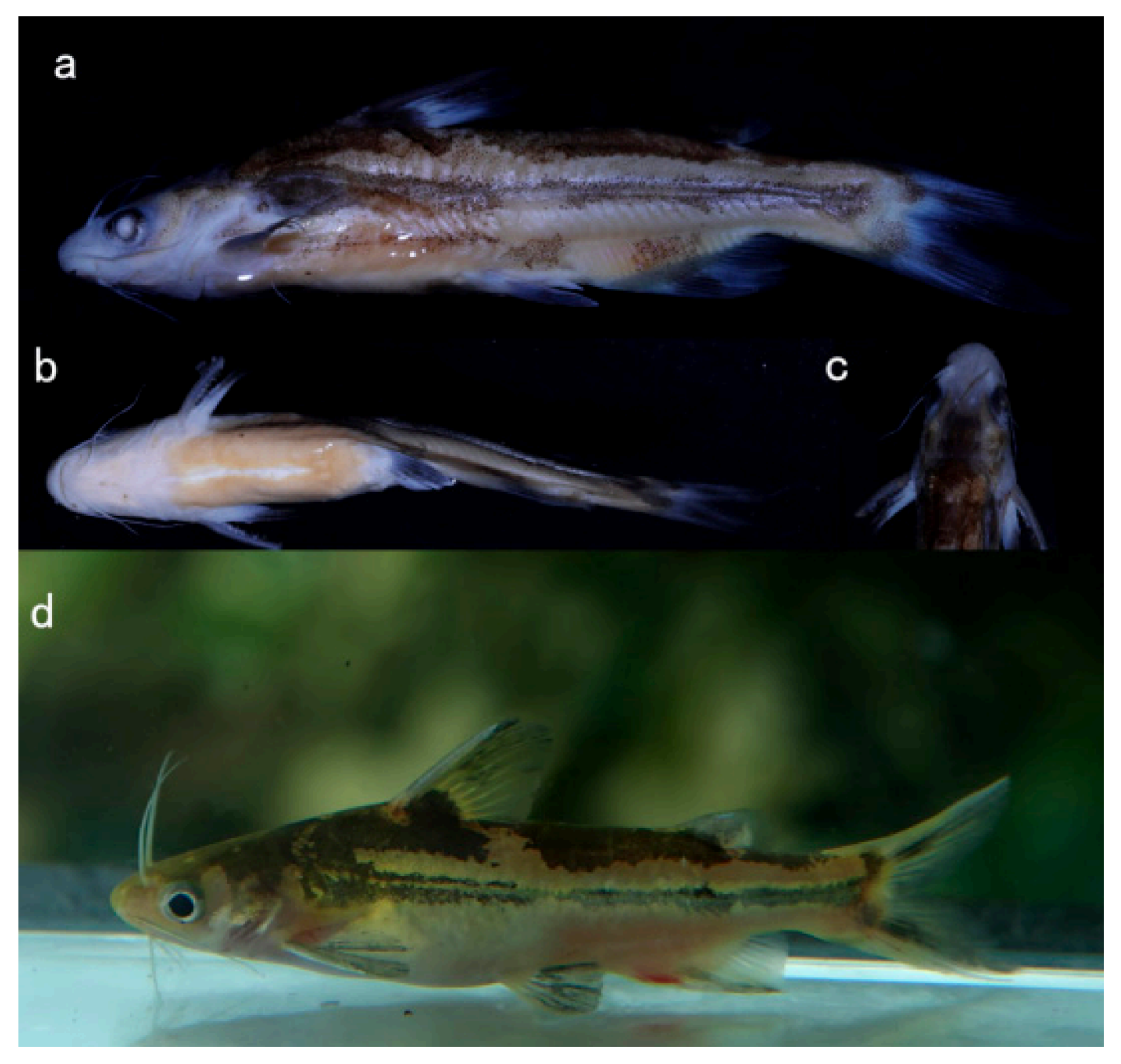
Lateral (**a**), dorsal (**b**) and ventral (**c**) views of *Tachysurus qiongzhouensis* sp. nov. (**d**) Freshly collected specimen of *T. qiongzhouensis*.

**Figure 17:**
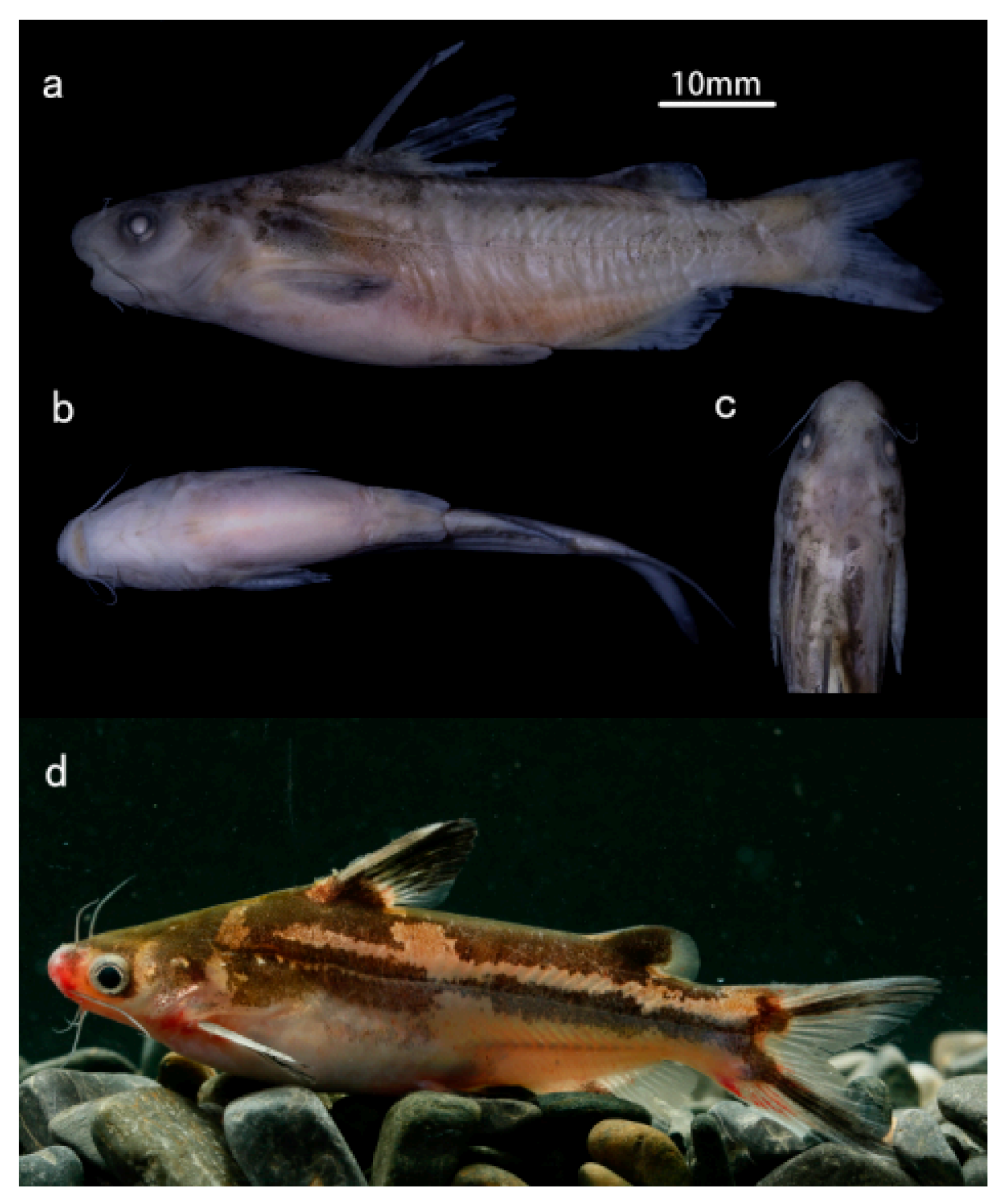
Lateral (**a**), dorsal (**b**) and ventral (**c**) views of *Tachysurus sinovietnamensis* sp. nov. (**d**) Freshly collected specimen of *T. sinovietnamensis*.

**Table 1:** Species used in this study for morphological analysis.

Taxon	Locality/Province/Country	Drainage	Voucher Number
*T. virgatus*	1. Qionghai, Hainan, China	Wanquan River	QIONGH65476-503
	2. Qiongzhong, Hainan, China	Wanquan River	QIONGZ30102-8
*T. kyphus*	3. Hekou, Yunnan, China	Red River	HEK65708-13
*T. qiongzhouensis*	4. Haikou, Hainan, China	Nandu River	HAIK37472-7, HAIK200301-5
	5. Ledong, Hainan, China	Changhua River	LED 253701
	6. Yangchun, Guangdong, China	Moyang River	YANGC57807-13
*T. analimaculatus*	7. Guangzhou, Guangdong, China	Pearl River	GUANGZ37480-4
	8. Foshan, Guangdong, China	Pearl River	FOS62101-7
	9. Huizhou, Guangdong, China	Pearl River	HUIZ37487
	10. Guigang, Guangxi, China	Pearl River	GUIG65213-7
*T. sinovietnamensis*	11. Quang Binh, Vietnam	Gianh River	GUANGP17990-3
	12. Ha Giang, Vietnam	Red River	HEJ36168
	13. Ha Tinh, Vietnam	Lam River	HEJING18141-2
	14. Yuanyang, Yunnan, China	Red River	YUANY18011-5
	22. Bac Ninh, Vietnam	Red River	BEIN251001
*T. pseudovirgatus*	15. Boluo, Guangdong, China	Dong River (tributary of Pearl River)	BOL12581-8
*T. bailongensis*	16. Fangcheng, Guangxi, China	Fangcheng River	FANGC12590-9
	17. Dongxing, Guangxi, China	Beilun River	DONGX65305, 8
*T. trimaculatus*	18. Pinghe, Fujian, China	Jiulong River	PINGHE65311-8, NANJ9279-86
*T. furcatus*	19. Quang Binh, Vietnam	Nhat Le River	GUANGP63001-15
*T. platystomus*	20. Haifeng, Guangdong, China	Huangjiang River	SHANW37470-7
*T. heterofasciatus*	21. Yangchun, Guangdong, China	Moyang River	YANGJ47901-9
*T. spilotus*	23. Da Nang, Vietnam	Tuy Loan River	XIANG251001-2

**Table 2:** Taxa examined in this study with Genebank accession numbers.

Taxon	Voucher Number	Genebank Accession Number		Source
		*COI*	*cyt-B*	
*T. virgatus*	QIONGH65454	PX290058	PX311038	This study
*M.*	QIONGH65455	PX290059	PX311039	This study
	QIONGH65456	PX290060	PX311040	This study
*T. pseudovirgatus*	BOL12583	PX290029	PX311009	This study
	BOL12585	PX290030	PX311010	This study
	BOL12586	PX290031	PX311011	This study
	BOL12588	PX290032	PX311012	This study
*T. furcatus*	GUANGP63003	PX290039	PX311019	This study
	GUANGP63007	PX290040	PX311020	This study
	GUANGP63009	PX290041	PX311021	This study
	GUANGP63010	PX290044	PX311022	This study
*T. bailongensis*	FANGC12593	PX290033	PX311013	This study
	FANGC12597	PX290034	PX311014	This study
	DONGX65305	PX290035	PX311015	This study
	DONGX65308	PX290036	PX311016	This study
*T. platystomus*	SHANW10056	PX290061	PX311041	This study
	SHANW37470	PX290062	PX311042	This study
	SHANW37472	PX290063	PX311043	This study
	SHANW37473	PX290064	PX311044	This study
*T. heterofasciatus*	YANGJ47903	PX290066	PX311046	This study
	YANGJ47904	PX290067	PX311047	This study
	YANGJ47907	PX290068	PX311048	This study
*T. trimaculatus*	NANJ49279	PX290055	PX311035	This study
	NANJ49282	PX290056	PX311036	This study
	PINGHE65312	PX290057	PX311037	This study
*T. analimaculatus*	GUANGZ37480	PX290043	PX311023	This study
	GUANGZ37483	PX290044	PX311024	This study
	HUIZ37487	PX290054	PX311034	This study
	GUIG65217	PX290045	PX311025	This study
*T. qiongzhouensis*	HAIK37475	PX290046	PX311026	This study
	HAIK65471	PX290047	PX311027	This study
	HAIK65472	PX290048	PX311028	This study
	LED37478	PX290049	PX311029	This study
	YANGC57811	PX290065	PX311045	This study
*T. kyphus*	HEKOU65328	PX290052	PX311032	This study
	HEKOU65329	PX290053	PX311033	This study
*T. sinovietnamensis*	GUANGP17989	PX290037	PX311017	This study
	YUANY98701	PX290038	PX311018	This study
	HEJ36168	PX290050	PX311030	This study
	HEJING18140	PX290051	PX311031	This study
*T.spilotus*	XIANG251001	PZ639086	PZ660332	This study
	XIANG251002	PZ639087	PZ660332	This study
In group				
*T.tenuis*		MZ636450	MZ636464	[30]
*T. albomarginatus*		MT363236	MT379998	[30]
*T. crassilabris*		PP268890	PP266665	[30]
*T. vachelli*		MZ636442	MZ636437	[30]
*T. nitidus*		MZ636440	MZ636435	[30]
*T. gracilis*		MZ636452	MZ636466	[30]
*T. wuyueensis*		PP266645	PP266644	NCBI
*T.truncatus*		MZ636441	MZ636436	[30]
*T. taeniatus*		MZ636457	MZ636471	[30]
*T. intermedius*		MZ636458	MZ636472	[30]
*T. sinensis*		MZ636439	MZ636434	[30]
*T. eupogon*		MZ636459	MZ636473	[30]
*T. tokiensis*		AB054127	AB054127	NCBI
*T. argentivittatus*		PQ788031	PQ773777	[10]
*T. mica*		PQ788118	PQ773861	[10]
Out group				
*T. trilineatus*		MZ636461	MZ636475	[30]

**Table 3:** Uncorrected p-distances for species within the *T. virgatus* group based on the *cyt-B* gene.

		1	2	3	4	5	6	7	8	9	10	11
Clade I	1. *T. pseudovirgatus*											
	2. *T. virgatus*	2.7										
	3. *T. bailongensis*	2.8	2.9									
	4. *T. furcatus*	3.8	4.0	1.5								
	5. *T. platystomus*	7.4	6.4	7.6	8.2							
	6. *T. heterofasciatus*	7.5	6.4	7.8	8.5	2.1						
	7. *T. trimaculatus*	8.1	8.0	7.7	8.4	9.2	9.0					
Clade II	8. *T. analimaculatus*	13.2	12.1	13.2	13.9	13.3	13.2	13.4				
	9. *T. qiongzhouensis*	13.0	12.3	13.1	13.6	12.9	13.1	13.5	1.9			
	10. *T. kyphus*	13.5	12.9	13.1	13.5	13.5	13.4	13.2	3.5	3.6		
	11. *T. sinovietnamensis*	11.9	11.6	14.7	15.6	12.8	14.5	14.7	3.9	3.7	2.6	
	12. *T. spilotus*	12.1	11.8	13.9	15.7	12.5	13.8	15.1	3.8	3.1	2.8	0.9

**Table 4:** The meristic characters of species within the *T. virgatus* group.

	Dorsal-Fin Rays	Anal-Fin Rays	Pectoral-Fin Rays	Pelvic-Fin Rays	Total Vertebrae
*T. virgatus*	7	12–15	7–8	6	30–31
*T. kyphus*	7	14–16	7	6	31–32
*T. spilotus*	6	14–17	7–8	6	31–34
*T. qiongzhouensis*	7	15–17	7	6	31–32
*T. analimaculatus*	7	15–16	7	6	32–33
*T. sinovietnamensis*	7	12–16	7–8	6	31–34
*T. pseudovirgatus*	7	14–15	7	6	30–32
*T. trimaculatus*	6–7	13–14	7	6	30–31
*T. bailongensis*	7	12–14	7	6	30–33
*T. furcatus*	7	13–14	7–8	6	30–32
*T. platystomus*	7	15–17	7	6	35–36
*T. heterofasciatus*	7	15–17	7	6	35–36

## Data Availability

The original molecular data presented in this study are openly available at https://www.ncbi.nlm.nih.gov/ (assessed on 1 Dcecmber 2026).

## Supplementary Materials

The following supporting information can be downloaded at https://www.mdpi.com/article/10.3390/ani16152422/s1, File S1: Detailed species description of novel species described in this study; File S2: Morphomertric data for species within *T. virgatus* s.l. (Table S1. Morphomertric data for *T. pesudovirgatus* sp. nov. and *T. virgatus* s. str.; Table S2. Morphomertric data for *T. trimaculatus* sp. nov., *T. bailongensis* sp. nov. and *T. furcatus* sp. nov.; Table S3. Morphomertric data for *T. platycephalus* sp. nov. and *T. heterofasciatus* sp. nov.; Table S4. Morphomertric data for *T. analimaculatus* sp. nov., *T. qiongzhouensis* sp. nov. and *T. sinovietnamensis* sp. nov.); Figure S1: Morphometric analyses of *T. virgatus*, *T. pseudovirgatus* and *T. furcatus*; Figure S2: Lacteal view of (a) *T. platystomus* and (b) *T. heterofasciatus*.
