# Supplementary material for: Beyond Morphological Conservatism: Integrative Taxonomy Unveils Remarkable Cryptic Diversity in the Tachysurus virgatus (Oshima 1926) Group (Siluriformes: Bagridae) Endemic to the Northern Margin of East Asian Tropics
Source: Animals (Basel). 2026 Aug 5;16(15):2422. doi: 10.3390/ani16152422 (PMC13466063; doi:10.3390/ani16152422)
Supplement: Supplementary file 1 [file animals-16-02422-s001.zip › Supplementary File S1.pdf]

***Tachysurus pseudovirgatus* sp. nov.**

(Figure 9)

Holotype. 201909112589, 59.2 mm SL, South China: Guangdong Prov.: Boluo County: a stream in Dong River, the tributary of the Pearl River (Figure 1); collected by Weihan Shao in Sept., 2019.

Paratypes. 201909112585−8, 20190937564−6, seven ex., 52.8−57.3 mm SL; other data same as holotype.

Diagnosis. A striped *Tachysurus* species can be easily distinguished from non-striped congeners by the coloration consisting of two black, longitudinal and continuous stripes extending from the head to the caudal peduncle. Distinguished from all other striped congeners except *T. bailongensis*, *T. virgatus*, *T. trimaculatus* and *T. furcatus* by the following combination of characters: a deeply (vs. moderately) forked caudal fin, a straight (vs. gently convex) posterior edge of the caudal-fin lobes, presence (vs. absence) of a prominent post-dorsal-fin notch on the dorso-lateral stripe and a uniform and unconstricted (vs. tapping, constricted and irregular) mid-lateral stripe. It further differs from *T. bailongensis* and *T. trimaculatus* by the absence (vs. presence) of a connection between the dorso-lateral stripe and the black blotches on the operculum, from *T. furcatus* in having a narrower caudal peduncle (depth 33.5−41.3% of HL vs. 42.1−45.3%), from *T. virgatus* in having a narrower (vs. broader) whitish-yellow patch along the posterior margin of eyes and absence (vs. presence) of a short whitish-yellow line in the beginning of the mid-lateral stripe.

Description. See Figure 9 for general body appearance. Morphometric measurements taken from the holotype and paratypes summarized in Table S1.

Body elongated, anteriorly cylindrical and posteriorly slightly compressed. Dorsal profile rising evenly and steeply from tip of snout to origin of dorsal fin, sloping gently ventrally from origin of dorsal fin to end of caudal peduncle. Ventral surface of head flattened; Ventral profile slightly convex to anal-fin base, then sloping slightly dorsally to end of caudal peduncle. Lateral line complete, straight and mid-lateral in position. Vertebrae 5+31, (30, 32). Head depressed, broad, and covered with thin skin. Supraoccipital process slender, with evenly converging sides and

pointed tip, separated from nuchal plate by broad interspace. Snout slightly pointed in dorsal view and obtuse or blunt in lateral view, longer than eye diameter. Interorbital space moderately wide and slightly flattish. Eyes moderately large, elliptical, covered with thick membrane, and anterolateral in head, visible when viewed dorsally, but not ventrally.

Mouth subterminal, transverse. Upper jaw anteriorly protruded, longer than lower jaw in length. Teeth villiform, in irregular rows on all toothbearing surfaces. Premaxillary tooth plates broad, of equal width throughout. Dentary tooth plates arched, broadest at symphysis and narrowing laterally, of same width at symphysis as premaxillary tooth plates. Vomerine tooth plate unpaired, continuous across midline, slightly curved anteriorly and much narrower than premaxillary plate. Gill opening wide, extending from post-temporal region to beyond isthmus.

Barbels in four pairs; nasal barbels small, thread-like, slightly extending posterior margin of eye, and maxillary barbels slender, not extending or slightly exceeding base of petoral-fin. Mandibular barbels in two pairs, thick, short; inner barbels positioned in transverse row at level of posterior naris, slightly extending beyond posterior of eye, and outer barbels rooted posterolateral to inner mandibular barbel, significantly extending to posterior margin of eye.

Dorsal fin with a spine and 7 soft branched rays. Dorsal-fin origin close to pectoral-fin insertion than pelvic-fin insertion, equidistant to anal-fin origin and snout tip. Dorsal fin spine slender, with smooth posterior edge, equal to or slightly longer than pectoral-fin spine. First dorsal-fin soft ray longest, surpassing tip of last ray. Distal margin of dorsal-fin rays nearly straight. Nuchal plate triangular, with anterior pointed tip.

Adipose fin inserted slightly behind anal fin origin, with convex distal margin along entire length and deeply incised posterior part to form rounded apex. Adipose fin base short, significantly shorter than anal fin base length.

Pectoral fin with a spine and 7 soft branched rays, inserted slightly anterior or at level of posteriormost point of opercle, not reaching halfway to pelvic-fin insertion. Pectoral fin spine very stout, sharply pointed at tip, with a smooth anterior margin and

11 (10, 12 mean 11.4) strong serrations along posterior margin. Cleithral process triangular with a sharp pointed tip, extending to half of pectoral-fin spine length.

Pelvic fin with one unbranched and five branched soft rays, inserted closer to posterior end of anal fin base than to tip of snout, closer to anterior end of anal fin base than to depressed tip of dorsal fin. Tip of depressed pelvic fin reaching or slightly extending beyond anal fin origin. Pelvic fin distal margin convex. Anus and urogenital opening nearer to anal fin origin than to posterior end of pelvic fin base. Males with a conical genital papilla not reaching base of first anal fin soft ray.

Anal fin long, with 14 (15) branched rays; inserted anterior to adipose fin origin. Anal fin origin nearer to caudal-fin base than to tip of snout. Distal margin of anal fin convex; anterior rays shortest.

Caudal fin with 8+10 (11) principal rays, deeply forked, with middle rays shorter than two thirds of longest rays; both lobes narrow and straight, with upper lobe equidistant to lower lobe; procurrent rays slightly extending from anterior to fin base. Shallowest point of caudal peduncle behind posterior end of anal fin base.

Coloration. Body gray, with two longitudinal black bands along sides. Mid-lateral stripe running along lateral line, black, continuous, and uniform; bifurcating at caudal base into two black narrow branches extending along the upper and lower caudal-fin lobes to their tips. Each branch occupies three fin rays. Dorsum with a broad black stripe, connecting with mid-lateral stripe anterior to dorsal fin. Three irregular grayish blotches present on ventral quarter of body, arranged in series from post-temporal region to caudal-fin base. Nape with a broad and yellow-whitish band, extending downwards from head to middle of eye. Mid-point of mid-lateral stripe extends as a short black branch joining with black blotches on operculum. A vertical yellow-whitish band present posterior to the eye, extending from the dorsal margin of the eye to the ventral surface. Operculum with a short, narrow whitish-yellow luminescent patch along its upper margin.

Adipose fin cream, with a scatted brown melanophores forming a brown band at the anterior two thirds of fin and a slightly hyaline posterodorsal margin. Dorsal fin brown on distal half, rest of fin hyaline. Pectoral, pelvic and anal fins hyaline with

small and blurred brownish blotches.

Distribution and habitat. Currently known from Boluo County, Guangdong Province, belonging to Dong River, a tributary of the Pearl River (Figure 1). It was found in the streams and mainstem of moderate rivers in this river basin.

Etymology. The specific epithet is derived from the Greek prefix *pseudo*-, in allusion to the close morphological and phylogenetic affinities of *T. pseudovirgatus* with *T. virgatus*. The onomatopoeic Chinese sound of this species is “Si Zong Wen Ni Chang”.

Tachysurus bailongensis **sp. nov.**

(Figure 10)

Holotype. FANGC12597, 71.02 mm SL, South China: Guangxi Zhuang Automatus Region: Fangchenggang City: Naguo Town: Fangcheng River, a coastal river flowing into the Beibu Gulf (Figure 1); collected by Weihan Shao in Sept., 2021.

Paratypes. FANGC12592-6, FANGC12598, seven ex., 61.3−76.8 mm SL; other data same as holotype; DONGX 65305-6, two ex. 42.3−62.2 mm SL, South China: Guangxi Zhuang Automatus Region: Dongxing City: Jiangpiung Town: Jiangping River, a coastal river flowing into the Beibu Gulf.

Diagnosis. A striped *Tachysurus* species can be easily distinguished from non-striped congeners by the coloration consisting of two black, longitudinal and continuous stripes extending from the head to the caudal peduncle. Distinguished from all other striped congeners except *T. pseudovirgatus*, *T. virgatus*, *T. trimaculatus* and *T. furcatus* by the following combination of characters: a deeply (vs. moderately) forked caudal fin, a straight (vs. gently convex) posterior edges of the caudal-fin lobes, presence (vs. absence) of a prominent post-dorsal-fin notch on the dorso-lateral stripe, and a uniform and unconstricted (vs. tapping, constricted and irregular) mid-lateral stripe. It can be differentiated from *T. trimaculatus*, *T. pseudovirgatus*, *T. virgatus* and *T. furcatus* by shallower head (depth 53.4–59.1% HL vs. 58.6–71.1% HL) and narrower head (width 59.8–68.5% HL vs. 66.4–76.7% HL), from *T. pseudovirgatus*, *T. virgatus* and *T. furcatus* by the presence (vs.absence) of a connection between the

dorso-lateral stripe and black blotches on the operculum.

Description. See Figure 10 for general body appearance. Morphometric measurements taken from the holotype and paratypes summarized in Table S2.

Body elongated, anteriorly cylindrical and posteriorly slightly compressed. Dorsal profile rising evenly and steeply from tip of snout to origin of dorsal fin, sloping gently ventrally from origin of dorsal fin to end of caudal peduncle. Ventral surface of head flattened; Ventral profile slightly convex to anal-fin base, then sloping slightly dorsally to end of caudal peduncle. Lateral line complete, straight and mid-lateral in position. Vertebrae 5+31, (30, 32). Head depressed, broad, and covered with thin skin. Supraoccipital process slender, with evenly converging sides and pointed tip, separated from nuchal plate by broad interspace. Snout slightly pointed in dorsal view and obtuse or blunt in lateral view, longer than eye diameter. Interorbital space moderately wide and slightly flattish. Eyes moderately large, elliptical, covered with thick membrane, and anterolateral in head, visible when viewed dorsally, but not ventrally.

Mouth subterminal, transverse. Upper jaw anteriorly protruded, longer than lower jaw in length. Teeth villiform, in irregular rows on all toothbearing surfaces. Premaxillary tooth plates broad, of equal width throughout. Dentary tooth plates arched, broadest at symphysis and narrowing laterally, of same width at symphysis as premaxillary tooth plates. Vomerine tooth plate unpaired, continuous across midline, slightly curved anteriorly and much narrower than premaxillary plate. Gill opening wide, extending from post-temporal region to beyond isthmus.

Barbels in four pairs; nasal barbels small, thread-like, slightly extending posterior margin of eye, and maxillary barbels slender, not extending or slightly exceeding base of petoral-fin. Mandibular barbels in two pairs, thick, short; inner barbels positioned in transverse row at level of posterior naris, slightly extending beyond posterior of eye, and outer barbels rooted posterolateral to inner mandibular barbel, significantly extending to posterior margin of eye.

Dorsal fin with a spine and 7 soft branched rays. Dorsal-fin origin close to pectoral-fin insertion than pelvic-fin insertion, equidistant to anal-fin origin and snout

tip. Dorsal fin spine slender, with smooth posterior edge, equal to or slightly longer than pectoral-fin spine. First dorsal-fin soft ray longest, surpassing tip of last ray. Distal margin of dorsal-fin rays nearly straight. Nuchal plate triangular, with anterior pointed tip.

Adipose fin inserted slightly behind anal fin origin, with convex distal margin along entire length and deeply incised posterior part to form rounded apex. Adipose fin base short, significantly shorter than anal fin base length.

Pectoral fin with a spine and 7 soft branched rays, inserted slightly anterior or at level of posteriormost point of opercle, not reaching halfway to pelvic-fin insertion. Pectoral fin spine very stout, sharply pointed at tip, with a smooth anterior margin and 11 (10, 12 mean 10.9) strong serrations along posterior margin. Cleithral process triangular with a sharp pointed tip, extending to half of pectoral-fin spine length.

Pelvic fin with one unbranched and five branched soft rays, inserted closer to posterior end of anal fin base than to tip of snout, closer to anterior end of anal fin base than to depressed tip of dorsal fin. Tip of depressed pelvic fin reaching or slightly extending beyond anal fin origin. Pelvic fin distal margin convex. Anus and urogenital opening nearer to anal fin origin than to posterior end of pelvic fin base. Males with a conical genital papilla not reaching base of first anal fin soft ray.

Anal fin long, with 14 (13, 15) branched rays; inserted anterior to adipose fin origin. Anal fin origin nearer to caudal-fin base than to tip of snout. Distal margin of anal fin convex; anterior rays shortest.

Caudal fin with 9+11 principal rays, deeply forked, with middle rays shorter than two thirds of longest rays; both lobes having a straight posterior edges, with upper lobe equidistant to lower lobe; procurrent rays slightly extending from anterior to fin base. Shallowest point of caudal peduncle behind posterior end of anal fin base.

Coloration. Body gray, with two longitudinal black bands along sides. Mid-lateral stripe running along lateral line, black, continuous, and uniform; bifurcating at caudal base into two black narrow branches extending along the upper and lower caudal-fin lobes to their tips. Each branch occupies three fin rays. Dorsum with a broad black stripe, connecting with mid-lateral stripe anterior to dorsal fin and with the black

blotches on the upper margin of the operculum. Three irregular grayish blotches present on ventral quarter of body, arranged in series from post-temporal region to caudal-fin base. Nape with a broad and yellow-whitish band, extending downwards from head to middle of eye. The anterior portion of the mid-lateral stripe is broadly connected with the black blotches on the operculum. A vertical yellow-whitish band present posterior to the eye, extending from the dorsal margin of the eye to the ventral surface. Operculum with a short, narrow whitish-yellow luminescent patch along its upper margin.

Adipose fin cream, with a scatted brown melanophores forming a brown band at the anterior two thirds of fin and a slightly hyaline posterodorsal margin. Dorsal fin brown on distal half, rest of fin hyaline. Pectoral, pelvic and anal fins hyaline with small and blurred brownish blotches.

Distribution and habitat. Currently known from the Fangcheng River and Jiangping River in Guangxi Zhuang Autonomous Region, China, which are two coastal rivers flowing into the Beibu Gulf basin (Figure 1). Juveniles occur in streams, while adults are restricted to the main channels of rivers.

Etymology. The specific name bailongensis refers to "Bailongwei", the locality in Fangchenggang City, Guangxi Zhuang Autonomous Region, southern China, where the new species was collected. The name commemorates the historical site of the Bailong Fort, which was constructed by the Qing Dynasty in the late 19th century to defend the Chinese-Vietnamese border against incursions by French colonial forces in Vietnam.

***Tachysurus furcatus* sp. nov.**

(Figure 11)

Holotype. GUANGP63012, 67.40 mm SL, Central Vietnam: Quang Binh Prov.: Nhat Le River, a coastal river flowing into South China Sea (Figure 1); collected by Weihan Shao in June, 2025.

Paratypes. GUANGP63001-11, eleven ex., 52.7−73.3 mm SL; other data same as

holotype.

Diagnosis. A striped *Tachysurus* species can be easily distinguished from non-striped congeners by the coloration consisting of two black, longitudinal and continuous stripes extending from the head to the caudal peduncle. Distinguished from all other striped congeners except *T. pseudovirgatus*, *T. virgatus*, *T. trimaculatus* and *T. bailongensis* by the following combination of characters: a straight (vs. gently convex) posterior edges of the caudal-fin lobes, a deeply (vs. moderately) forked caudal fin, presence (vs. absence) of a prominent post-dorsal-fin notch on the dorsolateral stripe and a uniform and unconstricted (vs. tapering, constricted and irregular) mid-lateral stripe. It can be differentiated from *T. virgatus*, *T. trimaculatus* and *T. bailongensis* by the origin of the mid-lateral stripe diverging into two or three short black lines (vs. the entire lower portion) connecting to the black blotches on the operculum, from *T. trimaculatus* and *T. bailongensis* by the absence (vs. presence) of a connection between the dorso-lateral stripe and the black blotches on the operculum, from *T. pseudovirgatus* in having a deeper caudal peduncle (depth 59.5−71.1% vs. 42.1−45.3% HL).

Description. See Figure 11 for general body appearance. Morphometric measurements taken from the holotype and paratypes summarized in Table S2.

Body elongated, anteriorly cylindrical and posteriorly slightly compressed. Dorsal profile rising evenly and steeply from tip of snout to origin of dorsal fin, sloping gently ventrally from origin of dorsal fin to end of caudal peduncle. Ventral surface of head flattened; Ventral profile slightly convex to anal-fin base, then sloping slightly dorsally to end of caudal peduncle. Lateral line complete, straight and mid-lateral in position. Vertebrae 5+31, (30, 32). Head depressed, broad, and covered with thin skin. Supraoccipital process slender, with evenly converging sides and pointed tip, separated from nuchal plate by broad interspace. Snout slightly pointed in dorsal view and obtuse or blunt in lateral view, longer than eye diameter. Interorbital space moderately wide and slightly flattish. Eyes moderately large, elliptical, covered with thick membrane, and anterolateral in head, visible when viewed dorsally, but not ventrally.

Mouth subterminal, transverse. Upper jaw anteriorly protruded, longer than lower jaw in length. Teeth villiform, in irregular rows on all toothbearing surfaces. Premaxillary tooth plates broad, of equal width throughout. Dentary tooth plates arched, broadest at symphysis and narrowing laterally, of same width at symphysis as premaxillary tooth plates. Vomerine tooth plate unpaired, continuous across midline, slightly curved anteriorly and much narrower than premaxillary plate. Gill opening wide, extending from post-temporal region to beyond isthmus.

Barbels in four pairs; nasal barbels small, thread-like, slightly extending posterior margin of eye, and maxillary barbels slender, not extending or slightly exceeding base of petoral-fin. Mandibular barbels in two pairs, thick, short; inner barbels positioned in transverse row at level of posterior naris, slightly extending beyond posterior of eye, and outer barbels rooted posterolateral to inner mandibular barbel, significantly extending to posterior margin of eye.

Dorsal fin with a spine and 7 soft branched rays. Dorsal-fin origin close to pectoral-fin insertion than pelvic-fin insertion, equidistant to anal-fin origin and snout tip. Dorsal fin spine slender, with smooth posterior edge, equal to or slightly longer than pectoral-fin spine. First dorsal-fin soft ray longest, surpassing tip of last ray. Distal margin of dorsal-fin rays nearly straight. Nuchal plate triangular, with anterior pointed tip.

Adipose fin inserted slightly behind anal fin origin, with convex distal margin along entire length and deeply incised posterior part to form rounded apex. Adipose fin base short, significantly shorter than anal fin base length.

Pectoral fin with a spine and 7 soft branched rays, inserted slightly anterior or at level of posteriormost point of opercle, not reaching halfway to pelvic-fin insertion. Pectoral fin spine very stout, sharply pointed at tip, with a smooth anterior margin and 10 (9, 11 mean 10.6) strong serrations along posterior margin. Cleithral process triangular with a sharp pointed tip, extending to half of pectoral-fin spine length.

Pelvic fin with one unbranched and five branched soft rays, inserted closer to posterior end of anal fin base than to tip of snout, closer to anterior end of anal fin base than to depressed tip of dorsal fin. Tip of depressed pelvic fin reaching or slightly

extending beyond anal fin origin. Pelvic fin distal margin convex. Anus and urogenital opening nearer to anal fin origin than to posterior end of pelvic fin base. Males with a conical genital papilla not reaching base of first anal fin soft ray.

Anal fin long, with 14 (13) branched rays; inserted anterior to adipose fin origin. Anal fin origin nearer to caudal-fin base than to tip of snout. Distal margin of anal fin convex; anterior rays shortest.

Caudal fin with 9+11 principal rays, deeply forked, with middle rays shorter than two thirds of longest rays; both lobes narrow and straight, with upper lobe equidistant to lower lobe; procurrent rays slightly extending from anterior to fin base. Shallowest point of caudal peduncle behind posterior end of anal fin base.

Coloration. Body gray, with two longitudinal black bands along sides. Mid-lateral stripe running along lateral line, black, continuous, and uniform (in some individuals tapering progressively from dorsal to caudal fin); bifurcating at caudal base into two black narrow branches extending along the upper and lower caudal-fin lobes to their tips. Each branch occupies three fin rays. Dorsum with a broad black stripe, connecting with mid-lateral stripe anterior to dorsal fin. Three irregular grayish blotches present on ventral quarter of body, arranged in series from post-temporal region to caudal-fin base. Nape with a broad and yellow-whitish band, extending downwards from head to middle of eye. The anterior portion of the mid-lateral stripe is broadly connected with the black blotches on the operculum. A vertical yellow-whitish band present posterior to the eye, extending from the dorsal margin of the eye to the ventral surface. Operculum with a short, narrow whitish-yellow luminescent patch along its upper margin.

Adipose fin cream, with a scatted brown melanophores forming a brown band at the anterior two thirds of fin and a slightly hyaline posterodorsal margin. Dorsal fin brown on distal half, rest of fin hyaline. Pectoral, pelvic and anal fins hyaline with small and blurred brownish blotches.

Distribution and habitat. Currently known from the Nhat Le River in Quang Binh Province, and Hương River in Thừa Thiên Huế Province, central Vietnam, which are two coastal rivers flowing into the South China Sea (Figure 1). Juveniles occur in

streams, while adults are restricted to the main channels of rivers.

Etymology. The specific epithet *furcatus* is derived from Latin, meaning “forked” , referring to the distinctive mid-lateral stripe of this species, which diverges into two or three short black lines to connect to the black blotches on the operculum, forming a fork-like pattern. The species is locally known in Chinese as “Cha Wen Ni Chang”.

Tachysurus trimaculatus **sp. nov.**

(Figure 12)

Holotype. PINGHE65311, 69.57 mm SL, Southeast China: Fujian Prov.:Pinghe Town: Jiulong River, a coastal river flowing into Taiwan Strait (Figure 1); collected by Weihan Shao in June, 2019.

Paratypes. PINGHE 65312-7, NANJ9279-9286, fourteen ex., 46.18−71.37 mm SL; other data same as holotype.

Diagnosis. A striped *Tachysurus* species can be easily distinguished from non-striped congeners by the coloration consisting of two black, longitudinal and continuous stripes extending from the head to the caudal peduncle. Distinguished from all other striped congeners by the following combination of characters: a deeply (vs. moderately) forked caudal fin, presence (vs. absence) of a connection between the dorso-lateral stripe and the black blotches on the upper margin of the operculum; and absence (vs. presence) of a connection between the dorso-lateral and mid-lateral stripes anterior to the dorsal fin.

Description. See Figure 12 for general body appearance. Morphometric measurements taken from the holotype and paratypes summarized in Table S2.

Body elongated, anteriorly cylindrical and posteriorly slightly compressed. Dorsal profile rising evenly and steeply from tip of snout to origin of dorsal fin, sloping gently ventrally from origin of dorsal fin to end of caudal peduncle. Ventral surface of head flattened; Ventral profile slightly convex to anal-fin base, then sloping slightly dorsally to end of caudal peduncle. Lateral line complete, straight and mid-lateral in position. Vertebrae 5+31, (30). Head depressed, broad, and covered with thin skin. Supraoccipital process slender, with evenly converging sides and

pointed tip, separated from nuchal plate by broad interspace. Snout slightly pointed in dorsal view and obtuse or blunt in lateral view, longer than eye diameter. Interorbital space moderately wide and slightly flattish. Eyes moderately large, elliptical, covered with thick membrane, and anterolateral in head, visible when viewed dorsally, but not ventrally.

Mouth subterminal, transverse. Upper jaw anteriorly protruded, longer than lower jaw in length. Teeth villiform, in irregular rows on all toothbearing surfaces. Premaxillary tooth plates broad, of equal width throughout. Dentary tooth plates arched, broadest at symphysis and narrowing laterally, of same width at symphysis as premaxillary tooth plates. Vomerine tooth plate unpaired, continuous across midline, slightly curved anteriorly and much narrower than premaxillary plate. Gill opening wide, extending from post-temporal region to beyond isthmus.

Barbels in four pairs; nasal barbels small, thread-like, slightly extending posterior margin of eye, and maxillary barbels slender, not extending or slightly exceeding base of petoral-fin. Mandibular barbels in two pairs, thick, short; inner barbels positioned in transverse row at level of posterior naris, slightly extending beyond posterior of eye, and outer barbels rooted posterolateral to inner mandibular barbel, significantly extending to posterior margin of eye.

Dorsal fin with a spine and 7 soft branched rays. Dorsal-fin origin close to pectoral-fin insertion than pelvic-fin insertion, equidistant to anal-fin origin and snout tip. Dorsal fin spine slender, with smooth posterior edge, equal to or slightly longer than pectoral-fin spine. First dorsal-fin soft ray longest, surpassing tip of last ray. Distal margin of dorsal-fin rays nearly straight. Nuchal plate triangular, with anterior pointed tip.

Adipose fin inserted slightly behind anal fin origin, with convex distal margin along entire length and deeply incised posterior part to form rounded apex. Adipose fin base short, significantly shorter than anal fin base length.

Pectoral fin with a spine and 7 soft branched rays, inserted slightly anterior or at level of posteriormost point of opercle, not reaching halfway to pelvic-fin insertion. Pectoral fin spine very stout, sharply pointed at tip, with a smooth anterior margin and

10 (9, 11 mean 10.6) strong serrations along posterior margin. Cleithral process triangular with a sharp pointed tip, extending to half of pectoral-fin spine length.

Pelvic fin with one unbranched and five branched soft rays, inserted closer to posterior end of anal fin base than to tip of snout, closer to anterior end of anal fin base than to depressed tip of dorsal fin. Tip of depressed pelvic fin reaching or slightly extending beyond anal fin origin. Pelvic fin distal margin convex. Anus and urogenital opening nearer to anal fin origin than to posterior end of pelvic fin base. Males with a conical genital papilla not reaching base of first anal fin soft ray.

Anal fin long, with 13 (14) branched rays; inserted anterior to adipose fin origin. Anal fin origin nearer to caudal-fin base than to tip of snout. Distal margin of anal fin convex; anterior rays shortest.

Caudal fin with 9+11 principal rays, deeply forked, with middle rays shorter than two thirds of longest rays; both lobes rounded, with upper lobe equidistant to lower lobe; procurrent rays slightly extending from anterior to fin base. Shallowest point of caudal peduncle behind posterior end of anal fin base.

Coloration. Body gray, with two longitudinal black bands along sides. Mid-lateral stripe running along lateral line, black, continuous, and uniform; bifurcating at caudal base into two black narrow branches extending along the upper and lower caudal-fin lobes to their tips. Each branch occupies three fin rays. Dorsum with a broad black stripe, not connecting with mid-lateral stripe anterior to dorsal fin, continuous with black blotches on the upper margin of the operculum. Three irregular grayish blotches present on ventral quarter of body, arranged in series from post-temporal region to caudal-fin base. Nape with a broad and yellow-whitish band, extending downwards from head to middle of eye. The anterior portion of the mid-lateral stripe is broadly connected with the black blotches on the operculum. A vertical yellow-whitish band present posterior to the eye, extending from the dorsal margin of the eye to the ventral surface. Operculum with a short, narrow whitish-yellow luminescent patch along its upper margin.

Adipose fin cream, with a scatted brown melanophores forming a brown band at the anterior two thirds of fin and a slightly hyaline posterodorsal margin. Dorsal fin

brown on distal half, rest of fin hyaline. Pectoral, pelvic and anal fins hyaline with small and blurred brownish blotches.

Distribution and habitat. Currently known from the Beixi River, a tributary of the Jiulongjiang River in Fujian Province, Southeast China, which is coastal river flowing into the Taiwan Strait (Figure 1). Recorded exclusively in flowing streams.

Etymology. The specific epithet is derived from the Latin *tri-* ("three") and *maculatus* ("spotted"), referring to the distinctive nuchal stripe of this species. In dorsal view, the nuchal white-yellowish stripe, typically continuous in congeners, is uniquely separated into three blotches. The onomatopoeic Chinese sound of this species is "San Ban Ni Chang".

Tachysurus platystomus **sp. nov.**

(Figure 13)

Holotype. HAIF01, 88.84 mm SL, South China: Guangdong Prov.: Haifeng County: Huang River, a coastal river flowing into South China Sea (Figure 1); collected by Weihan Shao in July, 2020.

Paratypes. HAIF02-8, seven ex., 34.15−137.54 mm SL; other data same as holotype.

Diagnosis. A striped *Tachysurus* species can be easily distinguished from non-striped congeners by the coloration consisting of two black, longitudinal and continuous stripes extending from the head to the caudal peduncle. Distinguished from all other striped congeners except *T. heterofasciatus* by the moderately (vs. deeply) forked caudal fin. It can be further differentiated from *T. heterofasciatus* in having a shorter dorsal spine (length 15.7−20.3% SL vs. 19.1−24.4%), a wider mouth (width 12.3−14.7% SL vs. 8.9−11.7%) and a mid-lateral stripe with slight (vs. distinct) constriction at the posterior end of the dorsal and adipose fins.

Description. See Figure 13 for general body appearance. Morphometric measurements taken from the holotype and paratypes summarized in Table S3.

Body elongated, anteriorly cylindrical and posteriorly slightly compressed. Dorsal profile rising evenly and steeply from tip of snout to origin of dorsal fin,

sloping gently ventrally from origin of dorsal fin to end of caudal peduncle. Ventral surface of head flattened; Ventral profile slightly convex to anal-fin base, then sloping slightly dorsally to end of caudal peduncle. Lateral line complete, straight and mid-lateral in position. Vertebrae 5+35, (36). Head depressed, broad, and covered with thin skin. Supraoccipital process slender, with evenly converging sides and pointed tip, separated from nuchal plate by broad interspace. Snout slightly pointed in dorsal view and obtuse or blunt in lateral view, longer than eye diameter. Interorbital space moderately wide and slightly flattish. Eyes moderately large, elliptical, covered with thick membrane, and anterolateral in head, visible when viewed dorsally, but not ventrally.

Mouth slightly subterminal, transverse. Upper jaw anteriorly protruded, longer than lower jaw in length. Teeth villiform, in irregular rows on all toothbearing surfaces. Premaxillary tooth plates broad, of equal width throughout. Dentary tooth plates arched, broadest at symphysis and narrowing laterally, of same width at symphysis as premaxillary tooth plates. Vomerine tooth plate unpaired, continuous across midline, slightly curved anteriorly and much narrower than premaxillary plate. Gill opening wide, extending from post-temporal region to beyond isthmus.

Barbels in four pairs; nasal barbels small, thread-like, not extending or slightly extending posterior margin of eye, and maxillary barbels slender, not extending base of petoral-fin. Mandibular barbels in two pairs, thick, short; inner barbels positioned in transverse row at level of posterior naris, not extending beyond posterior of eye, and outer barbels rooted posterolateral to inner mandibular barbel, slightly extending to posterior margin of eye.

Dorsal fin with a spine and 7 soft branched rays. Dorsal-fin origin close to pectoral-fin insertion than pelvic-fin insertion, equidistant to anal-fin origin and snout tip. Dorsal fin spine slender, with smooth posterior edge, equal to or slightly longer than pectoral-fin spine. First dorsal-fin soft ray longest, surpassing tip of last ray. Distal margin of dorsal-fin rays nearly straight. Nuchal plate triangular, with anterior pointed tip.

Adipose fin inserted slightly behind anal fin origin, with convex distal margin

along entire length and deeply incised posterior part to form rounded apex. Adipose fin base short, significantly shorter than anal fin base length.

Pectoral fin with a spine and 7 soft branched rays, inserted slightly anterior or at level of posteriormost point of opercle, not reaching halfway to pelvic-fin insertion. Pectoral fin spine very stout, sharply pointed at tip, with a smooth anterior margin and 12 (11 and 13, mean 12.2) strong serrations along posterior margin. Cleithral process triangular with a sharp pointed tip, extending to half of pectoral-fin spine length.

Pelvic fin with one unbranched and five branched soft rays, inserted closer to posterior end of anal fin base than to tip of snout, closer to anterior end of anal fin base than to depressed tip of dorsal fin. Tip of depressed pelvic fin reaching or slightly extending beyond anal fin origin. Pelvic fin distal margin convex. Anus and urogenital opening nearer to anal fin origin than to posterior end of pelvic fin base. Males with a conical genital papilla not reaching base of first anal fin soft ray.

Anal fin long, with 16 (15, 17) branched rays; inserted anterior to adipose fin origin. Anal fin origin nearer to caudal-fin base than to tip of snout. Distal margin of anal fin convex; anterior rays shortest.

Caudal fin with 8+10 principal rays, moderately forked , with middle rays longer than two thirds of longest rays; both lobes rounded, with upper lobe equidistant to lower lobe; procurrent rays slightly extending from anterior to fin base. Shallowest point of caudal peduncle behind posterior end of anal fin base.

Coloration. Body gray, with two longitudinal black bands along sides. Mid-lateral stripe running along lateral line, black, continuous, and slightly constricted at the ; bifurcating at caudal base into two black wide and obscured branches extending along the upper and lower caudal-fin lobes to their tips. Each branch occupies four to fiver fin rays. Dorsum with a broad black stripe, connecting with mid-lateral stripe anterior to dorsal fin. Three irregular grayish blotches present on ventral quarter of body, arranged in series from post-temporal region to caudal-fin base. Nape with a moderately broad yellow-whitish band, extending downward from the head to the middle of the eye, which is obscured in large individuals extending 120 mm SL. In smaller individuals, the anterior portion of the mid-lateral stripe is slightly connected

to the black blotches on the operculum, whereas in larger individuals this connection is largely developed. A vertical yellow-whitish band present posterior to the eye, extending from the dorsal margin of the eye to the ventral surface. Operculum with a short, narrow whitish-yellow luminescent patch along its upper margin.

Adipose fin cream, with a scatted brown melanophores forming a brown band at the anterior two thirds of fin and a slightly hyaline posterodorsal margin. Dorsal fin brown on distal half, rest of fin hyaline. Pectoral, pelvic and anal fins hyaline with small and blurred brownish blotches.

Distribution and habitat. Currently known from the Huangjiang River, in Haifeng County, Guangdong Province, South China, which is a coastal river flowing into South China Sea (Figure 1). Recorded exclusively in flowing streams.

Etymology. The specific name *platycephalus* is derived from the Greek *platy-* ("broad") and *-stomus* ("mouth"), referring to the broad mouth of this species, which is wider than that of all other striped *Tachysurus* species. The onomatopoeic Chinese sound of this species is"Kuankou Ni Chang".

***Tachysurus heterofasciatus* sp. nov.**

(Figure 14)

Holotype. YANGC47901, 102.53 mm SL, South China: Guangdong Prov.: Yangchun City: E'Huangzhang Natural Reserve: Moyangjiang River, a coastal river flowing into South China Sea (Figure 1); collected by Ke Zheng in July, 2024.

Paratypes. YANGC47902-11, ten ex., 57.79−156.92 mm SL; other data same as holotype.

Diagnosis. A striped *Tachysurus* species can be easily distinguished from non-striped congeners by the coloration consisting of two black, longitudinal and continuous stripes extending from the head to the caudal peduncle. Distinguished from all other striped congeners except *T. platecephalus* by the moderately (vs. deeply) forked caudal fin. It can be further differentiated from *T. platecephalus* in having a longer dorsal spine (length 19.1−24.4% vs. 15.7−20.3% SL), a narrower mouth (width 8.9−11.7% SL vs.12.3−14.7%) and a mid-lateral stripe distinctly (vs. slightly)

constricted at the posterior end of the dorsal and adipose fins.

Description. See Figure 14 for general body appearance. Morphometric measurements taken from the holotype and paratypes summarized in Table S3.

Body elongated, anteriorly cylindrical and posteriorly slightly compressed. Dorsal profile rising evenly and steeply from tip of snout to origin of dorsal fin, sloping gently ventrally from origin of dorsal fin to end of caudal peduncle. Ventral surface of head flattened; Ventral profile slightly convex to anal-fin base, then sloping slightly dorsally to end of caudal peduncle. Lateral line complete, straight and mid-lateral in position. Vertebrae 5+36, (35). Head depressed, broad, and covered with thin skin. Supraoccipital process slender, with evenly converging sides and pointed tip, separated from nuchal plate by broad interspace. Snout slightly pointed in dorsal view and obtuse or blunt in lateral view, longer than eye diameter. Interorbital space moderately wide and slightly flattish. Eyes moderately large, elliptical, covered with thick membrane, and anterolateral in head, visible when viewed dorsally, but not ventrally.

Mouth slightly subterminal, transverse. Upper jaw anteriorly protruded, longer than lower jaw in length. Teeth villiform, in irregular rows on all toothbearing surfaces. Premaxillary tooth plates broad, of equal width throughout. Dentary tooth plates arched, broadest at symphysis and narrowing laterally, of same width at symphysis as premaxillary tooth plates. Vomerine tooth plate unpaired, continuous across midline, slightly curved anteriorly and much narrower than premaxillary plate. Gill opening wide, extending from post-temporal region to beyond isthmus.

Barbels in four pairs; nasal barbels small, thread-like, not extending or slightly extending posterior margin of eye, and maxillary barbels slender, not extending base of petoral-fin. Mandibular barbels in two pairs, thick, short; inner barbels positioned in transverse row at level of posterior naris, not extending beyond posterior of eye, and outer barbels rooted posterolateral to inner mandibular barbel, slightly extending to posterior margin of eye.

Dorsal fin with a spine and 7 soft branched rays. Dorsal-fin origin close to pectoral-fin insertion than pelvic-fin insertion, equidistant to anal-fin origin and snout

tip. Dorsal fin spine slender, with smooth posterior edge, equal to or slightly longer than pectoral-fin spine. First dorsal-fin soft ray longest, surpassing tip of last ray. Distal margin of dorsal-fin rays nearly straight. Nuchal plate triangular, with anterior pointed tip.

Adipose fin inserted slightly behind anal fin origin, with convex distal margin along entire length and deeply incised posterior part to form rounded apex. Adipose fin base short, significantly shorter than anal fin base length.

Pectoral fin with a spine and 7 soft branched rays, inserted slightly anterior or at level of posteriormost point of opercle, not reaching halfway to pelvic-fin insertion. Pectoral fin spine very stout, sharply pointed at tip, with a smooth anterior margin and 14 (15 and 17 mean 15.1) strong serrations along posterior margin. Cleithral process triangular with a sharp pointed tip, extending to half of pectoral-fin spine length.

Pelvic fin with one unbranched and five branched soft rays, inserted closer to posterior end of anal fin base than to tip of snout, closer to anterior end of anal fin base than to depressed tip of dorsal fin. Tip of depressed pelvic fin reaching or slightly extending beyond anal fin origin. Pelvic fin distal margin convex. Anus and urogenital opening nearer to anal fin origin than to posterior end of pelvic fin base. Males with a conical genital papilla not reaching base of first anal fin soft ray.

Anal fin long, with 15 (16,17) branched rays; inserted anterior to adipose fin origin. Anal fin origin nearer to caudal-fin base than to tip of snout. Distal margin of anal fin convex; anterior rays shortest.

Caudal fin with 8(9)+10 principal rays, moderately forked, with middle rays longer than two thirds of longest rays; both lobes rounded, with upper lobe equidistant to lower lobe; procurrent rays slightly extending from anterior to fin base. Shallowest point of caudal peduncle behind posterior end of anal fin base.

Coloration. Body gray, with two longitudinal black bands along sides. Mid-lateral stripe running along lateral line, black, continuous, and constricted at two points, the posterior end of the dorsal fin and the adipose fin; bifurcating at caudal base into two black wide and obscured branches extending along the upper and lower caudal-fin lobes to their tips. Each branch occupies four to fiver fin rays. Dorsum with a broad

black stripe, connecting with mid-lateral stripe anterior to dorsal fin. Three irregular grayish blotches present on ventral quarter of body, arranged in series from post-temporal region to caudal-fin base. Nape with a narrow yellow-whitish band, extending downward from the head to the ventral region and interrupted by a short black line originating from the upper margin of the mid-lateral stripe. This band is obscured in large individuals extending to 120 mm SL. In smaller individuals, the anterior portion of the mid-lateral stripe is slightly connected to the black blotches on the operculum, whereas in larger individuals this connection is largely developed. A vertical yellow-whitish band present posterior to the eye, extending from the dorsal margin of the eye to the ventral surface. Operculum with a short, narrow whitish-yellow luminescent patch along its upper margin.

Adipose fin cream, with a scatted brown melanophores forming a brown band at the anterior two thirds of fin and a slightly hyaline posterodorsal margin. Dorsal fin brown on distal half, rest of fin hyaline. Pectoral, pelvic and anal fins hyaline with small and blurred brownish blotches.

Distribution and habitat. Currently known from the E' Huangzhang Natural Reserve, Moyang River, in Yangchun City, Guangdong Province, South China, which is a coastal river flowing into South China Sea (Figure 1). Recorded exclusively in flowing streams.

Etymology. The specific epithet is derived from the Greek *hetero-* ("different") and Latin *-fasciatus* ("banded"), referring to the species' unique mid-lateral stripe, which is of uniform width but interrupted by two pronounced constrictions, in contrast to other striped species, where the mid-lateral stripe is either uniformly wide without constrictions or gradually narrows with constrictions. The onomatopoeic Chinese sound of this species is "Yi Wen Ni Chang".

***Tachysurus analimaculatus* sp. nov.**

(Figure 15)

Holotype. GUANGZ37486, 72.40 mm SL, China: Guangdong Prov.: Guangzhou City, Zhongluotan Town, Beijiang River, a tributary of the Pearl River (Figure 1); collected

by Weihan Shao in March, 2020.

Paratypes. GUANGZ37481-5, five ex., 56.68−87.19 mm SL, other data same as holotype; GUIGANG65213-7, five ex., 41.00−51.91 mm SL, China: Guangxi Zhuang Autonomous Region: Guigang City, Xijiang River, a tributary of the Pearl River.

Diagnosis. A striped *Tachysurus* species can be easily distinguished from non-striped congeners by the coloration consisting of two black, longitudinal and continuous stripes extending from the head to the caudal peduncle. Distinguished from all other striped congeners except *T. qiongzhouensis* and *T. sinovietnamensis* by the following combination of characters: a deeply (vs. moderately) forked caudal fin, presence (vs. absence) of a prominent post-dorsal-fin notch on the dorsolateral stripe and a constricted, irregular and tapping (vs. unconstricted and uniformly wide) mid-lateral stripe. It can be differentiated from *T. qiongzhouensis* and *T. sinovietnamensis* in having a more slender body (depth at anus 13.8–19.2% HL vs. 19.1–24.5%). It further differs from *T. qiongzhouensis* by having a shorter nasal barbel (30.6–44.8% HL vs. 44.1–69.8%), and from *T. sinovietnamensis* by having a more slender caudal peduncle (depth 15.2–17.6% pre-anal length vs. 12.6–15.7%).

Description. See Figure 15 for general body appearance. Morphometric measurements taken from the holotype and paratypes summarized in Table S4.

Body elongated, anteriorly cylindrical and posteriorly slightly compressed. Dorsal profile rising evenly and steeply from tip of snout to origin of dorsal fin, sloping gently ventrally from origin of dorsal fin to end of caudal peduncle. Ventral surface of head flattened; Ventral profile slightly convex to anal-fin base, then sloping slightly dorsally to end of caudal peduncle. Lateral line complete, straight and mid-lateral in position. Vertebrae 5+32 (33). Head depressed, broad, and covered with thin skin. Supraoccipital process slender, with evenly converging sides and pointed tip, separated from nuchal plate by broad interspace. Snout slightly pointed in dorsal view and obtuse or blunt in lateral view, longer than eye diameter. Interorbital space moderately wide and slightly flattish. Eyes moderately large, elliptical, covered with thick membrane, and anterolateral in head, visible when viewed dorsally, but not ventrally.

Mouth subterminal, transverse. Upper jaw anteriorly protruded, longer than lower jaw in length. Teeth villiform, in irregular rows on all toothbearing surfaces. Premaxillary tooth plates broad, of equal width throughout. Dentary tooth plates arched, broadest at symphysis and narrowing laterally, of same width at symphysis as premaxillary tooth plates. Vomerine tooth plate unpaired, continuous across midline, slightly curved anteriorly and much narrower than premaxillary plate. Gill opening wide, extending from post-temporal region to beyond isthmus.

Barbels in four pairs; nasal barbels small, thread-like, slightly extending posterior margin of eye, and maxillary barbels slender, not extending or slightly exceeding base of petoral-fin. Mandibular barbels in two pairs, thick, short; inner barbels positioned in transverse row at level of posterior naris, slightly extending beyond posterior of eye, and outer barbels rooted posterolateral to inner mandibular barbel, significantly extending to posterior margin of eye.

Dorsal fin with a spine and 7 soft branched rays. Dorsal-fin origin close to pectoral-fin insertion than pelvic-fin insertion, equidistant to anal-fin origin and snout tip. Dorsal fin spine slender, with smooth posterior edge, equal to or slightly longer than pectoral-fin spine. First dorsal-fin soft ray longest, surpassing tip of last ray. Distal margin of dorsal-fin rays nearly straight. Nuchal plate triangular, with anterior pointed tip.

Adipose fin inserted slightly behind anal fin origin, with convex distal margin along entire length and deeply incised posterior part to form rounded apex. Adipose fin base short, significantly shorter than anal fin base length.

Pectoral fin with a spine and 7 soft branched rays, inserted slightly anterior or at level of posteriormost point of opercle, not reaching halfway to pelvic-fin insertion. Pectoral fin spine very stout, sharply pointed at tip, with a smooth anterior margin and 10 (9, 11 mean 10.5) strong serrations along posterior margin. Cleithral process triangular with a sharp pointed tip, extending to half of pectoral-fin spine length.

Pelvic fin with one unbranched and five branched soft rays, inserted closer to posterior end of anal fin base than to tip of snout, closer to anterior end of anal fin base than to depressed tip of dorsal fin. Tip of depressed pelvic fin reaching or slightly

extending beyond anal fin origin. Pelvic fin distal margin convex. Anus and urogenital opening nearer to anal fin origin than to posterior end of pelvic fin base. Males with a conical genital papilla not reaching base of first anal fin soft ray.

Anal fin long, with 16 (15) branched rays; inserted anterior to adipose fin origin. Anal fin origin nearer to caudal-fin base than to tip of snout. Distal margin of anal fin convex; anterior rays shortest.

Caudal fin with 9+11 principal rays, deeply forked, with middle rays shorter than two thirds of longest rays; both lobes narrow and straight, with upper lobe equidistant to lower lobe; procurrent rays slightly extending from anterior to fin base. Shallowest point of caudal peduncle behind posterior end of anal fin base.

Coloration. Body gray, with two longitudinal black bands along sides. Mid-lateral stripe running along lateral line, black, continuous, and tapering progressively with two constriction at posterior margins of the dorsal and adipose fins; bifurcating at caudal base into two black narrow branches extending along the upper and lower caudal-fin lobes to their tips. Each branch occupies three fin rays. Dorsum with a broad black stripe, connecting with mid-lateral stripe anterior to dorsal fin. Three irregular grayish blotches present on ventral quarter of body, arranged in series from post-temporal region to caudal-fin base extending ventrally onto the pelvic and anal fins. Nape with a broad yellow-whitish band extending from the cephalic region to the ventral region, interrupted by two short black lines, which originate from the upper and lower margins of the anterior region of the mid-lateral stripe, linking to the opercular black blotches. This band becomes obscured in large individuals. A vertical yellow-whitish band present posterior to the eye, extending from the dorsal margin of the eye to the ventral surface. Operculum with a short, narrow whitish-yellow luminescent patch along its upper margin.

Adipose fin cream, with a scatted brown melanophores forming a brown band at the anterior two thirds of fin and a slightly hyaline posterodorsal margin. Dorsal fin brown on distal half, rest of fin hyaline. Pectoral, pelvic and anal fins hyaline with small and blurred brownish blotches.

Distribution and habitat. Currently known from the Pearl River, South China

(Figure 1). Inhabiting exclusively in main channels of rivers.

Etymology. The specific epithet "*analimaculatus*" is a compound adjective derived from the Latin terms *analis*- (pertaining to the anal fin) and *maculatus*- (spotted or marked with patches). This name refers to the prominent black blotch on the anal fin. The onomatopoeic Chinese sound of this species is "Tun Ban Ni Chang".

***Tachysurus qiongzhouensis* sp. nov.**

(Figure 16)

Holotype. HAIK37477, 88.29 mm SL, China: Hainan Prov.: Haikou City, Xinpo Town, Nandu River, a coastal river of Hainan Island (Figure 1); collected by Xingwei Cai in November, 2019.

Paratypes. HAIK37472-6, HAIK200304-8, ten ex., 56.56−98.28 mm SL, other data same as holotype; YANGC57807-10, 57813-14, five ex., 60.74−69.67 mm SL, China: Guangdong Prov.: Yangchun City, Heshui Town, Moyang River, a coastal river of south China.

Diagnosis. A striped *Tachysurus* species can be easily distinguished from non-striped congeners by the coloration consisting of two black, longitudinal and continuous stripes extending from the head to the caudal peduncle. Distinguished from all other striped congeners except *T. analimaculatus* and *T. sinovietnamensis* by the following combination of characters: a deeply (vs. moderately) forked caudal fin, presence (vs. absence) of a prominent post-dorsal-fin notch on the dorsolateral stripe and a constricted, irregular and tapering (vs. unconstricted and uniformly wide) mid-lateral stripe. It can be differentiated from *T. analimaculatus* in having a deepter body (depth at anus 19.1–24.5% vs. 13.8–19.2% HL). *Tachysurus qiongzhouensis* exhibits overall morphological similarity to *T. sinovietnamensis* but can be distinguished by the ratios of SL/HL to maxillary barbel length (25.28–51.1% vs. 46.86–88.76%) and SL/HL to nasal barbel length (21.58–34.90% vs. 32.01–46.53%).

Description. See Figure 16 for general body appearance. Morphometric measurements taken from the holotype and paratypes summarized in Table S4.

Body elongated, anteriorly cylindrical and posteriorly slightly compressed. Dorsal profile rising evenly and steeply from tip of snout to origin of dorsal fin, sloping gently ventrally from origin of dorsal fin to end of caudal peduncle. Ventral surface of head flattened; Ventral profile slightly convex to anal-fin base, then sloping slightly dorsally to end of caudal peduncle. Lateral line complete, straight and mid-lateral in position. Vertebrae 5+31 (32). Head depressed, broad, and covered with thin skin. Supraoccipital process slender, with evenly converging sides and pointed tip, separated from nuchal plate by broad interspace. Snout slightly pointed in dorsal view and obtuse or blunt in lateral view, longer than eye diameter. Interorbital space moderately wide and slightly flattish. Eyes moderately large, elliptical, covered with thick membrane, and anterolateral in head, visible when viewed dorsally, but not ventrally.

Mouth subterminal, transverse. Upper jaw anteriorly protruded, longer than lower jaw in length. Teeth villiform, in irregular rows on all toothbearing surfaces. Premaxillary tooth plates broad, of equal width throughout. Dentary =tooth plates arched, broadest at symphysis and narrowing laterally, of same width at symphysis as premaxillary tooth plates. Vomerine tooth plate unpaired, continuous across midline, slightly curved anteriorly and much narrower than premaxillary plate. Gill opening wide, extending from post-temporal region to beyond isthmus.

Barbels in four pairs; nasal barbels small, thread-like, slightly extending posterior margin of eye, and maxillary barbels slender, not extending or slightly exceeding base of petoral-fin. Mandibular barbels in two pairs, thick, short; inner barbels positioned in transverse row at level of posterior naris, slightly extending beyond posterior of eye, and outer barbels rooted posterolateral to inner mandibular barbel, significantly extending to posterior margin of eye.

Dorsal fin with a spine and 7 soft branched rays. Dorsal-fin origin close to pectoral-fin insertion than pelvic-fin insertion, equidistant to anal-fin origin and snout tip. Dorsal fin spine slender, with smooth posterior edge, equal to or slightly longer than pectoral-fin spine. First dorsal-fin soft ray longest, surpassing tip of last ray. Distal margin of dorsal-fin rays nearly straight. Nuchal plate triangular, with anterior

pointed tip.

Adipose fin inserted slightly behind anal fin origin, with convex distal margin along entire length and deeply incised posterior part to form rounded apex. Adipose fin base short, significantly shorter than anal fin base length.

Pectoral fin with a spine and 7 soft branched rays, inserted slightly anterior or at level of posteriormost point of opercle, not reaching halfway to pelvic-fin insertion. Pectoral fin spine very stout, sharply pointed at tip, with a smooth anterior margin and 10 (11, 12 mean 11.2) strong serrations along posterior margin. Cleithral process triangular with a sharp pointed tip, extending to half of pectoral-fin spine length.

Pelvic fin with one unbranched and five branched soft rays, inserted closer to posterior end of anal fin base than to tip of snout, closer to anterior end of anal fin base than to depressed tip of dorsal fin. Tip of depressed pelvic fin reaching or slightly extending beyond anal fin origin. Pelvic fin distal margin convex. Anus and urogenital opening nearer to anal fin origin than to posterior end of pelvic fin base. Males with a conical genital papilla not reaching base of first anal fin soft ray.

Anal fin long, with 16 (15, 17) branched rays; inserted anterior to adipose fin origin. Anal fin origin nearer to caudal-fin base than to tip of snout. Distal margin of anal fin convex; anterior rays shortest.

Caudal fin with 9+11 principal rays, deeply forked, with middle rays shorter than two thirds of longest rays; both lobes narrow and straight, with upper lobe equidistant to lower lobe; procurrent rays slightly extending from anterior to fin base. Shallowest point of caudal peduncle behind posterior end of anal fin base.

Coloration. Body gray, with two longitudinal black bands along sides. Mid-lateral stripe running along lateral line, black, continuous, and tapering progressively with two constriction at posterior margins of the dorsal and adipose fins; bifurcating at caudal base into two black narrow branches extending along the upper and lower caudal-fin lobes to their tips. Each branch occupies three to four fin rays, with the branch on the lower caudal-fin lobe thicker than that on the upper lobe. Dorsum with a broad black stripe, connecting with mid-lateral stripe anterior to dorsal fin. Three irregular grayish blotches occur on the ventral quarter of the body; black blotches are

present at the bases of the pelvic and anal fins but do not reach the fin margins. Nape with a broad yellow-whitish band extending from the cephalic region to the ventral region, interrupted by two short black lines, which originate from the upper and lower margins of the anterior region of the mid-lateral stripe, linking to the opercular black blotches. This band becomes obscured in large individuals. A vertical yellow-whitish band present posterior to the eye, extending from the dorsal margin of the eye to the ventral surface. Operculum with a short, narrow whitish-yellow luminescent patch along its upper margin.

Adipose fin cream, with a scatted brown melanophores forming a brown band at the anterior two thirds of fin and a slightly hyaline posterodorsal margin. Dorsal fin brown on distal half, rest of fin hyaline. Pectoral, pelvic and anal fins hyaline with small and blurred brownish blotches.

Distribution and habitat. Currently known from the Nandu and Changhua Rivers on Hainan Island and Moyang River in Guangdong Province, South China (Figure 1). Inhabiting exclusively in main channels of rivers.

Etymology. The specific epithet is derived from Qiongzhou, the historical designation for Hainan Island and the primary distributional range of this species. This species also inhabits the Moyang River in Guangdong Province, a region situated across the Qiongzhou Strait from Hainan Island. This epithet emphasizes the geographical affinity linking the populations of these two adjacent regions. The onomatopoeic Chinese sound of this species is “Qiong Zhou Ni Chang”.

***Tachysurus sinovietnamensis* sp. nov.**

(Figure 17)

Holotype. YUANY13, 71.90 mm SL, China: Yunnan Prov.: Yuanyang County, Red River (Figure 1); collected by Hongfu Yang in November, 2024.

Paratypes. YUANY11-12, 15-16, four ex., 50.01−69.91 mm SL, other data same as holotype; BN202501-4, four ex., 52.74−65.27 mm SL, Vietnam: Bắc Ninh Prov.: Bắc Ninh City, Cầu River, a tributary of Red River.

Diagnosis. A striped *Tachysurus* species can be easily distinguished from

non-striped congeners by the coloration consisting of two black, longitudinal and continuous stripes extending from the head to the caudal peduncle. Distinguished from all other striped congeners except *T. analimaculatus* and *T. sinovietnamensis* by the following combination of characters: a deeply (vs. moderately) forked caudal fin, presence (vs. absence) of a prominent post-dorsal-fin notch on the dorsolateral stripe and a constricted, irregular and tapering (vs. unconstricted and uniformly wide) mid-lateral stripe. It can be differentiated from *T. analimaculatus* in having a deepter body (depth at anus 19.1–24.5% vs. 13.8–19.2% HL). *Tachysurus sinovietnamensis* exhibits overall morphological similarity to *T. qiongzhouensis* but can be distinguished by the ratios of SL/HL to maxillary barbel length (46.86–88.76% vs. 25.28–51.1%) and SL/HL to nasal barbel length (32.01–46.53% vs. 21.58–34.90%).

Description. See Figure 17 for general body appearance. Morphometric measurements taken from the holotype and paratypes summarized in Table S4.

Body elongated, anteriorly cylindrical and posteriorly slightly compressed. Dorsal profile rising evenly and steeply from tip of snout to origin of dorsal fin, sloping gently ventrally from origin of dorsal fin to end of caudal peduncle. Ventral surface of head flattened; Ventral profile slightly convex to anal-fin base, then sloping slightly dorsally to end of caudal peduncle. Lateral line complete, straight and mid-lateral in position. Vertebrae 5+31 (32). Head depressed, broad, and covered with thin skin. Supraoccipital process slender, with evenly converging sides and pointed tip, separated from nuchal plate by broad interspace. Snout slightly pointed in dorsal view and obtuse or blunt in lateral view, longer than eye diameter. Interorbital space moderately wide and slightly flattish. Eyes moderately large, elliptical, covered with thick membrane, and anterolateral in head, visible when viewed dorsally, but not ventrally.

Mouth subterminal, transverse. Upper jaw anteriorly protruded, longer than lower jaw in length. Teeth villiform, in irregular rows on all toothbearing surfaces. Premaxillary tooth plates broad, of equal width throughout. Dentary tooth plates arched, broadest at symphysis and narrowing laterally, of same width at symphysis as premaxillary tooth plates. Vomerine tooth plate unpaired, continuous across midline,

slightly curved anteriorly and much narrower than premaxillary plate. Gill opening wide, extending from post-temporal region to beyond isthmus.

Barbels in four pairs; nasal barbels small, thread-like, slightly extending posterior margin of eye, and maxillary barbels slender, not extending or slightly exceeding base of petoral-fin. Mandibular barbels in two pairs, thick, short; inner barbels positioned in transverse row at level of posterior naris, slightly extending beyond posterior of eye, and outer barbels rooted posterolateral to inner mandibular barbel, significantly extending to posterior margin of eye.

Dorsal fin with a spine and 7 soft branched rays. Dorsal-fin origin close to pectoral-fin insertion than pelvic-fin insertion, equidistant to anal-fin origin and snout tip. Dorsal fin spine slender, with smooth posterior edge, equal to or slightly longer than pectoral-fin spine. First dorsal-fin soft ray longest, surpassing tip of last ray. Distal margin of dorsal-fin rays nearly straight. Nuchal plate triangular, with anterior pointed tip.

Adipose fin inserted slightly behind anal fin origin, with convex distal margin along entire length and deeply incised posterior part to form rounded apex. Adipose fin base short, significantly shorter than anal fin base length.

Pectoral fin with a spine and 7 soft branched rays, inserted slightly anterior or at level of posteriormost point of opercle, not reaching halfway to pelvic-fin insertion. Pectoral fin spine very stout, sharply pointed at tip, with a smooth anterior margin and 10 (9, 11 mean 10.1) strong serrations along posterior margin. Cleithral process triangular with a sharp pointed tip, extending to half of pectoral-fin spine length.

Pelvic fin with one unbranched and five branched soft rays, inserted closer to posterior end of anal fin base than to tip of snout, closer to anterior end of anal fin base than to depressed tip of dorsal fin. Tip of depressed pelvic fin reaching or slightly extending beyond anal fin origin. Pelvic fin distal margin convex. Anus and urogenital opening nearer to anal fin origin than to posterior end of pelvic fin base. Males with a conical genital papilla not reaching base of first anal fin soft ray.

Anal fin long, with 16 (15, 17) branched rays; inserted anterior to adipose fin origin. Anal fin origin nearer to caudal-fin base than to tip of snout. Distal margin of

anal fin convex; anterior rays shortest.

Caudal fin with 9+11 principal rays, deeply forked, with middle rays shorter than two thirds of longest rays; both lobes narrow and straight, with upper lobe equidistant to lower lobe; procurrent rays slightly extending from anterior to fin base. Shallowest point of caudal peduncle behind posterior end of anal fin base.

Coloration. Body gray, with two longitudinal black bands along sides. Mid-lateral stripe running along lateral line, black, continuous, and tapering progressively with two constriction at posterior margins of the dorsal and adipose fins; bifurcating at caudal base into two black narrow branches extending along the upper and lower caudal-fin lobes to their tips. Each branch occupies three to four fin rays, with the branch on the lower caudal-fin lobe thicker than that on the upper lobe. Dorsum with a broad black stripe, connecting with mid-lateral stripe anterior to dorsal fin. Three irregular grayish blotches occur on the ventral quarter of the body; black blotches are present at the bases of the pelvic and anal fins but do not reach the fin margins. Nape with a broad yellow-whitish band extending from the cephalic region to the ventral region, interrupted by two short black lines, which originate from the upper and lower margins of the anterior region of the mid-lateral stripe, linking to the opercular black blotches. This band becomes obscured in large individuals. A vertical yellow-whitish band present posterior to the eye, extending from the dorsal margin of the eye to the ventral surface. Operculum with a short, narrow whitish-yellow luminescent patch along its upper margin.

Adipose fin cream, with a scatted brown melanophores forming a brown band at the anterior two thirds of fin and a slightly hyaline posterodorsal margin. Dorsal fin brown on distal half, rest of fin hyaline. Pectoral, pelvic and anal fins hyaline with small and blurred brownish blotches.

Distribution and habitat. Currently known from the Red River in Southwest China and Northern Vietnam as well as coastal rivers in northern central Vietnam including Gianh and Lam Rivers in Quang Binh and Ha Thin Provinces respectively (Figure 1). Inhabiting exclusively in main channels of rivers.

Etymology. The specific epithet refers to the known distribution of this species across China (*sino-*) and Vietnam (*-vietnamensis*).
