# Supplementary material for: Beyond Morphological Conservatism: Integrative Taxonomy Unveils Remarkable Cryptic Diversity in the Tachysurus virgatus (Oshima 1926) Group (Siluriformes: Bagridae) Endemic to the Northern Margin of East Asian Tropics
Source: Animals (Basel). 2026 Aug 5;16(15):2422. doi: 10.3390/ani16152422 (PMC13466063; doi:10.3390/ani16152422)
Supplement: Supplementary file 1 [file animals-16-02422-s001.zip › Supplementary File S2.pdf]

Table S1. Morphomertric data for *T. pesudovirgatus* sp. nov. and *T. virgatus* s. str..

	Tachysurus pseudovirgatus sp. **nov.**			***Tachysurus virgatus s. str.***	
	Holotype	Paratypes (n=7)		Topotypes (n=31)	
		Range	Mean±SD	Range	Mean±SD
Standard length	57.3	52.8-61.2	56.0±2.1	51.62-83.1	64.2±7.8
%SL					
Body depth at anus	18.1	18.1-19.6	18.6±0.7	18.3-20.0	18.6±0.7
Predorsal length	40.3	37.1-40.1	39.4±1.2	34.5-40.9	37.2±1.4
Preanal length	68.7	63.8-69.1	67.0±1.9	60.8-69.9	64.3±1.7
Prepelvic length	56.9	53.2-57.9	55.3±1.7	48.6-54.5	51.8±1.6
Length of dorsal-fin spine	20.9	18.8-22.1	20.3±1.2	15.7-27.1	21.3±3.7
Length of dorsal-fin base	16.2	14.1-17.9	15.9±1.4	12.4-17.3	14.4±1.1
Length of pectoral-fin spine	19.8	18.7-20.8	19.5±0.9	16.4-23.3	19.0±1.4
Length of anal-fin base	18.5	18.0-21.2	19.2±1.1	16.7-22.7	19.8±1.3
Adipose to caudal distance	19.4	17.4-20.2	19.3±0.9	15.3-21.0	18.2±1.5
Length of caudal peduncle	19.5	17.7-19.9	18.6±0.8	14.9-19.2	17.0±1.1
Depth of caudal peduncle	10.2	9.4-10.8	10.3±0.5	9.5-12.1	10.7±0.7
Head length at latera	27.9	26.2-28.8	27.3±1.0	25.2-28.8	26.8±1.0
%HL					
Head depth	=61.1	58.6-63.8	62.1±1.8	58.7-73.6	62.9±4.6
Head width	68.9	67.4-73.7	70.3±2.0	66.4-76.7	68.5±3.8
Snout length					
Interorbital width	33.9	32.0-42.9	35.4±3.6	28.8-42.4	36.3±3.2
Eye diameter	27.9	27.5-31.8	29.1±1.8	23.1-33.6	28.5±2.6
Mouth width	38.6	38.6-44.4	40.5±2.1	34.0-50.4	40.0±3.4
Length of nasal barbel	37.5	35.5-50.4	42.0±5.3	32.2-58.3	45.7±6.4

Length of maxillary barbel	76.1	74.9-85.7	80.0±3.8	67.1-92.1	85.6±6.1
Length of inner mandibular barbel	31.8	31.4-36.6	33.3±1.8	28.9-42.5	35.3±2.8
Length of outer mandibular barbel	54.2	46.4-56.2	52.2±3.3	42.2-56.6	50.7±3.4

Table S2. Morphomertric data for *T. trimaculatus* sp. nov.，*T. bailongensis* sp. nov. and *T. furcatus* sp. nov.

	***Tachysurus trimaculatus sp.* nov.**			***Tachysurus bailongensis sp.* nov.**			***Tachysurus furcatus sp.* nov.**		
	Holotype	Paratypes (n=14)		Holotype	Paratypes (n=11)		Holotype	Paratypes (n=12)	
		Range	Mean±SD		Range	Mean±SD		Range	Mean±SD
Standard length	66.9	46.2-69.6	56.7±6.9	71.0	42.3-76.8	65.5±9.8	71.4	52.7-74.4	64.5±7.2
%SL									
Body depth at anus	17.6	14.0-18.4	16.5±1.2	20.2	16.4-21.3	19.2±1.3	21.5	18.3-21.5	20.0±1.2
Predorsal length	38.3	35.1-40.2	37.5±1.6	39.3	34.8-40.6	37.1±1.7	37.4	33.3-40.8	37.5±2.1
Preanal length	65.0	61.8-68.4	64.3±1.9	69.9	63.8-70.0	66.8±2.4	66.5	61.4-68.3	65.4±1.8
Prepelvic length	49.8	48.3-55.3	51.8±1.8	54.6	49.6-58.2	53.8±2.6	54.7	50.2-58.8	53.0±1.8
Length of dorsal-fin spine	18.9	15.7-19.4	18.1±1.1	22.6	19.7-23.5	21.3±1.1	19.2	18.8-22.3	19.7±1.1
Length of dorsal-fin base	13.4	12.6-15.7	13.9±0.9	13.9	12.2-16.4	14.6±1.3	14.1	13.1-15.6	14.3±0.8
Length of pectoral-fin spine	17.7	14.8-19.2	17.6±1.0	18.8	16.6-20.9	18.6±1.3	19.7	17.6-20.7	19.2±0.9
Length of anal-fin base	17.2	16.9-22.0	19.3±1.5	18.7	16.6-20.0	18.6±1.0	18.5	16.5-19.4	17.9±0.8
Adipose to caudal distance	17.4	17.1-20.3	18.6±1.0	18.5	16.1-18.9	17.6±1.1	18.6	16.9-20.1	18.2±1.0
Length of caudal peduncle	19.2	16.0-21.2	17.9±1.2	16.9	15.7-18.4	16.9±0.9	17.3	16.5-19.3	17.6±0.8
Depth of caudal peduncle	10.1	8.4-10.2	9.4±0.5	11.2	9.7-12.0	10.6±0.7	12.1	11.1-12.6	11.8±0.5
Head length at latera	26.7	23.9-27.7	25.8±1.0	27.2	23.4-27.5	25.9±1.2	26.8	24.6-29.1	26.9±1.2
%HL									
Head depth	65.1	55.8-66.0	62.1±2.8	55.9	53.4-59.1	57.4±2.1	68.9	59.5-71.1	63.3±3.2
Head width	70.5	66.8-76.4	69.8±4.2	63.3	59.8-68.5	64.2±2.7	73.8	65.7-74.6	70.1±2.4

Snout length									
Interorbital width	39.4	30.7-41.0	37.0±3.1	34.2	29.0-37.7	34.3±2.8	35.0	34.6-42.2	37.6±2.5
Eye diameter	31.4	24.6-31.5	28.7±2.0	23.1	22.8-31.8	28.8±2.8	29.8	23.8-35.4	29.0±2.8
Mouth width	40.3	36.8-45.5	41.1±2.0	41.4	34.9-44.0	40.1±2.6	29.8	33.8-42.0	38.9±2.4
Length of nasal barbel	42.7	36.3-54.6	42.7±5.3	38.4	32.5-52.9	42.0±6.8	50.3	35.9-58.8	42.3±2.4
Length of maxillary barbel	63.8	63.0-82.3	72.6±5.8	73.4	67.2-85.5	78.6±6.1	74.8	66.7-89.1	76.7±8.5
Length of inner mandibular barbel	31.6	25.8-37.7	31.8±3.4	30.7	28.5-40.0	32.8±4.2	35.1	30.2-38.9	33.8±3.2
Length of outer mandibular barbel	41.0	39.5-58.8	48.8±5.8	43.8	41.1-56.9	47.8±5.3	47.7	39.0-57.1	48.3±5.9

Table S3. Morphomertric data for *T. platycephalus* sp. nov. and *T. heterofasciatus* sp. nov.

	Tachysurus platycephalus sp. **nov.**			***Tachysurus heterofasciatus sp.*** **nov.**		
	Holotype	Paratypes (n=7)		Holotype	Paratypes (n=10)	
		Range	Mean±SD		Range	Mean±SD
Standard length	79.9	34.2-137.5	79.6±38.5	156.9	57.8-156.9	106.2±32.6
%SL						
Body depth at anus	14.4	14.1-17.5	16.1±1.4	15.4	14.5-16.7	15.4±0.6
Predorsal length	38.1	35.1-41.1	37.6±2.0	37.3	33.4-39.5	36.3±1.7
Preanal length	63.5	58.5-66.2	62.3±2.6	64.6	60.8-65.3	63.5±1.3
Prepelvic length	51.3	47.3-56.8	51.2±3.3	50.6	49.5-53.4	50.9±1.2
Length of dorsal-fin spine	17.9	15.7-20.3	17.7±1.9	20.0	19.1-24.4	21.6±1.9
Length of dorsal-fin base	13.6	10.5-15.3	12.8±1.5	12.3	12.1-15.3	13.1±0.9
Length of pectoral-fin spine	17.1	14.8-19.9	17.6±1.7	19.2	18.2-24.8	20.5±1.8
Length of anal-fin base	21.8	21.6-22.1	21.4±0.6	21.0	19.3-22.6	21.0±1.1
Adipose to caudal distance	19.2	16.8-20.7	18.6±1.2	19.4	17.0-19.4	18.1±0.8

Length of caudal peduncle	17.0	16.7-17.9	17.3±0.4	17.1	14.8-20.3	17.8±1.5
Depth of caudal peduncle	8.3	7.8-9.3	8.7±0.5	7.8	7.7-9.3	8.2±0.5
Head length at latera	26.6	23.9-30.9	26.7±2.5	25.0	23.3-29.6	26.3±1.9
%HL						
Head depth	53.6	51.1-65.1	57.7±4.6	60.2	50.8-63.7	57.0±4.0
Head width	65.3	64.8-75.0	70.4±4.3	71.1	62.3-77.3	69.3±4.2
Snout length						
Interorbital width	34.3	32.2-40.4	36.7±3.2	38.2	36.0-41.2	37.9±1.8
Eye diameter	23.3	20.0-30.2	24.6±3.4	18.2	18.2-31.6	24.6±3.9
Mouth width	46.1	45.1-54.0	49.5±3.3	41.4	39.7-52.3	44.0±3.9
Length of nasal barbel	30.5	27.2-46.2	35.6±6.7	43.1	36.3-50.1	42.9±4.3
Length of maxillary barbel	62.0	56.2-72.1	60.5±6.1	67.3	57.9-76.1	66.7±5.9
Length of inner mandibular barbel	32.1	25.0-34.3	31.2±3.2	35.3	25.3-39.0	31.5±4.3
Length of outer mandibular barbel	42.2	34.9-54.2	45.9±5.9	58.9	44.0-59.2	50.7±5.4

Table S4. Morphomertric data for *T. analimaculatus* sp. nov., *T. qiongzhouensis* sp. nov. and *T. sinovietnamensis* sp. nov.

	***Tachysurus analimaculatus sp.* nov.**			***Tachysurus qiongzhouensis sp.* nov.**			***Tachysurus sinovietnamensis sp.* nov.**		
	Holotype	Paratypes (n=17)		Holotype	Paratypes (n=18)		Holotype	Paratypes (n=13)	
		Range	Mean±SD		Range	Mean±SD		Range	Mean±SD
Standard length	99.8	41.0-105.9	67.3±22.3	77.1	56.6-98.3	72.3±11.9	63.33	50.0-83.2	64.6±8.6
%SL									
Body depth at anus	19.1	13.8-19.2	17.8±2.3	19.7	19.8-22.1	20.1±1.9	18.9	19.1-24.5	20.8±2.2
Predorsal length	37.4	32.3-40.6	37.4±2.1	37.8	34.8-39.8	37.3±1.4	36.3	35.2-41.1	37.7±1.6
Preanal length	65.0	59.3-68.6	64.8±2.6	66.3	59.8-68.4	64.5±2.7	59.8	59.6-69.3	63.8±2.9
Prepelvic length	52.9	48.8-55.5	52.4±2.0	53.9	51.3-55.6	53.1±1.4	50.7	48.1-55.1	51.3±2.4

Length of dorsal-fin spine	20.6	19.1-24.9	21.7±1.9	19.1	18.3-23.9	21.6±1.7	21.7	16.3-25.9	21.6±2.4
Length of dorsal-fin base	14.6	12.5-16.0	14.5±1.0	13.1	11.9-16.5	14.0±1.2	13.3	12.7-15.6	14.0±1.0
Length of pectoral-fin spine	19.4	17.8-22.4	19.9±1.3	15.8	14.2-20.7	18.0±2.0	20.1	17.2-20.7	19.0±1.0
Length of anal-fin base	18.9	17.3-23.9	20.4±1.6	20.7	19.1-24.6	21.7±1.3	26.7	19.7-27.7	22.2±2.1
Adipose to caudal distance	18.3	17.2-19.7	18.6±0.9	17.4	16.0-20.4	18.3±1.5	17.1	16.5-20.1	18.3±1.1
Length of caudal peduncle	15.1	14.6-19.5	16.7±1.6	14.8	14.3-20.5	17.1±1.8	14.6	14.5-19.1	16.7±1.2
Depth of caudal peduncle	10.1	7.9-10.2	9.5±0.7	10.7	10.3-11.9	10.9±0.6	9.8	9.3-11.8	10.5±0.8
Head length at latera	23.8	23.4-29.9	26.4±1.9	26.0	24.1-28.8	26.6±1.2	25.0	23.8-28.3	25.6±1.1
%HL									
Head depth	68.7	55.9-74.1	64.8±5.7	62.5	61.1-71.5	66.7±3.1	60.5	57.1-73.6	62.6±4.4
Head width	67.9	57.5-79.5	66.1±5.2	62.2	59.0-74.0	66.4±4.7	65.4	57.5-68.8	64.0±3.8
Snout length									
Interorbital width	42.8	30.9-43.6	37.4±3.9	35.4	32.0-42.7	39.8±2.8	39.5	31.5-47.4	38.3±4.2
Eye diameter	31.3	24.3-36.3	29.8±3.1	23.9	23.6-34.8	27.4±2.9	25.8	25.0-35.7	28.3±2.0
Mouth width	39.5	34.8-41.9	37.4±2.0	35.2	32.9-43.8	37.7±2.9	38.0	32.9-44.1	37.9±2.8
Length of nasal barbel	40.5	30.6-44.8	38.1±5.0	60.5	44.0-69.8	56.7±9.3	39.8	35.3-57.9	41.1±4.4
Length of maxillary barbel	68.6	59.8-77.7	67.4±4.6	69.6	64.8-89.0	76.0±7.7	69.0	59.6-78.1	67.5±5.9
Length of inner mandibular barbel	28.9	22.8-34.7	28.5±3.5	27.4	25.4-35.0	25.9±3.0	25.9	23.1-31.4	27.1±2.7
Length of outer mandibular barbel	47.3	35.0-47.4	41.2±3.6	43.4	36.1-56.4	45.5±5.8	38.4	34.6-49.7	41.7±3.9
